# Synthesis of BODIPY-pyrrolo[3,4-*b*]pyridin-5-ones *via* Ugi-Zhu/cascade reactions and studies of fluorescence response toward viscosity

**DOI:** 10.3389/fchem.2024.1488933

**Published:** 2024-10-18

**Authors:** Julio C. Flores-Reyes, Annia Galano, Sandra M. Rojas-Montoya, Luis Blancarte-Carrazco, Elba Xochitiotzi-Flores, Héctor García-Ortega, Norberto Farfán, Alejandro Islas-Jácome, Eduardo González-Zamora

**Affiliations:** ^1^ Departamento de Química, Universidad Autónoma Metropolitana-Iztapalapa, Ciudad de México, Mexico; ^2^ Departamento de Química Orgánica, Facultad de Química, Universidad Nacional Autónoma de México, Ciudad de México, Mexico

**Keywords:** multicomponent reaction, BODIPY, fluorescence, viscosity, polyheterocycle

## Abstract

A series of seven new *meso*-phenyl BODIPY-pyrrolo[3,4-*b*]pyridin-5-one conjugates were synthesized in one experimental step by using a Sc(III)-catalyzed Ugi-Zhu three-component reaction coupled to a cascade sequence (*aza* Diels-Alder/*N*-acylation/aromatization) as post-MCR functionalization process. Further experimental studies were performed behind understanding the fluorescence response toward viscosity. All compounds exhibited a linear response between increasing viscosity (DMSO and glycerol mixtures) and fluorescence intensity. The different substituents also influenced the photophysical properties. Furthermore, in DMSO all compounds exhibited dual emission. Each band is attributed to the pyrrolo[3,4-*b*]pyridin-5-one and BODIPY moieties, respectively. The electronic structure of all compounds was computed by DFT and TD-DFT calculations, allowing to determine the molecular orbitals involved in the electronic transitions.

## Introduction

Fluorescent viscosity probes have emerged as important tools to monitor the microenvironment of several systems of interest in various fields, for instance, the inside of cells and cellular organelles. The high sensitivity in the response of fluorescence towards the change of viscosity makes the generation of these types of probes a constant research subject (Lee et al., 2018). Most fluorescent viscosity probes are fluorescent molecular rotors (FMR), which consist of fluorophores (commonly donor-acceptor structures) linked by a single bond that can adopt a twisted intramolecular charge transfer (TICT) state. The TICT model was proposed by Grabowski et al. in 1973 to explain the dual emission observed in *N*,*N*-dimethylaminobenzonitrile (DMABN), a donor-acceptor molecule (Rotkiewicz et al., 1973). In low viscosity media, the TICT state is responsible for quenching the fluorescence due to the fast rotation about the single bond, resulting in increased non-radiative relaxation pathways (Wang et al., 2021). However, in a high viscosity medium, the molecular rotation becomes gradually restricted and consequently, an increased fluorescence intensity is often observed.

**FIGURE 1:**
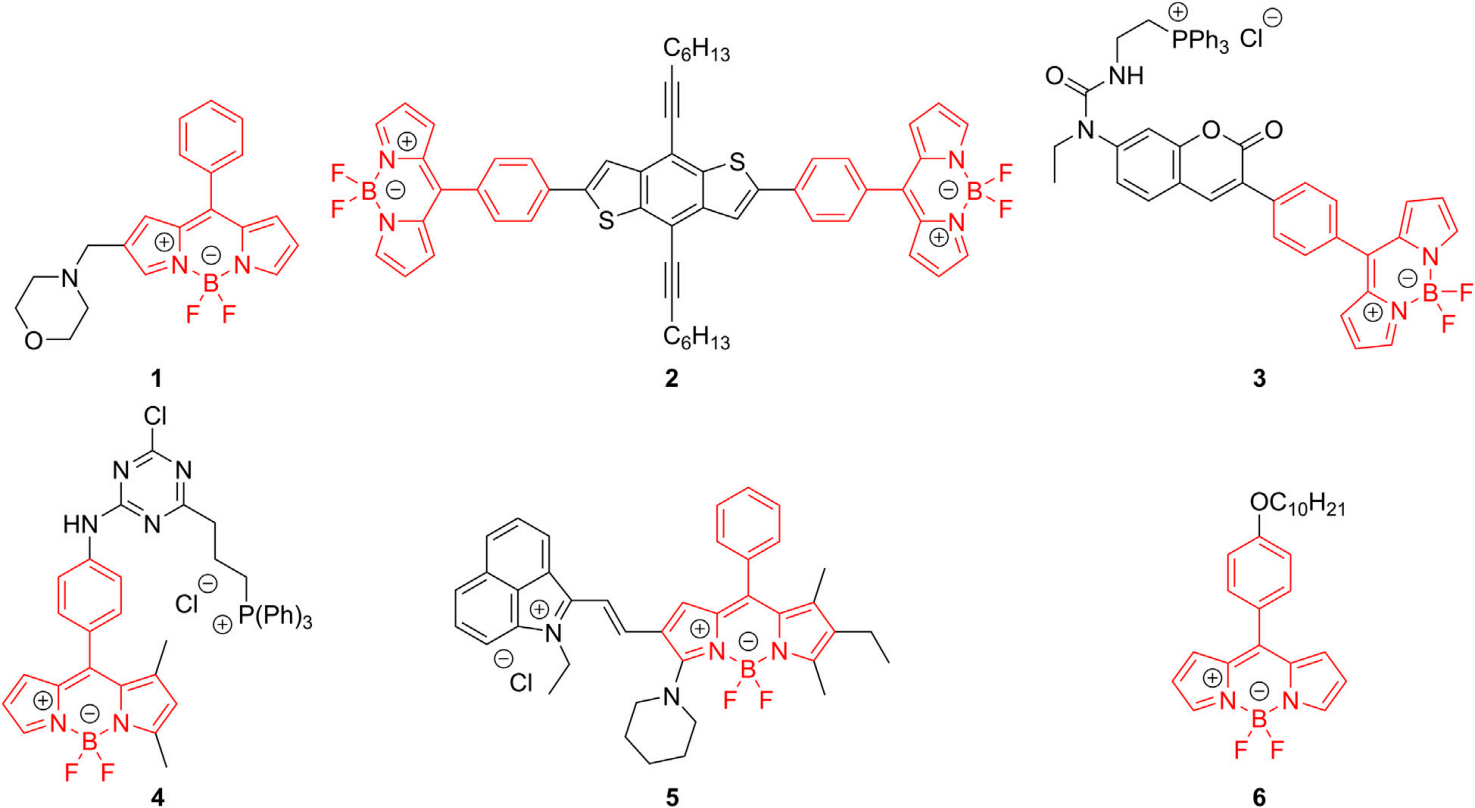
FMRs based on *meso*-phenyl BODIPY moiety.

Several classes of compounds that can act as FMRs exist, one of such are the *meso*-phenyl substituted 4,4-difluoro-4-bora-3a,4a-diaza-*s*-indacenes, better known as BODIPYs (Loudet and Burgess, 2007), particularly, those derivatives synthesized using unsubstituted pyrrole. Using 2,4-dimethylpyrrole for the synthesis of *meso*-phenyl BODIPY is a common strategy to increase the fluorescence quantum yield (FQY) because the rotation of the *meso*-phenyl substituent becomes sterically hindered, thus favoring the radiative pathways. This, however, comes with a decreased environmental sensitivity towards, for example, solvent polarity, viscosity and temperature. These compounds have gained increased attention due to their photophysical properties such as high molar extinction coefficients, visible to red emission wavelengths, and high FQYs, tunable with relative ease through chemical modifications (Miao et al., 2019). Furthermore, the low toxicity and excellent photostability exhibited by BODIPY-type dyes make them ideal candidates for diverse applications such as biological imaging, fluorescent probes, and chemical tags for different targets (Boens et al., 2012). BODIPY derivatives have experienced a widespread adoption as fluorescent probes towards different targets. As was mentioned above, they have been used as FMRs to sense changes in viscosity, and even have been used as intracellular viscosity probes (Su et al., 2014; Zhu et al., 2014; Su et al., 2016) since it has been demonstrated that abnormal changes in the intracellular viscosity can be related to some diseases such as Alzheimer’s disease, or diabetes. By adding suitable functional groups to the BODIPY structure it has been used as a sensor of H_2_S by reduction of the azide group (Saha et al., 2013) or thiolysis of dinitrophenyl ether (Cao et al., 2012) appended to the structure; it has been used for sensing a wide variety of metal cations such as Mg(II) (Lin, et al., 2016), Al(III) (Kashyap et al., 2019), Fe(III) (Nootem, et al., 2021), Cu(II) (Xue, et al., 2016) and Zn(II) (Zhu et al., 2013), to name a few. Furthermore, BODIPY dyes have been used as constituents of the emissive material layer in green (Song et al., 2020; Nakamura et al., 2023), red (Ma et al., 2022) and near infrared (Shikano et al., 2021) OLEDs. This versatility of applications makes the development of novel fluorescent probes based on BODIPY a constant research interest. Figure 1 shows some examples of FMRs based on the BODIPY core.

Molecules incorporating more than one non-conjugated chromophoric fragment are referred to as multichromophore systems. These compounds do not interact in the ground state due to the lack of conjugation but, they can interact in the excited state. One of the main rationales behind the synthesis of multichromophore systems is that their emission colors can be additively mixed, which could potentially be used to obtain white light-emitting materials; this, however, remains quite challenging (Grotkopp et al., 2018). A synthetic tool that appears to be custom-made for the synthesis of multichromophore systems are multicomponent reactions (MCRs), which involve combinations of at least three structurally different reactants into a domino one-pot process to generate a product containing all or most of the atoms from the starting materials (high atom economy). MCRs coupled to further functionalization processes are especially suited for the rapid construction of complex polyheterocycles with diverse functionalities (Levi and Müller, 2016; Ibarra et al., 2018; Rocha et al., 2020).

In this context, our research group continues to advance the development of the Ugi-Zhu reaction (Flores-Reyes et al., 2021) coupled to a cascade process for the synthesis of *bis*-chromophoric pyrrolo[3,4-*b*]pyridin-5-ones. As a continuation of our previous study involving *meso*-thienyl BODIPY-pyrrolo[3,4-*b*]pyridin-5-ones (Flores-Reyes et al., 2024), we now explored a different variant with a *meso*-phenyl substituent. Phenyl substituents at the *meso* position typically adopt an orthogonal conformation resulting in a poor electronic coupling. With this in mind, we expect the BODIPY moiety to become more isolated from remote electronic effects and in turn exhibit a good environmental response, in this case towards varying viscosity. Herein we report the one-pot multicomponent synthesis of *meso*-phenyl BODIPY-pyrrolo[3,4-*b*]pyridin-5-one conjugates and the study of their fluorescence response in a high viscosity medium using UV-Vis and fluorescence spectroscopies, while the involved electronic transitions were computed by DFT and TD-DFT calculations. These new products were obtained in a single experimental step with moderate yields. With this work we aim to continue expanding the available synthetic methodologies to access multichromophore systems with diverse photophysical properties through a multicomponent approach.

## Results and discussion

### Synthesis

BODIPY-containing aldehyde **7** was prepared according to a procedure reported in the literature (Xochitiotzi-Flores et al., 2016). After this, the next step consisted of the synthesis of the key α-isocyanoacetamide **9** to perform the Ugi-Zhu multicomponent reaction. This latter one was prepared also according to a method reported in the literature (Fayol et al., 2005) but with a slight modification. Once these key reagents were obtained, we continued with the synthesis of the target polyheterocyclic scaffold containing the BODIPY moiety.

**SCHEME 1:**
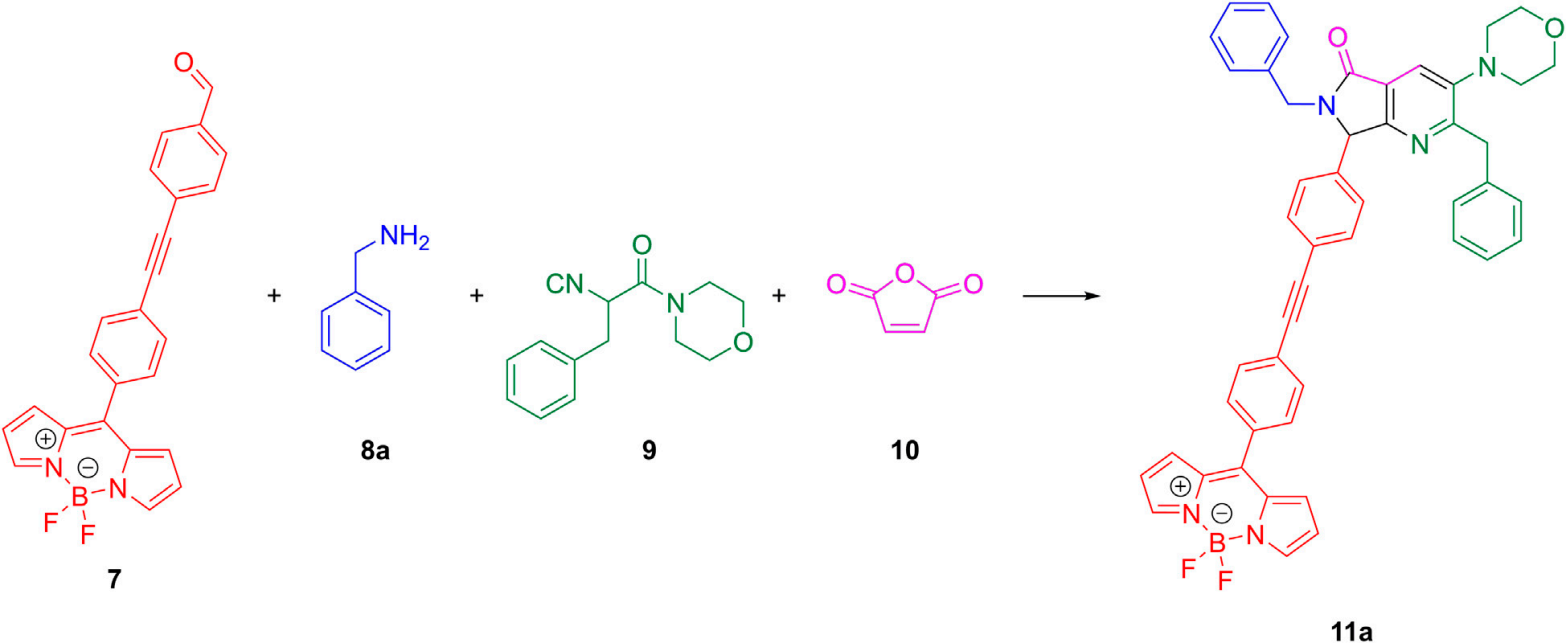
Model reaction to test for the optimal reaction conditions.

Due to the novel characteristics of the aldehyde that was utilized in the preparation of the compounds, it was necessary to carry out a screening of the conditions for the MCR to obtain acceptable yields using a reaction model. To do this, the *meso*-(5-((4-formylphenyl)ethynyl)phenyl)-4,4-difluoro-4-bora-3a,4a-diaza-*s*-indacene (**7**), benzylamine (**8a**), 2-isocyano-1-morpholino-3-phenylpropan-1-one (**9**) and maleic anhydride (**10**) were combined sequentially to synthesize the corresponding BODIPY-pyrrolo[3,4-*b*]pyridin-5-one **11a**, according to Scheme 1.

Table 1 shows the reaction conditions that were evaluated. First, the reaction conditions originally reported by Zhu (Janvier et al., 2002) were assayed (entry 1). However, even after 24 h of stirring, there was no change in the progress of the reaction. Microwave heating (entry 2) and the addition of a weak Brønsted acid (entry 3) were also unsuccessful to promote the reaction. Next, the non-polar solvent toluene (PhMe) was used (entry 4), in which the solubility of the aldehyde was improved, but the reaction still did not proceed at room temperature, even when microwave heating was applied (entry 5). A catalytic amount of scandium(III) triflate was utilized next (entry 6), as it has proved successful at promoting this reaction in previous reports of our research group (Zamudio-Medina et al., 2010; Vázquez-Vera et al., 2017; Zamudio-Medina et al., 2018; Segura-Olvera et al., 2019) and others (Rentería-Gómez et al., 2019; Pharande et al., 2020; Longo Jr et al., 2021). As expected, the addition of this Lewis’ acid promoted the reaction even at room temperature, albeit with a prolonged reaction time. The yield increased slightly when using microwave heating (entry 7). A two-fold increase of the catalyst loading improved the yield substantially (entry 8). A higher temperature (entry 9), however, did not improve the yield. Other Lewis’ acids were employed, namely, ytterbium(III) triflate (entry 10), which produced a lower yield than scandium(III) triflate, and indium(III) chloride (entry 11), which failed to promote the reaction at all. These Lewis’ acids were selected because they are considered as hard acids (small ionic radii, high oxidation state), they are stable in the presence of water and air, and, with the exception of indium(III) chloride, they have a weak coordinating counterion (OTf) which will not compete with the imine for coordination with the cation. Furthermore, the latter may be the reason why indium(III) chloride failed to promote the reaction.

**TABLE 1:** Optimization of reaction conditions.

Entry	Solvent	Temperature	Additive	Yield (%)
1	MeOH	RT	—	—
2	MeOH	MW (60°C)	—	—
3	MeOH	MW (60°C)	NH_4_Cl (1.5 equiv.)	—
4	PhMe	RT	—	—
5	PhMe	MW (60°C)	—	—
6	PhMe	RT	Sc(OTf)_3_ (5 mol%)	26
7	PhMe	MW (60°C)	Sc(OTf)_3_ (5 mol%)	30
8	PhMe	MW (60°C)	Sc(OTf)_3_ (10 mol%)	46
9	PhMe	MW (80°C)	Sc(OTf)_3_ (10 mol%)	45
10	PhMe	MW (60°C)	Yb(OTf)_3_ (5 mol%)	19
11	PhMe	MW (60°C)	InCl_3_ (5 mol%)	—
12	MeOH	RT	Sc(OTf)_3_ (10 mol%)	—
13	MeOH	MW (60°C)	Sc(OTf)_3_ (10 mol%)	8

Having established scandium(III) triflate as the best catalyst for the MCR, we revisited the use of MeOH as a solvent in the presence of this catalyst at RT and at 60°C. At RT (entry 12) the reaction did not proceed, and product formation was not observed. At 60°C (entry 13) the reaction did occur and **11a** was obtained in 8% yield. This result was attributed to the reduced solubility of **7** in MeOH and possibly to the ring opening and esterification of maleic anhydride in the presence of MeOH.

**FIGURE 2:**
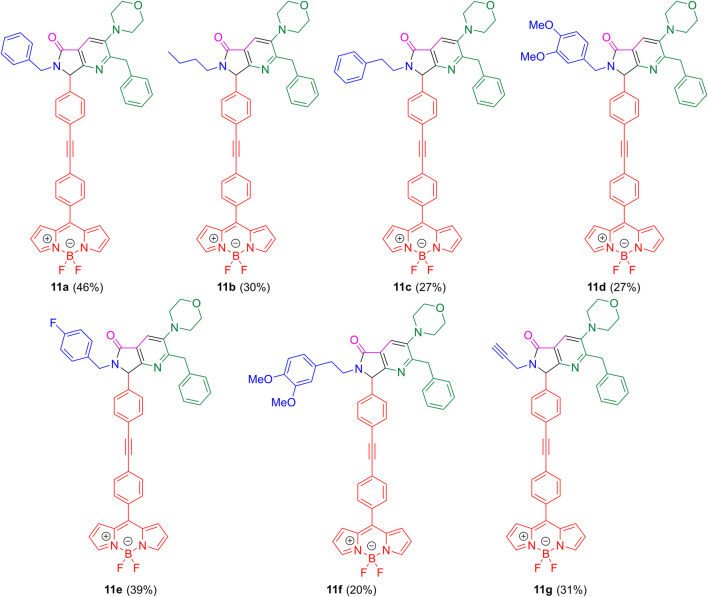
Synthesized compounds under the optimized reaction conditions.

Once the optimal reaction conditions were established (PhMe as the solvent, MW as heat source, at 60°C, and using a loading of 10 mol% of Sc(III) as the catalyst), the synthesis of the compound library **11a-g** was carried out using the BODIPY-functionalized aldehyde **7**, seven alkyl amines **8a-g**, the α-isocyanoacetamide **9** and maleic anhydride (**10**) (Figure 2). Similar yields were obtained for all compounds which ranged from 20% to 46%. These yields are acceptable considering the structural complexity of the products, the number of reaction steps that occur and the remarkable atom economy (AE > 90% for all the cases) because only a couple of molecules of water and one molecule of carbon dioxide were released per synthesized compound. The reason for the moderate yields was due to incomplete consumption of the starting aldehyde and imine intermediate even under the optimized reaction conditions. Complete conversion of 5-aminooxazole to pyrrolo[3,4-*b*]pyridine-5-one was observed in all cases, according to the TLC monitoring. Formation of byproducts was not observed during the course of the reactions, even when using 3,4-dimethoxyphenethylamine (**11f**), which could potentially give the Pictet-Spengler product. This is in agreement with the original report by J. Zhu (Janvier et al., 2002) in which it is stated that the intermolecular reaction between imine and isocyanide is faster than the intramolecular trapping to form the corresponding tetrahydroisoquinoline. The overall atom economy of the process is remarkable considering that five new bonds were formed, even if the yields were low. However, it is important to note that atom economy is not an optimal parameter to determine whether a chemical reaction is efficient or not since it fails to take into account the incomplete conversion of reactants into products or formation of byproducts. The structure of all synthesized compounds was elucidated by NMR experiments (^1^H, ^13^C, ^11^B and ^19^F) and high-resolution mass spectrometry (HRMS). Assignments for all protons and carbons were performed by 2D NMR (COSY, HSQC and HMBC). See the Supplementary Material for further details.

### Absorption and emission spectra

**FIGURE 3:**
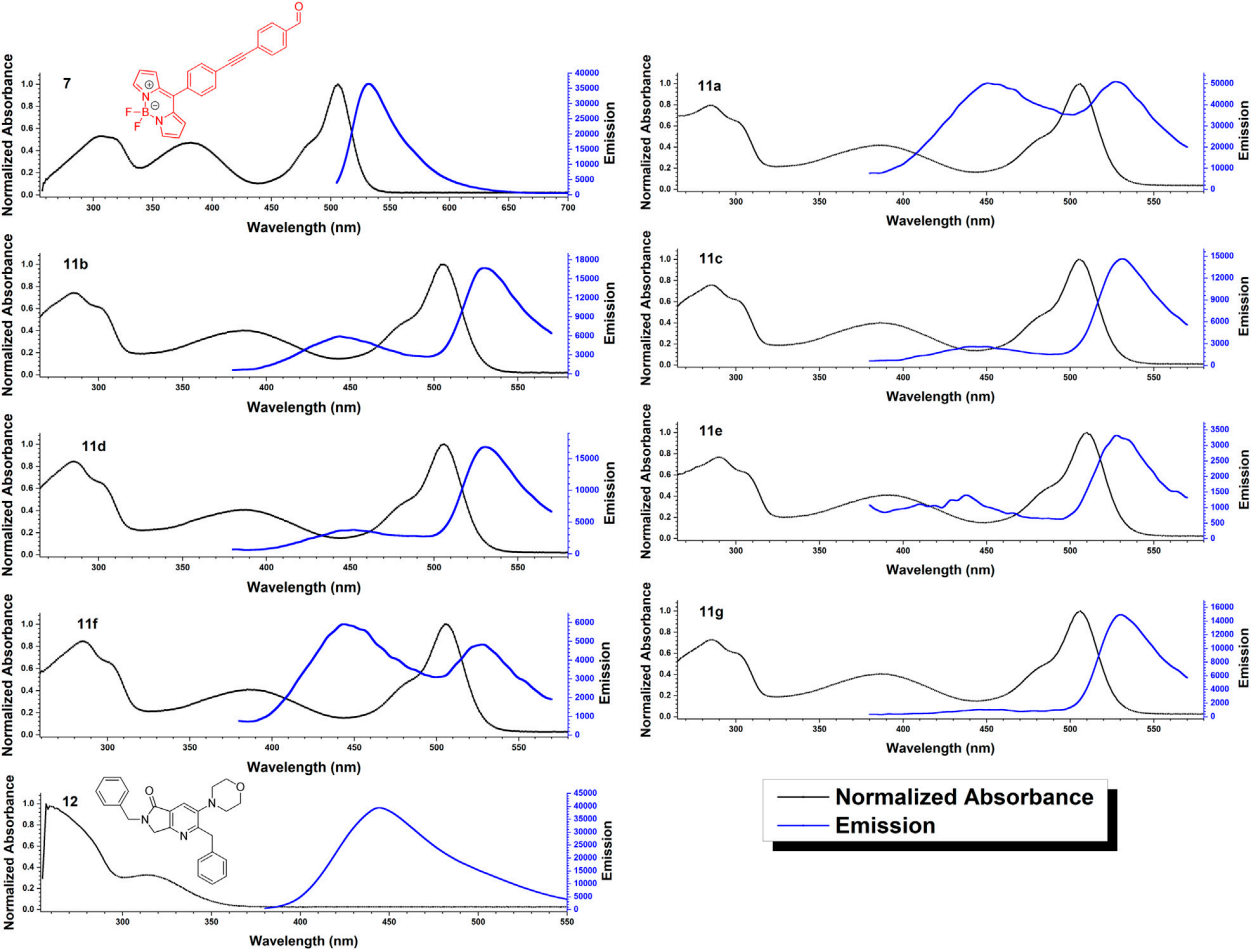
Normalized absorption and emission spectra (Excitation at 290 nm) for the studied compounds. DMSO was used as solvent at a concentration of 10^–6^ M at RT.

The absorption and emission spectra of all compounds were acquired using dimethyl sulfoxide (DMSO) as the solvent, at a concentration of 10^–6^ M and at room temperature. These spectra are detailed in Figure 3. Additionally, Table 2 summarizes the optical properties of each compound. All studied compounds exhibit absorption maxima at around 505 nm, and a second absorption band at around 390 nm, corresponding to the S_0_→S_1_ and S_0_→S_2_ transitions of the BODIPY core, respectively (Rojas-Montoya, et al., 2024). Additionally, there is an absorption band near 285 nm attributed to the pyrrolo[3,4-*b*]pyridin-5-one core (Flores-Reyes, et al., 2024). Compound **11e**, which features a *p*-fluorobenzyl group in its structure, shows a bathochromic shift of approximately 5 nm for each of these bands. It is important to highlight that the substituent present in the pyrrolo[3,4-*b*]pyridin-5-one core influences its optical properties. For example, in the case of compound **11b**, the aliphatic chain caused a decrease in its molar absorptivity coefficient (ε).

**TABLE 2:** Summary of optical properties using DMSO as the solvent.

Compound	Absorbance		Emission		
	λ_max_ (nm)^A^ (ε) [L mol^-1^ cm^-1^]	λ_max_ (nm)^B^ (ε) [L mol^-1^ cm^-1^]	λ_max_ (nm)^A^	λ_max_ (nm)^B^	FQY
7	—	506 (58,029)	—	532	0.0066
11a	285 (22,460)	506 (32,353)	450	528	0.0002
11b	285 (8,006)	505 (12,135)	444	530	0.0026
11c	285 (22,469)	505 (30,642)	445	531	0.1715
11d	285 (21,671)	505 (25,735)	449	531	0.0042
11e	290 (29,979)	510 (41,063)	438	528	0.0006
11f	286 (29,212)	506 (35,358)	444	528	NP
11g	286 (31,237)	506 (43,491)	449	530	NP
12	260(20,900)	—	445	—	NP

Notes: ^A^ Corresponding to the pyrrolo[3,4-*b*]pyridin-5-one core. ^B^ Corresponding to the BODIPY core.

In the emission spectra, two bands are generally observed that correspond to each chromophore. The characteristic emission of the *meso*-phenyl BODIPY core is seen between 528 and 531 nm, while the emission of the pyrrolo[3,4-*b*]pyridin-5-one core appears between 438 and 450 nm, with a more pronounced hypsochromic shift in compound **11e**. To support this statement, we acquired the absorption and emission spectra of an unsubstituted analog of **11a** (**12**) in DMSO and compared them to the spectra of **7**. The emission spectra of the dyads correspond to the overlapping of the emission spectra of both moieties. The absorbance spectra of dyads **11a-g** show a more intense band between 250 and 300 nm than the one shown for compound **7**, which corresponds to the pyrrolo[3,4-*b*]pyridin-5-one’s absorption. Emission of BODIPY **7** is shown in Supplementary Figure S40. The relative intensity of these bands varies among the different compounds. Generally, the emission from the BODIPY core is stronger. However, for compound **11f**, the intensity of these two bands is similar. Notably, compound **11e** shows a higher emission intensity of the pyrrolo[3,4-*b*]pyridin-5-one band. This dual emission centered into two different fluorophores allows organic compounds to cover a wide range of the visible spectrum and has awakened interest for designing white organic LEDs (Lee et al., 2020). Some examples of dual emitting organic materials containing the BODIPY core are the BODIPY-(1,8-naphtilamide) dyads separated by an oxoaryl bridge which showed the corresponding emissions for each fluorophore (Murkherjee and Thilagar, 2014), and phenyleneethynylene-BODIPY oligomers, which showed dual emission only when the oligomer chain was long enough to achieve an energy transfer quench (Reyes-Flores et al., 2021).

It is worth mentioning that the excitation spectra of compounds **11a-g** showed a contribution of the pyrrolo[3,4-*b*]pyridin-5-one absorption towards the BODIPY emission (Supplementary Figure S41, measured at 540 nm). While BODIPY **7** exhibits absorption around 320 nm, dyads exhibit a band near 290 nm, this agrees with the computational studies shown below where the lowest energy transition has a charge transfer character contribution. Excitation spectra of the pyrrolo[3,4-*b*]pyridin-5-one is shown in Supplementary Figure S42.

Compared to our previous study where we synthesized *meso*-thienyl BODIPY-pyrrolo[3,4-*b*]pyridine-5-one conjugates (Flores-Reyes, et al., 2024), the absorption maxima of both chromophores are slightly blue shifted. The bathochromic shift in the *meso*-thienyl derivatives is attributed to a donor-acceptor structure arising from the inclusion of the thiophene moiety, a weak electron donor. The FQYs are very low and about the same in both series of compounds, which was expected due to the unrestricted rotation of the BODIPY moiety. The most striking difference between both types of compounds lies in the emission spectra. The *meso*-phenyl series exhibit a dual emission with a significantly blue shifted emission maxima for the BODIPY core (∼110 nm), while the *meso*-thienyl counterpart exhibited two distinct emission maxima for each chromophore with different excitation wavelengths. This also highlights the dramatic effect on emission of switching the thiophene for a phenyl ring.

### Effect of viscosity on the emission

It was investigated how the emission spectra of compounds **11a-g** changed in response to varying viscosities in mixtures of dimethylsulfoxide (DMSO) and glycerol (Gly). The study began with a solution consisting entirely of DMSO, and glycerol was gradually added to increase the viscosity. The specific viscosities used are detailed in Table 3.

**TABLE 3:** Viscosity conditions utilized in the study.

DMSO:Gly proportion	100%:0	75%:25%	50%:50%	25%:75%	10%:90%	0:100%
Viscosity (mPa s)	2.2	8.5	26.8	186.3	577.4	945.0

Note: Samples were dissolved in 0.3 mL of DMSO and then 2 mL of the corresponding DMSO:Gly mixture were added.

As the viscosity of the medium increases with the addition of glycerol, all compounds exhibit an increase in the emission intensity corresponding to the BODIPY core. Similarly, the pyrrolo[3,4-*b*]pyridin-5-one core shows a hypsochromic shift, with peaks initially around 444 nm in DMSO shifting to approximately 410 nm in glycerol-containing solutions. This shift may be attributed to the stabilization of a conformer favoured by the medium’s viscosity, which affects the rotation of the triple bond between the two fluorophores. Emission spectra across various viscosities for all compounds are shown in the Supplementary Figures S43–S49. This phenomenon has been previously observed in BODIPY dimers linked by butadiyne units (Zhang et al., 2017).

The dependence of fluorescence intensity on the viscosity of the solutions was analyzed according to the Förster-Hoffmann equation (Equation 1), where I_*f*_ represents the fluorescence intensity, η is the viscosity of the medium, *c* is a constant dependent on concentration and temperature, and *χ* is a constant related to the sensitivity of the fluorophores towards viscosity (Förster and Hoffmann, 1971; Su et al., 2016).

$$\mathrm{Ln}\;I_{f}=c+\chi \ln\;\eta$$ (1)

### Calibration curves and sensitivity analysis

**FIGURE 4:**
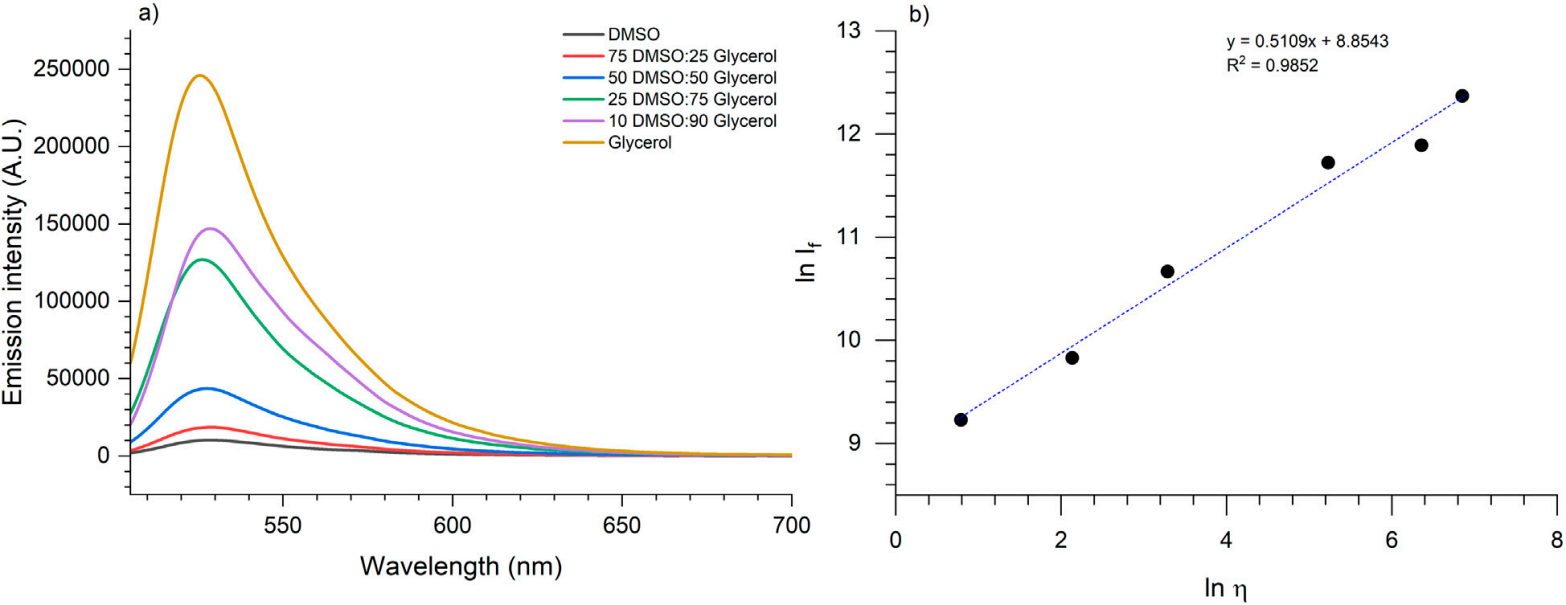
For compound 11g, excitation at 490 nm. **(A)** Variation in emission intensity with respect to viscosity (DMSO/glycerol mixtures). **(B)** Calibration curve of ln η vs. ln I_*f*_ (emission at 530 nm).

Calibration curves were obtained by performing an excitation at 400 nm for each sensor. Among them, compound **11g** exhibited the best linear trend (*R* ^2^ = 0.9852) with a coefficient *χ* = 0.511 (Figure 4). This one suggests that the presence of smaller groups attached to the nitrogen atom of the pyrrolo[3,4-*b*]pyridin-5-one reduces potential energy losses, which might occur in other analogues into this compound family due to a higher number of degrees of freedom in the substituents. To see the coefficients obtained by the Förster-Hoffman analysis of all the studied compounds see Supplementary Table S2.

The curve obtained for this analogue was compared with the one obtained when performing an excitation at 290 nm, where the emission bands of the fluorophores in the 100% DMSO solution were studied (530 nm for BODIPY and 446 nm for pyrrolo[3,4-*b*]pyridin-5-one), as well as the new band observed at 406 nm with increased viscosity (Supplementary Figure S50). The bands corresponding to the pyrrolo[3,4-*b*]pyridin-5-one core did not show a linear behavior with respect to the natural logarithm of viscosity, whereas the BODIPY band demonstrated higher sensitivity (*χ* = 0.5551) and slightly lower correlation at this excitation wavelength.

Although the emission bands of the pyrrolo[3,4-*b*]pyridin-5-one core did not exhibit a linear trend, possibly due to non-radiative decay processes and conformer formation, it is noteworthy that the behavior of all studied compounds upon excitation at 490 nm follows a linear fitting. The correlation coefficients (*R* ^2^) are at least 0.9, and the *χ* values range from 0.5027 to 0.6082, indicating a good sensitivity to changes in viscosity. This correlation suggests a systematic response among the *meso*-phenyl BODIPY-containing molecules, demonstrating their high sensitivity to variations in viscosity.

### Electronic structure

For further elucidation of the electronic transitions within compounds **11a-g**, the ground state geometries were optimized by DFT calculations using the program Gaussian 09, (Frisch et al., 2010), with the B3LYP functional and the 6-311G(d,p) basis set. The solvation model based on density (SMD) was applied using DMSO as solvent to enable a comparison with experimental results. To ensure that the optimized structures corresponded to energy minima, a frequency analysis was conducted to confirm the absence of imaginary frequencies. The optimized geometry of the compounds showed a torsion angle ranging from 53.8° to 54.2° between the BODIPY core and the phenyl substituent at the *meso* position, indicating a deviation from coplanarity (Table 4). These values are consistent with literature reports for *meso*-phenyl BODIPY compounds (Li et al., 1998).

**TABLE 4:** Dihedral angle between the BODIPY core and the *meso*-phenyl ring.

Compound	11a	11b	11c	11d	11e	11f	11g
Dihedral angle (°)	54.0	54.3	54.3	53.8	53.9	53.9	54.2

**FIGURE 5:**
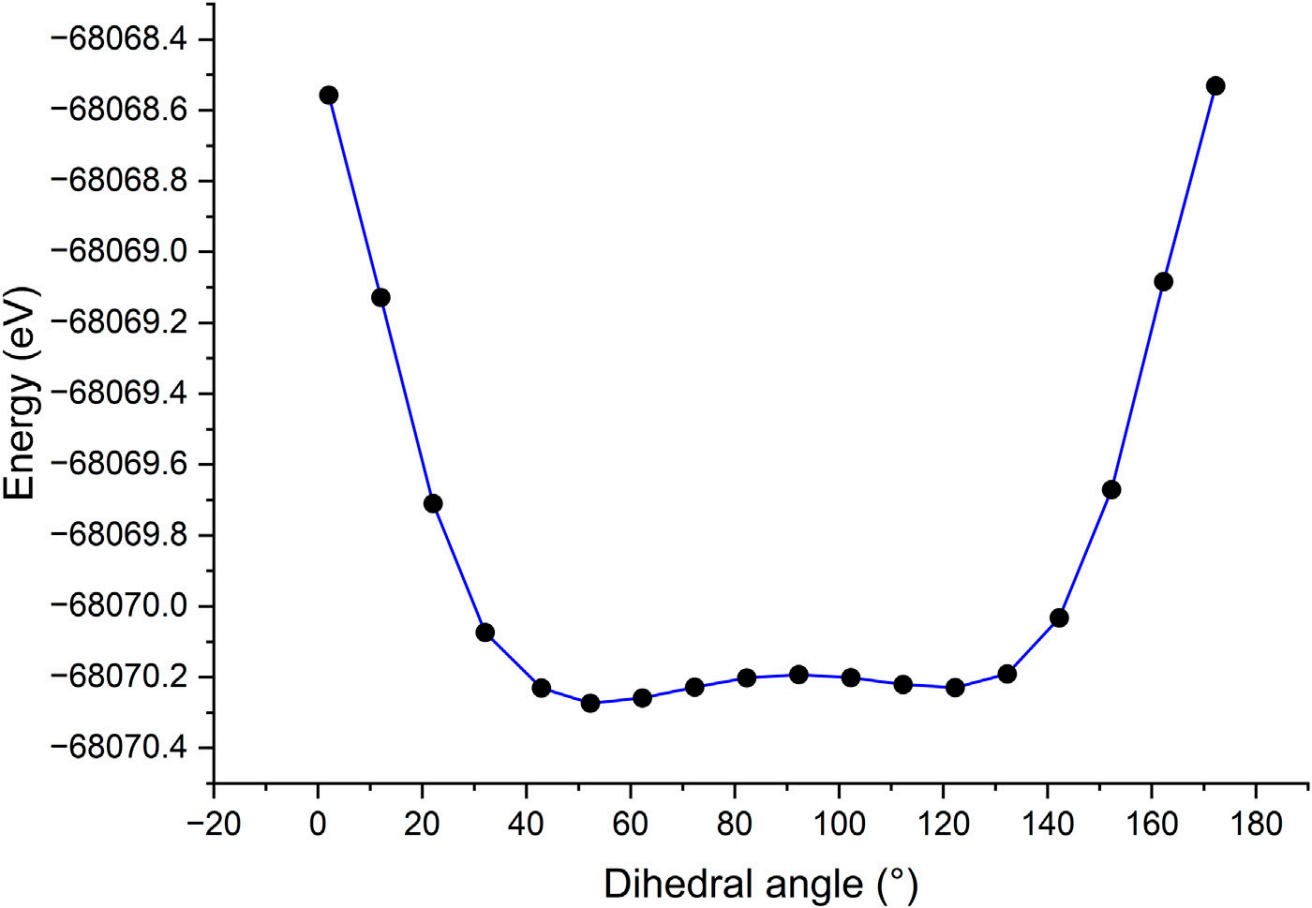
Dihedral angle scan between BODIPY and *meso*-phenyl substituent of compound **11a**.

To investigate how variations in the dihedral angle influence the energy of the compounds, a scan of the dihedral angle between the BODIPY core and the *meso*-phenyl ring was performed (Figure 5). This scan was conducted in the ground state by twisting the dihedral angle in 10° increments, covering a full range of 180°, using compound **11a** as a model. The results revealed that the rotamers with the highest energies corresponded to dihedral angles of 2° and 172°, which represent nearly coplanar conformations of the two fragments. In contrast, two energy minima were observed for rotamers at dihedral angles of 52° and 122°, suggesting that a more planar orientation is favored rather than a completely orthogonal one (Dong et al., 2021). The lowest energy rotamer, with a dihedral angle of 52°, exhibited energy differences of 1.72 eV and 1.74 eV compared to the higher energy rotamers at 2° and 172°, respectively.

**FIGURE 6:**
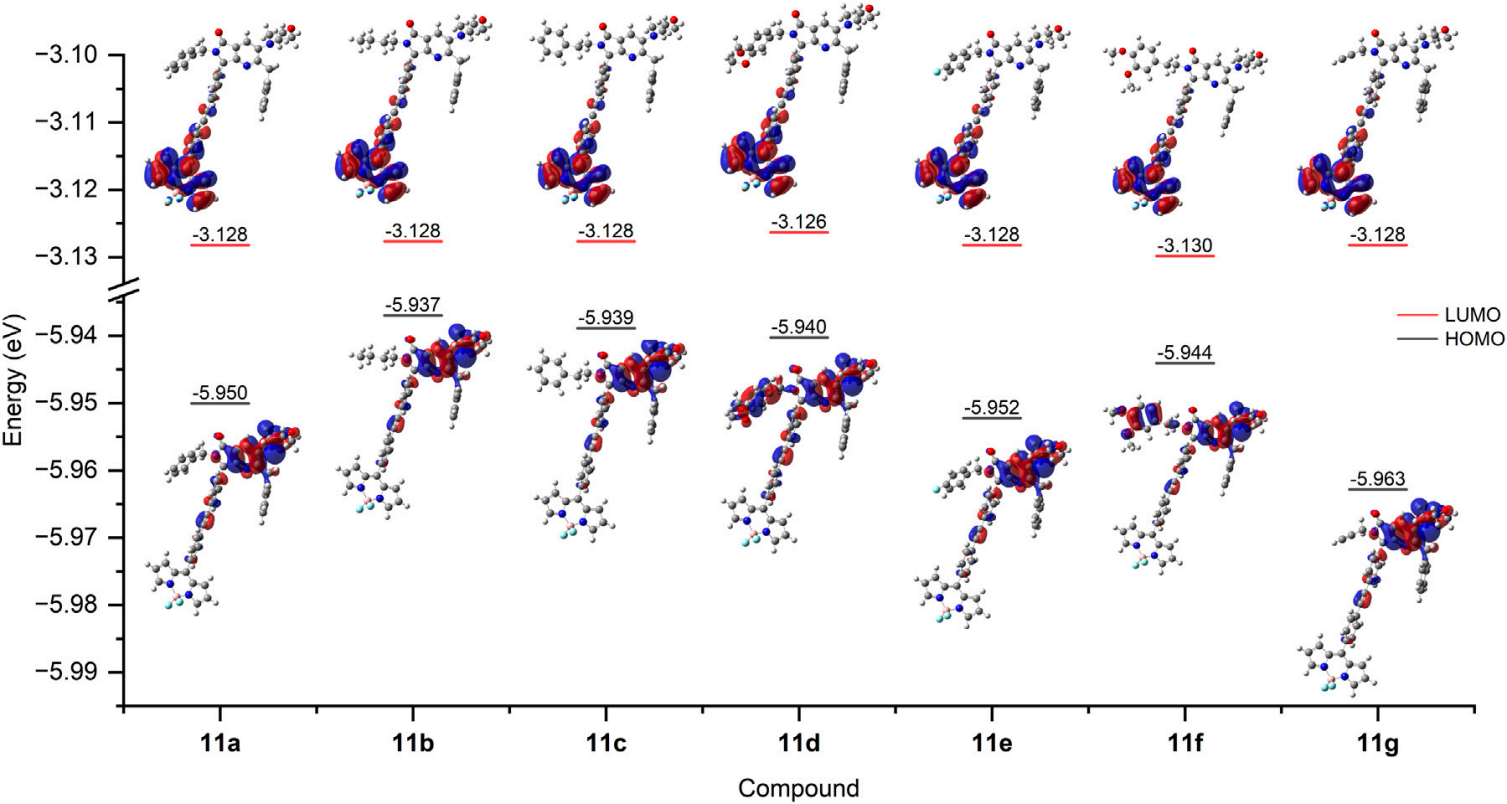
Computed ground state frontier molecular orbitals and energies of the synthesized compounds (B3LYP/6-311G(d,p), DMSO as solvent).

Based on the ground state optimized geometry of compounds **11a-g**, the electronic transitions were determined by time dependent DFT (TD-DFT) using the B3LYP functional and the 6-311+G(d,p) basis set with DMSO as solvent. Table 5 shows the experimental and theoretical absorption maxima for the BODIPY and pyrrolo[3,4-*b*]pyridin-5-one fragments, oscillator strengths and the orbitals involved in the transitions with the corresponding contributions. Analysis of the frontier molecular orbitals revealed that for compounds **11a**, **11b**, **11c**, **11e** and **11g**, the highest occupied molecular orbital (HOMO) distribution is located mainly in the pyrrolo[3,4-*b*]pyridin-5-one core, including the morpholine fragment. For compounds **11d** and **11f**, the HOMO is also present in the electron rich 3,4-dimethoxybenzyl and 3,4-dimethoxyphenethyl groups, respectively. None of the compounds showed electron density contribution from the benzyl substituent. Regarding the lowest unoccupied molecular orbital (LUMO), it is distributed in the *meso*-phenyl BODIPY system on all compounds and no overlap with the HOMO can be seen. The LUMO energies are very similar among the compounds differing by only ±0.002 eV, however, the HOMO energies show a higher variation, attributed to the different substituents of the pyrrolo[3,4-*b*]pyridin-5-one (Figure 6).

**TABLE 5:** Selected experimental UV-Vis and TD-DFT calculations of the absorption maxima of 11a-g in DMSO.

	λ_max abs_ (nm)			
Compound	Experimental	Theoretical	Oscillator strength (*f*)	Main contributions
11a	506	485	0.359	HOMO-1→LUMO (40%) HOMO→LUMO (60%)
	285	283	0.106	HOMO→LUMO+3 (97%)
11b	505	485	0.327	HOMO-2→LUMO (15%) HOMO-1→LUMO (21%) HOMO→LUMO (64%)
	285	282	0.108	HOMO→LUMO+3 (94%)
11c	505	485	0.327	HOMO-2→LUMO (36%) HOMO→LUMO (64%)
	285	283	0.115	HOMO→LUMO+3 (89%)
11d	505	489	0.326	HOMO-3→LUMO (27%) HOMO-1→LUMO (10%) HOMO→LUMO (63%)
	285	283	0.101	HOMO→LUMO+3 (94%)
11e	510	485	0.361	HOMO-2→LUMO (7%) HOMO-1→LUMO (34%) HOMO→LUMO (59%)
	290	283	0.104	HOMO→LUMO+3 (95%)
11f	506	484	0.335	HOMO-3→LUMO (36%) HOMO→LUMO (64%)
	286	282	0.112	HOMO→LUMO+3 (90%)
11g	506	482	0.358	HOMO-2→LUMO (42%) HOMO→LUMO (58%)
	286	283	0.108	HOMO→LUMO+3 (100%)

In all cases, the lowest energy absorption band, attributed to the BODIPY fragment, consists predominantly of a HOMO→LUMO transition (≥58% contribution for all compounds) with considerable oscillator strength, and was computed with a hypsochromic shift between 16 and 25 nm compared to the experimental value. The HOMO is located within the pyrrolo[3,4-*b*]pyridin-5-one, while the LUMO is in the *meso*-phenyl BODIPY system, making this transition of charge-transfer character. Other transitions contributing to this absorption band are of the type of HOMO-x→LUMO, with x = 1, 2, 3. These higher HOMOs are located within the BODIPY fragment or in the phenyl-alkyne-phenyl bridge. On the other hand, the highest energy absorption band, which is attributed to the pyrrolo[3,4-*b*]pyridin-5-one, was reproduced with high precision as it only differed by 2–7 nm from the experimental results. For all compounds in general, the most contributing electronic transition consisted of a HOMO→LUMO+3 transition (≥89% contribution for all compounds). Both molecular orbitals are located within the pyrrolo[3,4-*b*]pyridin-5-one fragment.

Additional TD-DFT calculations were performed in CHCl_3_ and PhMe to assess the influence of different solvents in the frontier molecular orbitals (Supplementary Material). Computed absorption maxima in CHCl_3_ and PhMe are slightly blue shifted in comparison with DMSO, which was expected since DMSO is the more polar solvent. Oscillator strengths are higher in PhMe compared to DMSO and CHCl_3_, indicating that in this solvent the compounds may show a more intense absorption and emission. The LUMO energies in CHCl_3_ and PhMe showed a considerable increase with respect to DMSO, resulting in a larger HOMO-LUMO gap. This may be attributed to the capability of DMSO to form hydrogen bonds, thus lowering the energy of the LUMO. Another noticeable difference is in the distribution of the HOMO since in CHCl_3_ and PhMe it shows a greater localization in the phenyl-alkyne-phenyl system than in DMSO (Supplementary Tables S5, S6).

## Conclusion

A series of seven new *bis*-chromophoric *meso*-phenyl BODIPY-pyrrolo[3,4-*b*]pyridin-5-one conjugates was synthesized in 20%-46% yields by coupling the Ugi-Zhu reaction to a cascade process initiated by maleic anhydride. Using scandium(III) triflate at 10 mol% catalyst loading, microwave heating and PhMe as solvent produced the best yields. The UV-Vis spectra of the compounds showed two main absorption bands at approximately 285 and 506 nm, corresponding to pyrrolo[3,4-*b*]pyridin-5-one and *meso*-phenyl-BODIPY, respectively. The fluorescence response to viscosity was investigated finding that compound **11g** had the best linear response within the seven studied compounds. Theoretical calculations were used to determine the molecular orbitals involved in the absorption. For all compounds the lowest energy absorption was composed mainly of a HOMO-LUMO transition, with the HOMO located in the pyrrolo[3,4-*b*]pyridin-5-one, and the LUMO on the BODIPY fragment. This multicomponent approach enabled us to synthesize bis-chromophoric compounds exhibiting a linear fluorescence response to viscosity variations, which could be of use when these types of sensors are desired.

## Materials and methods

### General considerations

^1^H and ^13^C Nuclear Magnetic Resonance (NMR) spectra were acquired on a Bruker AMX Advance III spectrometer (500 MHz, Fällande, Uster, Switzerland). The solvent used for NMR experiments was deuterated chloroform (CDCl_3_). Chemical shifts are reported in parts per million (δ/ppm). Coupling constants are reported in Hertz (J/Hz). Internal reference for NMR spectra was tetramethylsilane (TMS) at 0.00 ppm. Multiplicities of the signals are reported using the standard abbreviations: singlet (s), doublet (d), triplet (t), quartet (q), and multiplet (m). NMR spectra were analysed using the MestReNova software (Ver. 12.0.0-20080). HRMS spectra were acquired by electrospray ionization (ESI) on a Micro-TOF II spectrometer Bruker Daltonics GmbH (Bremen, Germany). HRMS samples were injected directly (Apollo source) and analysed by the Time-of-Flight method (TOF). HRMS spectra were analysed using the Compass analysis software (Ver. 1.5, Flex Control and Flex Analysis by Bruker Daltonics, Inc.). Microwave-assisted reactions were performed in closed-vessel mode on a CEM Discover SP MW-reactor (Matthews, North Carolina, CA, United States). Reaction progress was monitored by thin-layer chromatography (TLC) and the spots were visualized under ultraviolet (UV) light (254 or 365 nm). Flash columns packed with silica gel in a 0.063–0.200 mm mesh particle size were used to purify the products. Mixtures of hexanes (Hex) and ethyl acetate (EtOAc) in 2:3 or 3:2 (*v/v*) proportions were used to run TLC, silica gel columns, and to measure the retention factor (R_*f*_) values (using the same mobile phase for all the experiments). R_*f*_ values were measured after an elution of 4 cm. All starting reagents and solvents were used as received (without further purification, distillation, or dehydration). Chemical structures were drawn using the ChemDraw Professional software (Ver. 15.0.0.106, Perkin Elmer Informatics, Cambridge, MA, United States).

### Synthesis

#### Synthesis of BODIPY-substituted aldehyde 1

This compound was synthesized as previously described in the literature (Xochitiotzi-Flores et al., 2016).

#### Synthesis and characterization of pyrrolo[3,4-*b*]pyridin-5-ones 11a-g

General procedure (GP): The corresponding BODIPY-substituted aldehyde (1.0 equiv.) and amines (1.2 equiv.) were placed in a sealed 10 mL CEM Discover microwave reaction tube and diluted with 1.0 mL of PhMe. Then, the mixture was stirred and heated using microwave irradiation (60°C, 100 W) for 30 min, after which scandium (III) triflate (10 mol%) was added. The mixture was stirred and heated using microwave irradiation (60°C, 100 W) for 5 min, and then the corresponding isocyanide 5 (1.2 equiv.) was added. The resulting mixture was stirred and heated using microwave irradiation (60°C, 100 W) for 30 min, and then maleic anhydride (7) (1.4 equiv.) was added. Finally, the reaction mixture was stirred and heated using microwave irradiation (60°C, 100 W) for 30 min. The crude of the reaction was purified by column chromatography using a mixture of Hex and EtOAc in a 2:3 (*v/v*) proportion. The isolated compound was further purified by preparative TLC using a mixture of Hex and EtOAc as mobile phase in a 2:3 (*v/v*) or 1:4 (*v/v*) proportions.

#### Synthesis of 2,6-dibenzyl-7-(4-((4-(5,5-difluoro-5H-4λ^4^,5λ^4^-dipyrrolo[1,2-*c*:2′,1′-*f*][1,3,2]diazaborinin-10-yl)phenyl)ethynyl)phenyl)-3-morpholino-6,7-dihydro-5*H*-pyrrolo[3,4-*b*]pyridin-5-one (11a)

According to the GP, *meso*-(5-((4-formylphenyl)ethynyl)phenyl)-4,4-difluoro-4-bora-3a,4a-diaza-*s*-indacene (38.0 mg), benzylamine (14.2 μL), scandium(III) triflate (4.4 mg), 2-isocyano-1-morpholino-3-phenylpropan-1-one (33.2 mg) and maleic anhydride (12.3 mg). Compound **11a** (33.8 mg, 46%) was isolated as an amorphous dark red thick oil. R_*f*_ = 0.20 (EtOAc-Hex 2:3 *v/v*); ^1^H-NMR (500 MHz, CDCl_3_): δ 7.96 (s, 2H, H-37, H-38), 7.92 (s, 1H, H-6), 7.69 (d, *J* = 8.6 Hz, 2H, H-24, H-28), 7.58 (dd, J = 8.6, 2.1 Hz, 4H, H-17, H-19, H-25, H-27), 7.33–7.29 (m, 2H, -H-50, H-52), 7.22–7.08 (m, 10H, H-16, H-20, H-49, H-51, H-53, H-54, H-55, H-56, H-57, H-58), 6.95 (d, *J* = 4.1 Hz, 2H, H-35, H-40), 6.57 (dd, *J* = 4.3, 1.2 Hz, 2H, H-36, H-39), 5.46 (d, *J* = 14.9 Hz, 1H, H-14), 5.27 (s, 1H, H-9), 4.29 (d, *J* = 14.0 Hz, 1H, H-12), 4.17 (d, *J* = 14.0 Hz, 1H, H-12′) 3.84–3.77 (m, 5H, H-14′, H-44, H-46), 2.88–2.77 (m, 4H, H-43, H-47). ^13^C-NMR (126 MHz, CDCl_3_): δ 167.0 (C-7), 162.2 (C-4), 160.1 (C-2), 148.0 (C-5), 146.3 (C-29), 144.4 (C-37, C-38), 139.2 (C-13), 136.6 (C-48), 136.2 (C-15), 134.7 (C-30, C-34), 133.7 (C-23), 132.3 (C-25, C-27), 131.6 (C-24, C-28), 131.4 (C-35, C-40), 130.6 (C-17, C-19), 128.8 (C-49, C-53), 128.8 (C-54, C-58), 128.5 (C-50, C-52), 128.2 (C-55, C-57), 127.8 (C-51), 126.2 (C-56), 125.9 (C-18), 124.0 (C-6), 123.8 (C-1), 123.1 (C-26), 118.7 (C-36, C-39), 91.7 (C-21), 89.1 (C-22), 67.1 (C-44, C-46), 64.1 (C-9), 53.1 (C-43, C-47), 44.0 (C-14), 40.0 (C-12). HRMS: (ESI^+^) *m/z* calcd. for [M−H]^+^ C_48_H_39_BF_2_N_5_O_2_^+^ 766.3159 found 766.3159 (error = 1.2 ppm).

#### Synthesis of 2-benzyl-6-butyl-7-(4-((4-(5,5-difluoro-5*H*-4λ^4^,5λ^4^-dipyrrolo[1,2-*c*:2′,1′-*f*][1,3,2]diazaborinin-10-yl)phenyl)ethynyl)phenyl)-3-morpholino-6,7-dihydro-5*H*-pyrrolo[3,4-*b*]pyridin-5-one (11b)

According to the GP, *meso*-(5-((4-formylphenyl)ethynyl)phenyl)-4,4-difluoro-4-bora-3a,4a-diaza-*s*-indacene (59.4 mg), butylamine (17.8 μL), scandium(III) triflate (7.4 mg), 2-isocyano-1-morpholino-3-phenylpropan-1-one (44.0 mg) and maleic anhydride (20.6 mg). Compound **11b** (33.0 mg, 30%) was isolated as an amorphous dark red thick oil. R_*f*_ = 0.425 (EtOAc-Hex 2:3 *v/v*); ^1^H-NMR (500 MHz, CDCl_3_): δ 7.95 (s, 2H, H-50, H-51), 7.88 (s, 1H, H-6), 7.68 (d, *J* = 8.6 Hz, 2H, H-32, H-36), 7.57 (dd, *J* = 8.5, 1.9 Hz, 4H, H-20, H-22, H-33, H-35), 7.24–7.11 (m, 8H, H-19, H-23, H-24, H-25, H-26, H-27, H-28), 6.94 (d, *J* = 4.3 Hz, 2H, H-48, H-53), 6.56 (ddd, *J* = 4.3, 1.9, 0.8 Hz, 2H, H-49, H-52), 5.49 (s, 1H, H-9), 4.31 (d, *J* = 13.9 Hz, 1H, H-12), 4.21 (d, *J* = 13.9 Hz, 1H, H-12′), 4.01 (dt, *J* = 13.9, 7.9 Hz, 1H, H-14), 3.80 (t, *J* = 4.6 Hz, 4H, H- 44, H-46), 2.91 (ddd, *J* = 13.8, 7.7, 5.9 Hz, 1H, H-14′), 2.86–2.79 (m, 4H, H-43, H-47), 1.60–1.52 (m, 2H, H-15), 1.38–1.28 (m, 2H, H-16), 0.91 (t, *J* = 7.4 Hz, 3H, H-17). ^13^C-NMR (126 MHz, CDCl_3_): δ 167.0 (C-7), 161.9 (C-4), 160.0 (C-2), 147.9 (C-5), 146.3 (C-34), 144.4 (C-50, C-51), 139.3 (C-13), 136.6 (C-18), 134.7 (C-38, C-42), 133.6 (C-31), 132.3 (C-20, C-22), 131.6 (C-32, C-36), 131.3 (C-48, C-53), 130.6 (C-33, C-35), 128.7 (C-24, C-28), 128.2 (C-19, C-23), 127.9 (C-25, C-27), 126.1 (C-26), 125.9 (C-21), 124.2 (C-1), 123.8 (C-6), 123.0 (C-37), 118.7 (C-49, C-52), 91.6 (C-30), 89.0 (C-29), 67.1 (C-44, C-46), 64.9 (C-9), 53.0 (C-43, C-47), 40.1 (C-12), 40.0 (C-14), 30.3 (C-15), 20.0 (C-16), 13.7 (C-17). HRMS: (ESI^+^) *m/z* calcd. for [M−H]^+^ C_45_H_40_BF_2_N_5_O_2_^+^ 732.3316 found 732.3338 (error = 2.0 ppm).

#### Synthesis of 2-benzyl-7-(4-((4-(5,5-difluoro-5*H*-4λ^4^,5λ^4^-dipyrrolo[1,2-*c*:2′,1′-*f*][1,3,2]diazaborinin-10-yl)phenyl)ethynyl)phenyl)-3-morpholino-6-phenethyl-6,7-dihydro-5*H*-pyrrolo[3,4-*b*]pyridin-5-one (11c)

According to the GP, *meso*-(5-((4-formylphenyl)ethynyl)phenyl)-4,4-difluoro-4-bora-3a,4a-diaza-*s*-indacene (19.8 mg), phenethylamine (10.7 μL), scandium(III) triflate (2.5 mg), 2-isocyano-1-morpholino-3-phenylpropan-1-one (22.4 mg) and maleic anhydride (10.6 mg). Compound **11c** (10.6 mg, 27%) was isolated as an amorphous dark red thick oil. R_*f*_ = 0.425 (EtOAc-Hex 2:3 *v/v*); ^1^H-NMR (500 MHz, CDCl_3_): δ 7.96 (s, 2H, H-49, H-50), 7.89 (s, 1H, H-6), 7.68 (d, *J* = 8.5 Hz, 2H, H-36, H-40), 7.57 (d, *J* = 8.6 Hz, 2H, H-37, H-39), 7.54 (d, *J* = 8.5 Hz, 2H, H-24, H-26), 7.36–7.21 (m, 5H, H-28, H-29, H-30, H-31, H-32), 7.19–7.11 (m, 7H, H-18, H-19, H-20, H-21, H-22), 7.07 (d, *J* = 7.9 Hz, 2H, H-23, H-27), 6.94 (d, *J* = 4.3 Hz, 2H, H-47, H-52), 6.56 (dt, *J* = 3.5, 1.1 Hz, 2H, H-48, H-51), 5.20 (s, 1H, H-9), 4.34–4.15 (m, 2H, H-11, H-15), 3.80 (dd, *J* = 5.7, 3.5 Hz, 4H, H-56, H-58), 3.14 (dt, *J* = 13.9, 7.5 Hz, 1H, H-11′), 2.97 (dt, *J* = 13.5, 7.7 Hz, 1H, 12), 2.92–2.77 (m, 5H, H-12′, H-55, H-59). ^13^C-NMR (126 MHz, CDCl_3_): δ 167.0 (C-7), 161.9 (C-4), 159.9 (C-2), 147.9 (C-5), 146.3 (C-41), 144.3 (C-49, C-50), 139.1 (C-16), 138.6 (C-13), 136.3 (C-14), 134.7 (C-42, C-46), 133.6 (C-35), 132.2 (C-24, C-26), 131.6 (C-36, C-40), 131.3 (C-47, C-52), 130.5 (C-37, C-39), 128.7 (C-18, C-19, C-21, C-22, C-28, C-29, C-31, C-32), 128.1 (C-23), 128.0 (C-27), 126.6 (C-20), 126.1 (C-30), 125.9 (C-38), 124.0 (C-1), 123.8 (C-6), 123.1 (C-25), 118.7 (C-48, C-51), 91.6 (C-33), 89.0 (C-34), 67.1 (C-56, C-58), 65.6 (C-9), 53.0 (C-55, C-59), 42.0 (C-11), 40.0 (C-15), 34.7 (C-12). HRMS: (ESI^+^) *m/z* calcd. for [M−H]^+^ C_49_H_41_BF_2_N_5_O_2_^+^ 780.3316 found 780.3310 (error = 1.8 ppm).

#### Synthesis of 2-benzyl-7-(4-((4-(5,5-difluoro-5*H*-4λ^4^,5λ^4^-dipyrrolo[1,2-*c*:2′,1′-*f*][1,3,2]diazaborinin-10-yl)phenyl)ethynyl)phenyl)-6-(3,4-dimethoxybenzyl)-3-morpholino-6,7-dihydro-5*H*-pyrrolo[3,4-*b*]pyridin-5-one (11d)

According to the GP, *meso*-(5-((4-formylphenyl)ethynyl)phenyl)-4,4-difluoro-4-bora-3a,4a-diaza-*s*-indacene (32.5 mg), 3,4-dimethoxybenzyl amine (14.8 μL), scandium(III) triflate (4.1 mg), 2-isocyano-1-morpholino-3-phenylpropan-1-one (24.0 mg) and maleic anhydride (11.6 mg). Compound **11d** (10.6 mg, 27%) was isolated as an amorphous dark red thick oil. R_*f*_ = 0.25 (EtOAc-Hex 3:2 *v/v*); ^1^H-NMR (500 MHz, CDCl_3_): δ 7.96 (s, 2H, H-53, H-54), 7.91 (s, 1H, H-6), 7.69 (d, *J* = 8.5 Hz, 2H, H-40, H-44), 7.58 (d, *J* = 8.6 Hz, 4H, H-23, H-25, H-41, H-43), 7.22–7.09 (m, 8H, H-22, H-26, H-27, H-28, H-29, H-30, H-31), 6.95 (d, *J* = 4.3 Hz, 2H, H-51, H-56), 6.79 (d, *J* = 8.1 Hz, 1H, H-18), 6.75 (d, *J* = 2.0 Hz, 1H, H-21), 6.70 (dd, *J* = 8.1, 2.0 Hz, 1H, H-17), 6.57 (ddd, *J* = 4.3, 2.0, 0.8 Hz, 2H, H-52, H-55), 5.38 (d, *J* = 14.7 Hz, 1H, H-11), 5.28 (s, 1H, H-9), 4.29 (d, *J* = 13.9 Hz, 1H, H-14), 4.16 (d, *J* = 13.9 Hz, 1H, H-14′), 3.87 (s, 3H, H-59), 3.84–3.79 (m, 7H, H-34, H-36, H-60), 3.75 (d, *J* = 14.7 Hz, 1H, H-11′), 2.83 (ddt, *J* = 16.3, 11.5, 5.9 Hz, 4H, H-33, H-37). ^13^C-NMR (126 MHz, CDCl_3_): δ 167.0 (C-7), 162.1 (C-4), 160.0 (C-2), 149.3 (C-19), 148.8 (C-20), 147.9 (C-5), 146.3 (C-45), 144.4 (C-53, C-54), 139.1 (C-15), 136.3 (C-13), 134.7 (C-46, C-50), 133.7 (C-39), 132.3 (C-23, C-25), 131.6 (C-40, C-44), 131.3 (C-51, C-56), 130.6 (C-41, C-43), 129.1 (C-12), 128.8 (C-22, C-26), 128.2 (C-27, C-28, C-30, C-31), 126.2 (C-29), 125.9 (C-24), 124.0 (C-6), 123.8 (C-1), 123.1 (C-42), 121.0 (C-17), 118.7 (C-52, C-55), 111.9 (C-21), 111.2 (C-18), 91.6 (C-38), 89.1 (C-32), 67.1 (C-34, C-36), 64.1 (C-9), 55.9 (C-59, C-60), 53.0 (C-33, C-37), 43.9 (C-11), 40.0 (C-14). HRMS: (ESI^+^) *m/z* calcd. for [M−H]^+^ C_50_H_43_BF_2_N_5_O_4_^+^ 826.3371 found 826.3375 (error = 0.5 ppm).

#### Synthesis of 2-benzyl-7-(4-((4-(5,5-difluoro-5*H*-4λ^4^,5λ^4^-dipyrrolo[1,2-*c*:2′,1′-*f*][1,3,2]diazaborinin-10-yl)phenyl)ethynyl)phenyl)-6-(4-fluorobenzyl)-3-morpholino-6,7-dihydro-5*H*-pyrrolo[3,4-*b*]pyridin-5-one (11e)

According to the GP, *meso*-(5-((4-formylphenyl)ethynyl)phenyl)-4,4-difluoro-4-bora-3a,4a-diaza-*s*-indacene (19.8 mg), 4-fluorobenzylamine (6.8 μL), scandium(III) triflate (2.5 mg), 2-isocyano-1-morpholino-3-phenylpropan-1-one (14.8 mg) and maleic anhydride (10.6 mg). Compound **11e** (15.2 mg, 39%) was isolated as an amorphous dark red thick oil. R_*f*_ = 0.3 (EtOAc-Hex 2:3 *v/v*); ^1^H-NMR (500 MHz, CDCl_3_): δ 7.96 (s, 2H, H-53, H-54), 7.91 (s, 1H, H-6), 7.69 (d, *J* = 8.5 Hz, 2H, H-40, H-44), 7.58 (dd, *J* = 8.5, 2.7 Hz, 4H, H-23, H-25, H-41, H-43), 7.21–7.10 (m, 9H, H-17, H-21, H-22, H-26, H-27, H-28, H-29, H-30, H-31), 7.00 (t, *J* = 8.6 Hz, 2H, H-18, H-20), 6.95 (d, *J* = 4.2 Hz, 2H, H-51, H-56), 6.57 (dd, *J* = 4.4, 1.1 Hz, 2H, H-52, H-55), 5.37 (d, *J* = 14.9 Hz, 1H, H-11), 5.26 (s, 1H, H-9), 4.29 (d, *J* = 13.9 Hz, 1H, H-15), 4.17 (d, *J* = 13.9 Hz, 1H, H-15′), 3.84–3.77 (m, 5H, H-11′, H-33, H-35), 2.89–2.78 (m, 4H, H-32, H-36). ^13^C-NMR (126 MHz, CDCl_3_): δ 167.0 (C-7), 163.3 (^1^ *J*_*C-F*_ = 246.4 Hz, C-19), 162.3 (C-4), 159.9 (C-2), 148.0 (C-5), 146.3 (C-45), 144.4 (C-53, C-54), 139.1 (C-16), 136.1 (C-13), 134.7 (C-46, C-50), 133.7 (C-39), 132.4 (^4^ *J*_*C-F*_ = 2.8 Hz, C-12), 132.4 (C-41, C-43), 131.6 (C-40, C-44), 131.3 (C-52, C-55), 130.6 (C-23, C-25), 130.2 (^3^ *J*_*C-F*_ = 8.2 Hz, C-17, C-21), 128.8 (C-22, C-26), 128.2 (C-27, C-28), 128.1 (C-30, C-31), 126.2 (C-29), 125.9 (C-42), 124.0 (C-6), 123.7 (C-1), 123.2 (C-24), 118.7 (C-52, C-55), 115.7 (^2^ *J*_*C-F*_ = 21.5 Hz, C-18, C-20), 91.6 (C-37), 89.1 (C-38), 67.1 (C-33, C-35), 64.2 (C-9), 53.0 (C-32, C-36), 43.3 (C-11), 40.0 (C-15). HRMS: (ESI^+^) *m/z* calcd. for [M−H]^+^ C_48_H_38_BF_3_N_5_O_2_^+^ 784.3065 found 784.3072 (error = 0.2 ppm).

#### Synthesis of 2-benzyl-7-(4-((4-(5,5-difluoro-5*H*-4λ^4^,5λ^4^-dipyrrolo[1,2-*c*:2′,1′-*f*][1,3,2]diazaborinin-10-yl)phenyl)ethynyl)phenyl)-6-(3,4-dimethoxyphenethyl)-3-morpholino-6,7-dihydro-5*H*-pyrrolo[3,4-*b*]pyridin-5-one (11f)

According to the GP, *meso*-(5-((4-formylphenyl)ethynyl)phenyl)-4,4-difluoro-4-bora-3a,4a-diaza-*s*-indacene (59.4 mg), 3,4-dimethoxyphenethylamine (30.4 μL), scandium(III) triflate (7.4 mg), 2-isocyano-1-morpholino-3-phenylpropan-1-one (44.5 mg) and maleic anhydride (27.5 mg). Compound **11f** (25.3 mg, 20%) was isolated as an amorphous dark red thick oil. R_*f*_ = 0.2 (EtOAc-Hex 3:2 *v/v*); ^1^H-NMR (600 MHz, CDCl_3_): δ 7.96 (s, 2H, H-56, H-57), 7.88 (s, 1H, H-6), 7.68 (d, *J* = 8.5 Hz, 2H, H-38, H-42), 7.58 (d, *J* = 8.6 Hz, 2H, H-39, H-41), 7.55 (d, *J* = 8.4 Hz, 2H, H-26, H-28), 7.21–7.11 (m, 5H, H-30, H-31, H-32, H-33, H-34), 7.09 (d, *J* = 8.0 Hz, 2H, H-25, H-29), 6.94 (d, *J* = 4.3 Hz, 2H, H-54, H-59), 6.78 (d, *J* = 8.1 Hz, 1H, H-18), 6.70 (dd, *J* = 8.1, 2.0 Hz, 1H, H-19), 6.66 (d, *J* = 2.0 Hz, 1H, H-15), 6.57 (dd, *J* = 4.3, 1.2 Hz, 2H, H-55, H-58), 5.19 (s, 1H, H-9), 4.33–4.17 (m, 3H, H-12, H-23), 3.86 (s, 3H, H-20), 3.83–3.78 (m, 7H, H-21, H-50, H-52), 3.13 (dt, *J* = 14.4, 7.4 Hz, 1H, H-12′), 2.96–2.90 (m, 1H, H-13), 2.86–2.78 (m, 5H, H-13′, H-49, H-53). ^13^C NMR (151 MHz, CDCl_3_): δ 167.1 (C-7), 162.0 (C-4), 160.0 (C-2), 149.0 (C-16), 147.9 (C-5), 147.8 (C-17), 146.3 (C-43), 144.4 (C-56, C-57), 139.1 (C-24), 136.3 (C-11), 134.7 (C-44, C-48), 133.6 (C-37), 132.2 (C-26, C-28), 131.6 (C-38, C-42), 131.3 (C-54, C-59), 131.0 (C-14) 130.6 (C-39, C-41), 128.7 (C-30, C-34), 128.2 (C-25, C-29), 128.1 (C-31, C-33), 126.2 (C-32), 125.9 (C-27), 124.0 (C-6), 123.8 (C-1), 123.1 (C-40), 120.7 (C-18), 118.7 (C-55, C-58), 111.7 (C-15), 111.3 (C-19), 91.5 (C-35), 89.1 (C-36), 67.1 (C-50, C-52), 65.6 (C-9), 55.9 (C-20), 55.8 (C-21), 53.0 (C-49, C-53), 41.9 (C-12), 40.1 (C-23), 34.2 (C-13). HRMS: (ESI^+^) *m/z* calcd. for [M−H]^+^ C_50_H_43_BF_2_N_5_O_4_^+^ 839.3449 found 839.3479 (error = 2.6 ppm).

#### Synthesis of 2-benzyl-7-(4-((4-(5,5-difluoro-5*H*-4λ^4^,5λ^4^-dipyrrolo[1,2-*c*:2′,1′-*f*][1,3,2]diazaborinin-10-yl)phenyl)ethynyl)phenyl)-3-morpholino-6-(prop-2-yn-1-yl)-6,7-dihydro-5*H*-pyrrolo[3,4-*b*]pyridin-5-one (11g)

According to the GP, *meso*-(5-((4-formylphenyl)ethynyl)phenyl)-4,4-difluoro-4-bora-3a,4a-diaza-*s*-indacene (59.5 mg), propargylamine (11.5 μL), scandium(III) triflate (7.4 mg), 2-isocyano-1-morpholino-3-phenylpropan-1-one (43.9 mg) and maleic anhydride (12.0 mg). Compound **11g** (33.3 mg, 31%) was isolated as an amorphous dark red thick oil. R_*f*_ = 0.525 (EtOAc-Hex 3:2 *v/v*); ^1^H-NMR (500 MHz, CDCl_3_): δ 7.96 (s, 2H, H-49, H-50), 7.90 (s, 1H, H-6), 7.68 (d, *J* = 8.5 Hz, 2H, H-26, H-30), 7.58 (dd, *J* = 8.5, 1.8 Hz, 4H, H-19, H-21, H-27, H-29), 7.24 (d, *J* = 8.0 Hz, 2H, H-18, H-22), 7.22–7.12 (m, 5H, H-31, H-32, H-33, H-34, H-35), 6.94 (d, *J* = 4.3 Hz, 2H, H-47, H-52), 6.56 (ddd, *J* = 4.3, 2.0, 0.8 Hz, 2H, H-48, H-51), 5.71 (s, 1H, H-9), 4.96 (dd, *J* = 17.7, 2.6 Hz, 1H, H-11), 4.32 (d, *J* = 13.9 Hz, 1H, H-15), 4.23 (d, *J* = 13.9 Hz, 1H, H-15′), 3.81 (t, *J* = 4.6 Hz, 4H, H-37, H-39), 3.58 (dd, *J* = 17.7, 2.5 Hz, 1H, H-11′), 2.89–2.78 (m, 4H, H-36, H-40), 2.27 (t, *J* = 2.5 Hz, 1H, H-17). NMR (151 MHz, CDCl_3_): δ 166.4 (C-7), 162.5 (C-4), 159.8 (C-2), 148.1 (C-5), 146.3 (C-41), 144.4 (C-49, C-50), 139.1 (C-16), 135.8 (C-13), 134.7 (C-42, C-46), 133.6 (C-25), 132.3 (C-19, C-21), 131.6 (C-26, C-30), 131.3 (C-47, C-52), 130.6 (C-27, C-29), 128.8 (C-31, C-32, C-34, C-35), 128.2 (C-18, C-22), 126.2 (C-33), 125.9 (C-28), 124.0 (C-6), 123.5 (C-1), 123.3 (C-20), 118.7 (C-48, C-41), 91.6 (C-24), 89.1 (C-23), 77.8 (C-12), 72.7 (C-17), 67.1 (C-37, C-39), 64.2 (C-9), 53.0 (C-36, C-40), 40.1 (C-15), 29.8 (C-11). HRMS: (ESI^+^) *m/z* calcd. for [M−H]^+^ C_44_H_35_BF_2_N_5_O_2_^+^ 714.2846 found 714.2855 (error = 0.2 ppm).

## Data Availability

The original contributions presented in the study are included in the article/Supplementary Material, further inquiries can be directed to the corresponding authors.

## References

[B1] Boens N. Leen V. Dehaen W. (2012). Fluorescent indicators based on BODIPY. Chem. Soc. Rev. 41, 1130–1172. 10.1039/C1CS15132K 21796324

[B2] Cao X. Lin W. Zheng K. He L. (2012). A near-infrared fluorescent turn-on probe for fluorescence imaging of hydrogen sulfide in living cells based on thiolysis of dinitrophenyl ether. Chem. Commun. 48, 10529–10531. 10.1039/C2CC34031C 22992474

[B3] Dong Y. Taddei M. Doria S. Bussotti L. Zhao J. Mazzone G. (2021). Torsion-induced nonradiative relaxation of the singlet excited state of *meso*-thienyl Bodipy and charge separation, charge recombination-induced intersystem crossing in its compact electron donor/acceptor dyads. J. Phys. Chem. B 125, 4779–4793. 10.1021/acs.jpcb.1c00053 33929843

[B4] Fayol A. Housseman C. Sun X. Janvier P. Bienaymé H. Zhu J. (2005). Synthesis of α-isocyano-α-alkyl(aryl)acetamides and their use in the multicomponent synthesis of 5-aminooxazole, pyrrolo[3,4-*b*]pyridin-5-one and 4,5,6,7-tetrahydrofuro[2,3-*c*]pyridine. Synthesis 1, 0161–0165. 10.1055/s-2004-831225

[B5] Flores-Reyes J. C. Islas-Jácome A. González-Zamora E. (2021). The Ugi three-component reaction and its variants. Org. Chem. Front. 8, 5460–5515. 10.1039/D1QO00313E

[B6] Flores-Reyes J. C. Rojas-Montoya S. M. Blancarte-Carrazco L. Xochitiotzi-Flores E. Guarin C. A. Farfán N. (2024). Multicomponent synthesis and photophysical properties of *meso*-thienyl BODIPY-pyrrolo[3,4-*b*]pyridin-5-ones. An experimental and theoretical study. J. Lumin. 273, 120698. 10.1016/j.jlumin.2024.120698

[B7] Förster T. Hoffmann G. (1971). Die Viskositätsabhängigkeit der Fluoreszenzquantenausbeuten einiger Farbstoffsysteme. Phys. Chem. 75, 63–76. 10.1524/zpch.1971.75.1_2.063

[B8] Frisch M. J. Trucks G. W. Schlegel H. B. Scuseria G. E. Robb M. A. Cheeseman J. R. (2010). Gaussian 09, revision B.01. Wallingford CT: Gaussian, Inc.

[B9] Grotkopp O. Mayer B. Müller T. J. J. (2018). Diversity-oriented synthesis and optical properties of bichromophoric pyrrole-fluorophore conjugates. Front. Chem. 6, 579. 10.3389/fchem.2018.00579 30542648 PMC6277781

[B10] Ibarra I. Islas-Jácome A. González-Zamora E. (2018). Synthesis of polyheterocycles via multicomponent reactions. Org. Biomol. Chem. 16, 1402–1418. 10.1039/c7ob02305g 29238790

[B11] Janvier P. Sun X. Bienaymé H. Zhu J. (2002). Ammonium chloride-promoted four-component synthesis of pyrrolo[3,4-*b*]pyridin-5-one. J. Am. Chem. Soc. 11, 2560–2567. 10.1021/ja017563a 11890807

[B12] Kashyap K. S. Kumar A. Hira S. K. Dey S. (2019). Recognition of Al^3+^ through the off-on mechanism as a proficient driving force for the hydrolysis of BODIPY conjugated Schiff base and its application in bio-imaging. Inorg. Chim. Acta 498, 119157. 10.1016/j.ica.2019.119157

[B13] Lee H. L. Jang H. J. Lee J. Y. (2020). Single molecule white emission by intra- and inter-molecular charge transfer. J. Mater. Chem. C 8, 10302–10308. 10.1039/D0TC02205E

[B14] Lee S.-C. Heo J. Woo H. C. Lee J.-A. Seo Y. H. Lee C.-L. (2018). Fluorescent molecular rotors for viscosity sensors. Chem. Eur. J. 24, 13706–13718. 10.1002/chem.201801389 29700889

[B15] Levi L. Müller T. J. J. (2016). Multicomponent syntheses of functional chromophores. Chem. Soc. Rev. 45, 2825–2846. 10.1039/C5CS00805K 26898222

[B16] Li F. Yang S. Ciringh Y. Seth J. Martin C. Singh D. (1998). Design, synthesis, and photodynamics of light-harvesting arrays comprised of a porphyrin and one, two, or eight boron-dipyrrin accessory pigments. J. Am. Chem. Soc. 120, 10001–10017. 10.1021/ja9812047

[B17] Lin Q. Gruskos J. J. Buccella D. (2016). Bright, red emitting fluorescent sensor for intracellular imaging of Mg^2+^ . Org. Biomol. Chem. 14, 11381–11388. 10.1039/C6OB02177H 27858038

[B18] Longo Jr. Siquiera F. Anjos N. Santos G. (2021). Scandium(III)-triflate-catalyzed multicomponent reactions for the synthesis of nitrogen heterocycles. ChemistrySelect, 6, 5097–5109. 10.1002/slct.202101032

[B19] Loudet A. Burgess K. (2007). BODIPY dyes and their derivatives: syntheses and spectroscopic properties. Chem. Rev. 107, 4891–4932. 10.1021/cr078381n 17924696

[B20] Ma D. Zhao G. Chen H. Zhou R. Zhang G. Tian W. (2022). Creation of BODIPYs-based red OLEDs with high color purity via modulating the energy gap and restricting rotation of substituents. Dyes Pigm 203, 110377. 10.1016/j.dyepig.2022.110377

[B21] Miao W. Yu C. Hao E. Jiao L. (2019). Functionalized BODIPYs as fluorescent molecular rotors for viscosity detection. Front. Chem. 7, 825. 10.3389/fchem.2019.00825 31850314 PMC6901978

[B22] Murkherjee S. Thilagar P. (2014). Fine-tuning dual emission and aggregation-induced emission switching in NPI–BODIPY Dyads. Chem. Eur. J. 20, 9052–9062. 10.1002/chem.201305049 24895089

[B23] Nakamura T. Sasabe H. Abe S. Kumada K. Sugiyama R. Hanayama T. (2023). Highly efficient and stable green fluorescent OLEDs with high color purity using a BODIPY derivative. Mol. Syst. Des. Eng. 8, 866–873. 10.1039/D3ME00029J

[B24] Nootem J. Sattayanon C. Daengngern R. Kamkaew A. Wattanathana W. Wannapaiboon S. (2021). BODIPY-pyridylhydrazone probe for fluorescence turn-on detection of Fe^3+^ and its bioimaging application. Chemosensors 9, 165. 10.3390/chemosensors9070165

[B25] Pharande S. Rentería-Gómez M. Gámez-Montaño R. (2020). Synthesis of polyheterocyclic dimers containing restricted and constrained peptidomimetics via IMCR-based domino/double CuAAC click strategy. Molecules 25, 5246. 10.3390/molecules25225246 33187075 PMC7696539

[B26] Rentería-Gómez M. Islas-Jácome A. Pharande S. Vosburg D. Gámez-Montaño R. (2019). Synthesis of tris-heterocycles via a cascade IMCR/aza Diels-Alder+CuAAC strategy. Front. Chem. 7, 546. 10.3389/fchem.2019.00546 31448260 PMC6691067

[B27] Reyes Flores J. Castruita-De León G. Turlakov G. Arias E. Moggio I. Montemayor S. M. (2021). Dual emission of *meso*-phenyleneethynylene–BODIPY oligomers: synthesis, photophysics, and theoretical optoelectronic study. Chem. Eur. J. 27, 2493–2505. 10.1002/chem.202004481 33119951

[B28] Rocha R. O. Rodrigues M. O. Neto B. A. D. (2020). Review on the Ugi multicomponent reaction mechanism and the use of fluorescent derivatives as functional chromophores. ACS Omega 5 (2), 972–979. 10.1021/acsomega.9b03684 31984252 PMC6977082

[B29] Rojas-Montoya S. M. González-Antonio O. Figureoa C. G. Rodríguez-Romero J. Santillan R. Farfán N. (2024). Chemical and thermal stability of novel phenyl-BODIPY symmetric dimer thin films. J. Mol. Struc. 1308, 138036. 10.1016/j.molstruc.2024.138036

[B30] Rotkiewicz K. Grellmann K. H. Grabowski Z. R. (1973). Reinterpretation of the anomalous fluorescense of p-n,n-dimethylamino-benzonitrile. Chem. Phys. Lett. 19, 315–318. 10.1016/0009-2614(73)80367-7

[B31] Saha T. Kand D. Talukdar P. (2013). A colorimetric and fluorometric BODIPY probe for rapid, selective detection of H_2_S and its application in live cell imaging. Org. Biomol. Chem. 11, 8166–8170. 10.1039/C3OB41884G 24178370

[B32] Segura-Olvera D. García-González A. Morales-Salazar I. Islas-Jácome A. Rojas-Aguirre Y. Ibarra I. (2019). Synthesis of pyrrolo[3,4-*b*]pyridin-5-ones via multicomponent reactions and *in vitro*–*in silico* studies against SiHa, HeLa, and CaSki Human cervical carcinoma cell lines. Molecules 24, 2648. 10.3390/molecules24142648 31336585 PMC6680468

[B33] Shikano M. Morimoto M. Naka S. (2021). Near-infrared organic light-emitting diodes of pure fluorescence emission using small-molecule boron-dipyrromethene derivative. Org. Electron. 99, 106320. 10.1016/j.orgel.2021.106320

[B34] Song X. Zhang D. Zhang Y. Lu Y. Duan L. (2020). Strategically modulating carriers and excitons for efficient and stable ultrapure-green fluorescent OLEDs with a sterically hindered BODIPY dopant. Adv. Opt. Mater. 8, 2000483. 10.1002/adom.202000483

[B35] Su D. Oh J. Lee S.-C. Lim J. M. Sahu S. Yu X. (2014). Dark to light! A new strategy for large Stokes shift dyes: coupling of a dark donor with tunable high quantum yield acceptors. Chem. Sci. 5, 4812–4818. 10.1039/C4SC01821D

[B36] Su D. Teoh C. L. Gao N. Xu Q.-H. Chang Y.-T. (2016). A simple BODIPY-based viscosity probe for imaging of cellular viscosity in live cells. Sensors 16, 1397. 10.3390/s16091397 27589762 PMC5038675

[B37] Vázquez-Vera O. Sánchez-Badillo J. Islas-Jácome A. Rentería-Gómez M. Pharande S. Cortes-García C. (2017). An efficient Ugi-3CR/aza Diels–Alder/Pomeranz–Fritsch protocol towards novel aza-analogues of (±)-nuevamine, (±)-lennoxamine and magallanesine: a diversity oriented synthesis approach. Org. Biomol. Chem. 15, 2363–2369. 10.1039/C6OB02572B 28066847

[B38] Wang C. Chi W. Qiao Q. Tan D. Xu Z. Liu X. (2021). Twisted intramolecular charge transfer (TICT) and twists beyond TICT: from mechanisms to rational designs of bright and sensitive fluorophores. Chem. Soc. Rev. 50, 12656–12678. 10.1039/d1cs00239b 34633008

[B39] Xochitiotzi-Flores E. Islas-Mejía A. García-Ortega H. Romero-Avila M. Mendez-Stivalet J. M. Carreón-Castro M. (2016). On the structure of *meso*-substituted F-BODIPYs and their assembly in molecular crystals: an experimental-theoretical approach. J. Organomet. Chem. 805, 148–157. 10.1016/j.jorganchem.2016.01.021

[B40] Xue X. Fang H. Chen H. Zhang C. Zhu C. Bai Y. (2016). *In vivo* fluorescence imaging for Cu^2+^ in live mice by a new NIR fluorescent sensor. Dyes Pigm 130, 116–121. 10.1016/j.dyepig.2016.03.017

[B41] Zamudio-Medina A. García-González A. Herrera-Carrillo G. Zárate-Zárate D. Benavides-Macías A. Tamariz J. (2018). Synthesis of polyheterocyclic pyrrolo[3,4-*b*]pyridin-5-ones via a one-pot (Ugi-3CR/aza DA/N-acylation/aromatization/S_N_2) process. A suitable alternative towards novel aza-analogues of falipamil. Molecules 23, 763. 10.3390/molecules23040763 29584639 PMC6017480

[B42] Zamudio-Medina A. García-González M. Padilla J. González-Zamora E. (2010). Synthesis of a tetracyclic lactam system of nuevamine by four-component reaction and free radical cyclization. Tetrahedron Lett. 51, 4837–4839. 10.1016/j.tetlet.2010.07.047

[B43] Zhang W. Sheng W. Yu C. Wei Y. Wang H. Hao E. (2017). One-pot synthesis and properties of well-defined butadiynylene-BODIPY oligomers. Chem. Commun. 53, 5318–5321. 10.1039/C7CC02393F 28447091

[B44] Zhu H. Fan J. Li M. Cao J. Wang J. Peng X. (2014). A “distorted-BODIPY”-based fluorescent probe for imaging of cellular viscosity in live cells. Chem. Eur. J. 20, 4691–4696. 10.1002/chem.201304296 24595961

[B45] Zhu S. Zhang J. Janjanam J. Vegesna G. Luo F.-T. Tiwari A. (2013). Highly water-soluble BODIPY-based fluorescent probes for sensitive fluorescent sensing of zinc(II). J. Mater. Chem. B 1, 1722–1728. 10.1039/C3TB00249G 32260703

