# Supplementary material for: Synthesis of BODIPY-pyrrolo[3,4-b]pyridin-5-ones via Ugi-Zhu/cascade reactions and studies of fluorescence response toward viscosity
Source: Front Chem. 2024 Oct 18;12:1488933. doi: 10.3389/fchem.2024.1488933 (PMC11527740; doi:10.3389/fchem.2024.1488933)
Supplement: Supplementary file 1 [file DataSheet1.pdf]

SUPPLEMENTARY MATERIAL

Synthesis of BODIPY-pyrrolo[3,4-*b*]pyridin-5-ones *via* Ugi-Zhu/cascade reactions and studies of fluorescence response toward viscosity

Julio C. Flores-Reyes,[1] Annia Galano,[1] Sandra M. Rojas-Montoya,[2] Luis Blancarte-Carrazco,[2] Elba Xochitiotzi-Flores,[2] Héctor García-Ortega,[2] Norberto Farfán,[2,*] Alejandro Islas-Jácome,[1,*] Eduardo González-Zamora[1,*]

[1] Departamento de Química, Universidad Autónoma Metropolitana-Iztapalapa, Av. Ferrocarril San Rafael Atlixco 186, Col. Leyes de Reforma 1A Sección, Iztapalapa, 09310, Ciudad de México, México.

[2] Departamento de Química Orgánica, Facultad de Química, Universidad Nacional Autónoma de México, Ciudad de México, México.

*** Correspondence**: norberto.farfan@gmail.com (N.F.); aij@xanum.uam.mx (A.I.-J.); egz@xanum.uam.mx (E.G.-Z.)

Table of contents

1 Synthesis 3

2 NMR and HRMS spectra 3

2.1 2,6-dibenzyl-7-(4-((4-(5,5-difluoro-5*H*-4λ^4,5λ^4-dipyrrolo[1,2-*c*:2',1'-*f*][1,3,2]diazaborinin-10-yl)phenyl)ethynyl)phenyl)-3-morpholino-6,7-dihydro-5-pyrrolo[3,4-*b*]pyridin-5-one (**11a**) 3

2.2 2-benzyl-6-butyl-7-(4-((4-(5,5-difluoro-5*H*-4λ^4,5λ^4-dipyrrolo[1,2-*c*:2',1'-*f*][1,3,2]diazaborinin-10-yl)phenyl)ethynyl)phenyl)-3-morpholino-6,7-dihydro-5*H*-pyrrolo[3,4-*b*]pyridin-5-one (**11b**) 7

2.3 2-benzyl-7-(4-((4-(5,5-difluoro-5*H*-4λ^4,5λ^4-dipyrrolo[1,2-*c*:2',1'-*f*][1,3,2]diazaborinin-10-yl)phenyl)ethynyl)phenyl)-3-morpholino-6-phenethyl-6,7-dihydro-5*H*-pyrrolo[3,4-*b*]pyridin-5-one (**11c**) 10

2.4 2-benzyl-7-(4-((4-(5,5-difluoro-5*H*-4λ^4,5λ^4-dipyrrolo[1,2-*c*:2',1'-*f*][1,3,2]diazaborinin-10-yl)phenyl)ethynyl)phenyl)-6-(3,4-dimethoxybenzyl)-3-morpholino-6,7-dihydro-5*H*-pyrrolo[3,4-*b*]pyridin-5-one (**11d**) 13

2.5 2-benzyl-7-(4-((4-(5,5-difluoro-5*H*-4λ^4,5λ^4-dipyrrolo[1,2-*c*:2',1'-*f*][1,3,2]diazaborinin-10-yl)phenyl)ethynyl)phenyl)-6-(4-fluorobenzyl)-3-morpholino-6,7-dihydro-5*H*-pyrrolo[3,4-*b*]pyridin-5-one (**11e**) 16

2.6 2-benzyl-7-(4-((4-(5,5-difluoro-5*H*-4λ4,5λ4-dipyrrolo[1,2-*c*:2',1'-*f*][1,3,2]diazaborinin-10-yl)phenyl)ethynyl)phenyl)-6-(3,4-dimethoxyphenethyl)-3-morpholino-6,7-dihydro-5*H*-pyrrolo[3,4-*b*]pyridin-5-one (**11f**) 19

2.7 2-benzyl-7-(4-((4-(5,5-difluoro-5*H*-4λ4,5λ4-dipyrrolo[1,2-*c*:2',1'-*f*][1,3,2]diazaborinin-10-yl)phenyl)ethynyl)phenyl)-3-morpholino-6-(prop-2-yn-1-yl)-6,7-dihidro-5*H*-pyrrolo[3,4-*b*]pyridin-5-one (**11g**) 22

3 Emission spectra of compounds **11a-g** with different viscosities 25

4 Data of DFT and TD-DFT calculations...... 30

4.1 Optimized xyz coordinates (B3LYP/6-31G(d), SMD, DMSO) and excitation energies and oscillator strengths (B3LYP/6-311+G(d,p)) of compound **11a** 30

4.2 Optimized xyz coordinates (B3LYP/6-31G(d), SMD, DMSO) and excitation energies and oscillator strengths (B3LYP/6-311+G(d,p)) of compound **11b** 33

4.3 Optimized xyz coordinates (B3LYP/6-31G(d), SMD, DMSO) and excitation energies and oscillator strengths (B3LYP/6-311+G(d,p)) of compound **11c**...... 35

4.4 Optimized xyz coordinates (B3LYP/6-31G(d), SMD, DMSO) and excitation energies and oscillator strengths (B3LYP/6-311+G(d,p)) of compound **11d** 38

4.5 Optimized xyz coordinates (B3LYP/6-31G(d), SMD, DMSO) and excitation energies and oscillator strengths (B3LYP/6-311+G(d,p)) of compound **11e**...... 41

4.6 Optimized xyz coordinates (B3LYP/6-31G(d), SMD, DMSO) and excitation energies and oscillator strengths (B3LYP/6-311+G(d,p)) of compound **11f** 43

4.7 Optimized xyz coordinates (B3LYP/6-31G(d), SMD, DMSO) and excitation energies and oscillator strengths (B3LYP/6-311+G(d,p)) of compound **11g** 46

4.8 Main orbital contributions for the longest wavelength absorption computed in $CHCl_3$ and PhMe 48

5 References 55

1 Synthesis

The *meso*-phenyl-BODIPY-aldehyde **7** was synthesized according to a procedure reported in the literature (**Scheme S1**).[1]

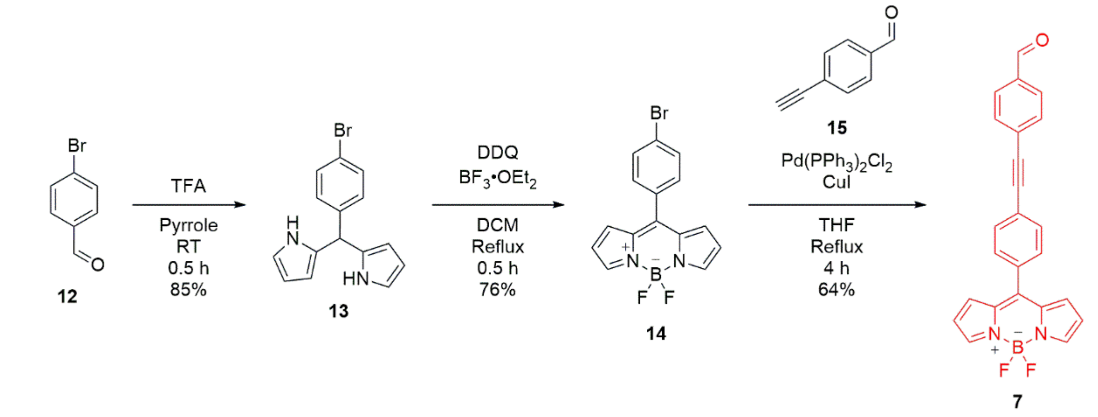

Scheme S1. Synthesis of *meso*-phenyl BODIPY aldehyde

The synthesis of the α-isocyanoacetamide **9** was performed in three reaction steps, according to a method reported in the literature by Zhu and Bienaymé: first, a formylation of racemic phenylalanine (**16**) was carried out using a mixed anhydride to give the *N*-formylphenylalanine (**17**), followed by a peptide coupling if this later one with morpholine (**18**) to give the amide **19**, and lastly, an Ugi-type dehydration to afford the α-isocyanoacetamide **9** in 52% overall yield (**Scheme S2**).[2]

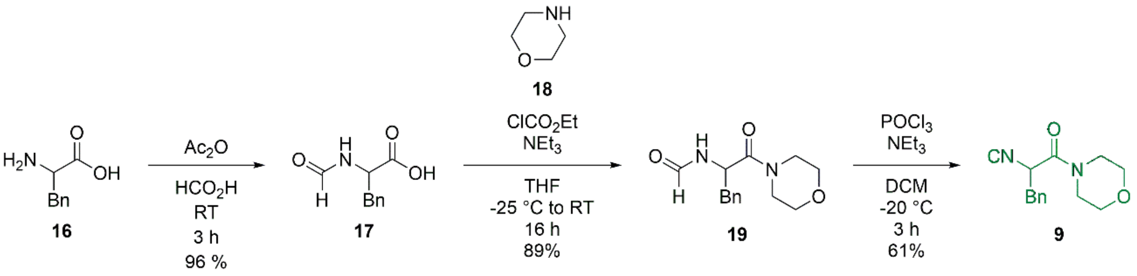

Scheme S2. Synthesis of the α-isocyanoacetamide **9**

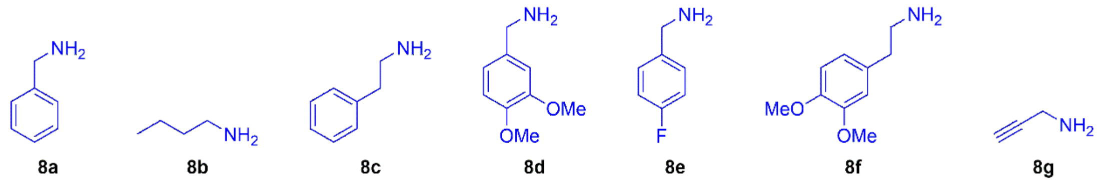

Figure S1. Primary amines used in the synthesis of the compounds

2 NMR and HRMS spectra

2.1 2,6-dibenzyl-7-(4-((4-(5,5-difluoro-5*H*-4λ^4,5λ^4-dipyrrolo[1,2-*c*:2',1'-*f*][1,3,2]diazaborinin-10-yl)phenyl)ethynyl)phenyl)-3-morpholino-6,7-dihydro-5-pyrrolo[3,4-*b*]pyridin-5-one (**11a**)

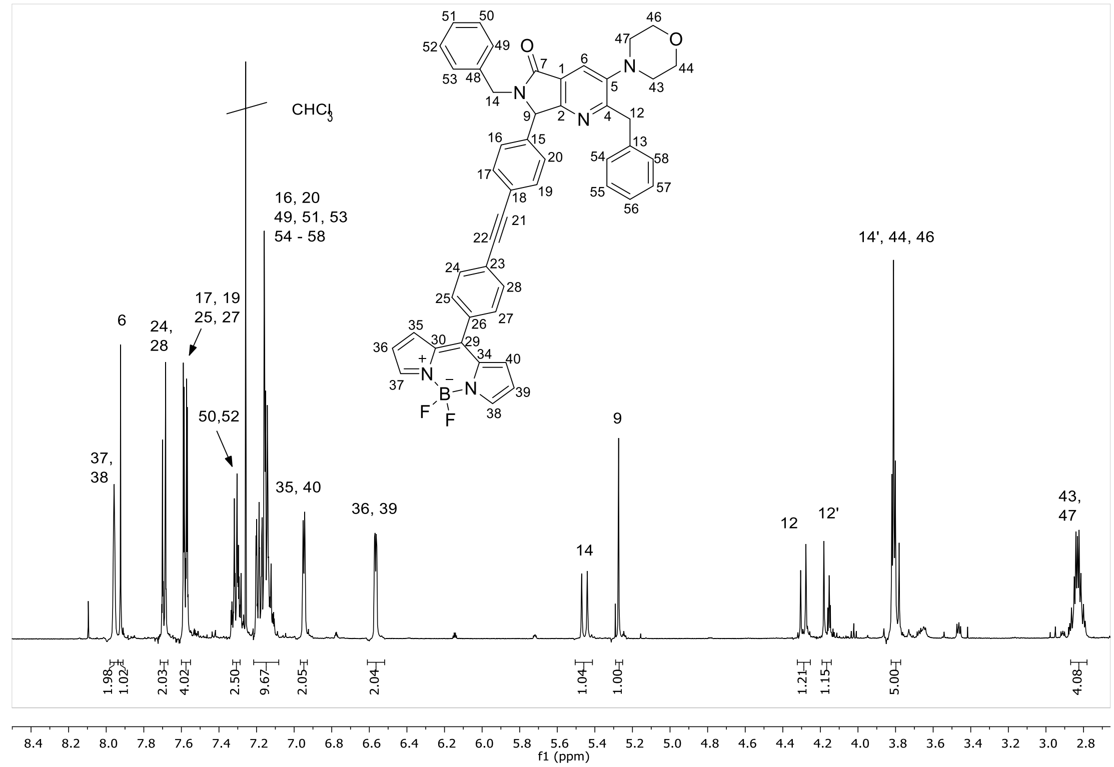

Figure S2. ^{1}H NMR spectrum of **11a**

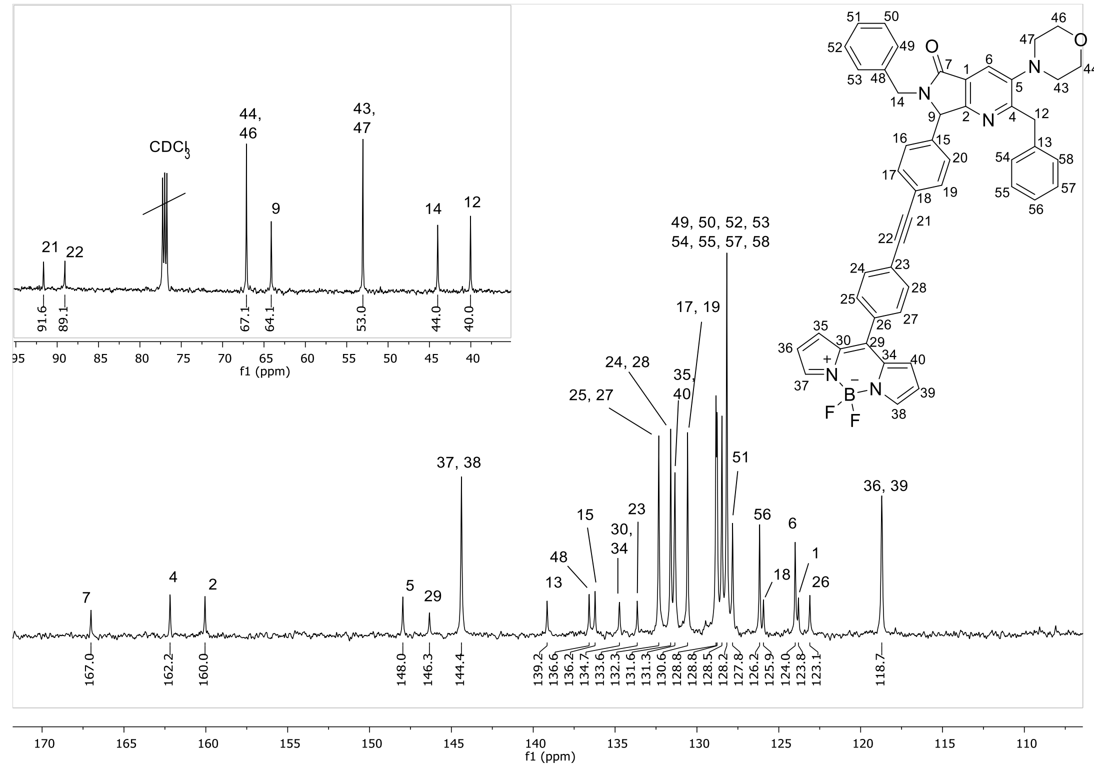

Figure S3. ^{13}C NMR spectrum of compound **11a**

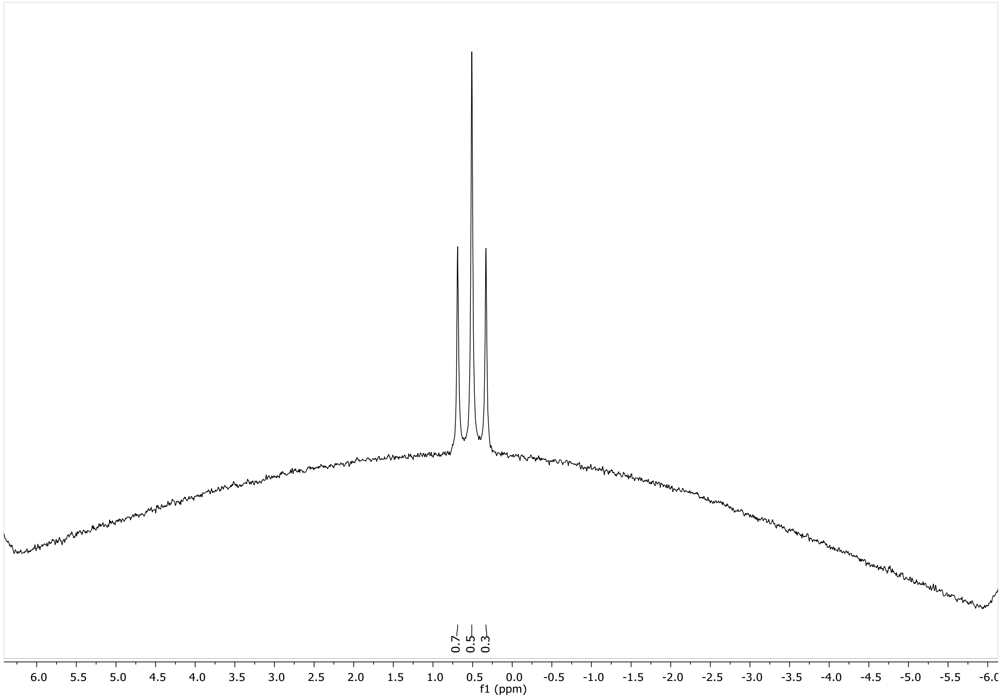

Figure S4. ^{11}B NMR spectrum of compounds **11a**

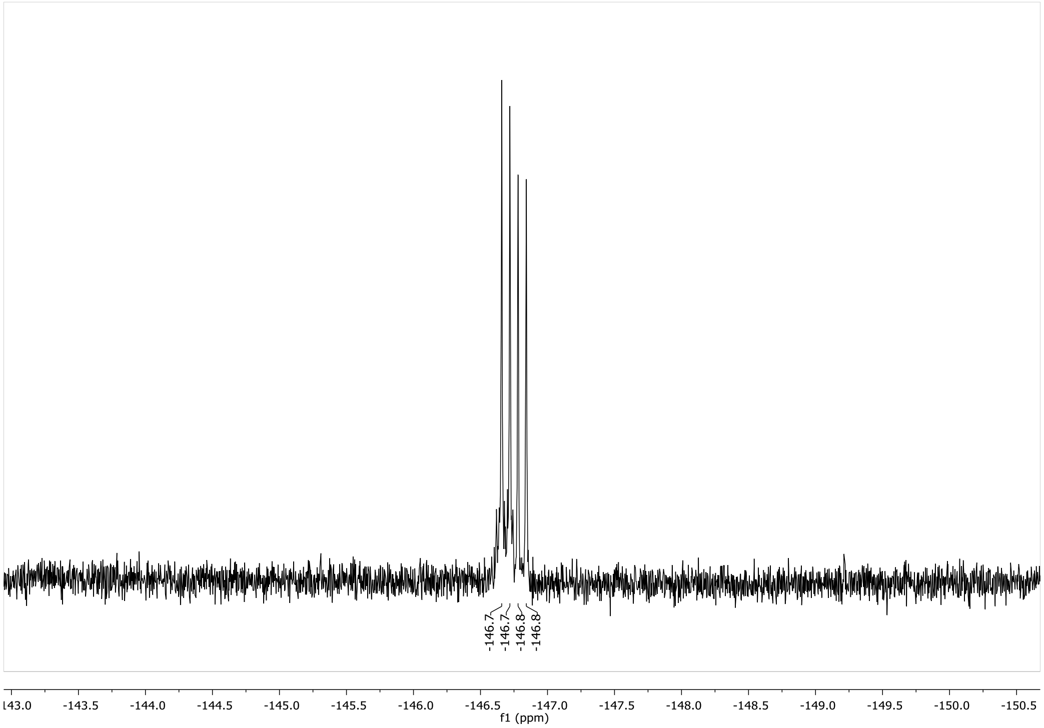

Figure S5. ^{19}F NMR spectrum of compound **11a**

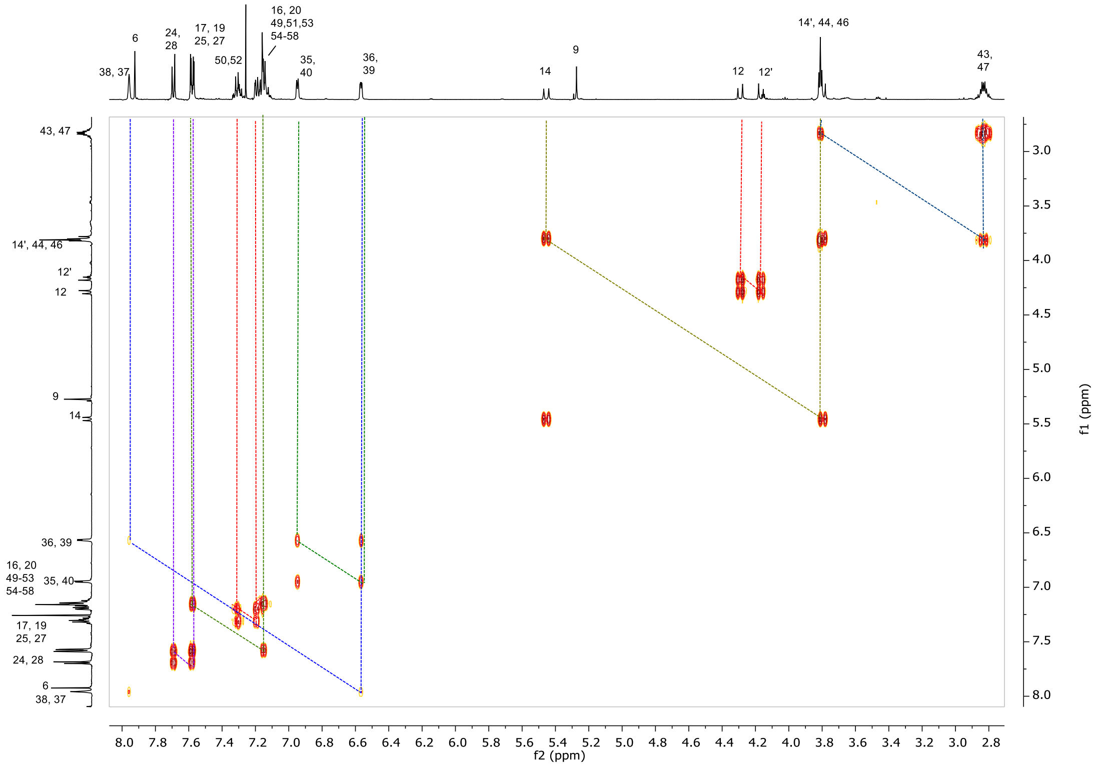

Figure S6. 2D NMR (COSY) spectrum of compound **11a**

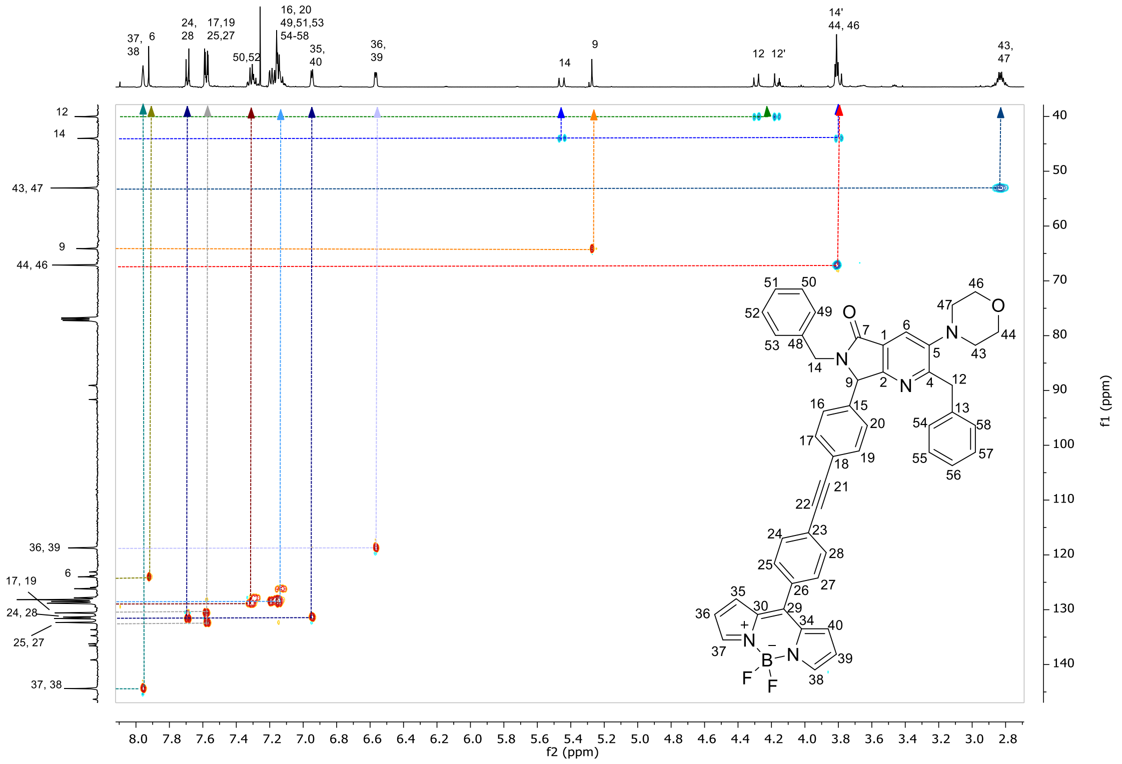

Figure S7. 2D NMR (HSQC) spectrum of compound **11a**

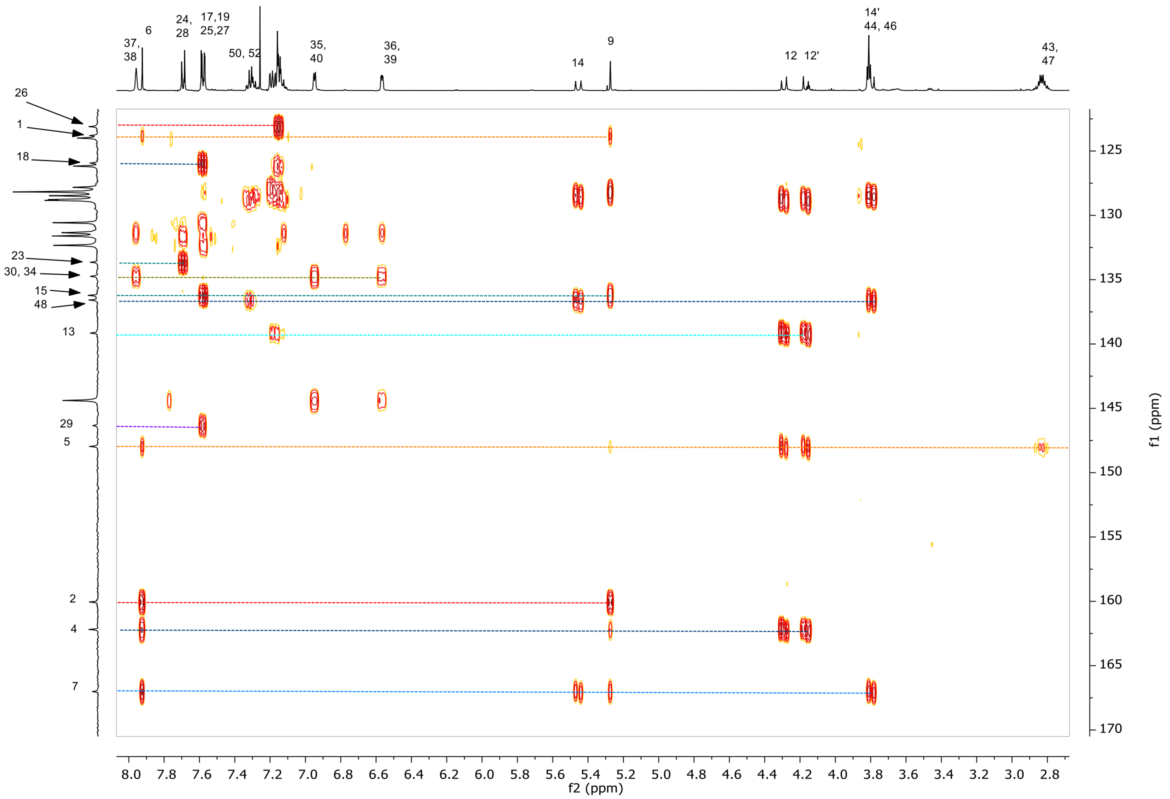

Figure S8. 2D NMR spectrum (HMBC) of compound **11a**

Mass Spectrum SmartFormula Report

Acquisition Parameter

Source Type	ESI	Ion Polarity	Positive	Set Nebulizer	0.5 Bar
Focus	Active			Set Dry Heater	150 °C
Scan Begin	50 m/z	Set Capillary	4500 V	Set Dry Gas	4.0 l/min
Scan End	3000 m/z	Set End Plate Offset	-500 V	Set Divert Valve	Waste

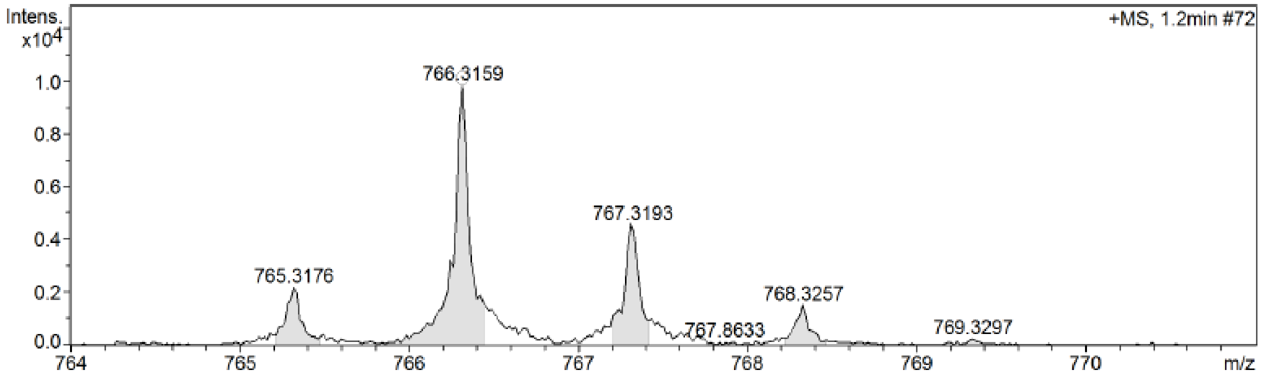

Meas. m/z	#	Ion Formula	Sum Formula	m/z	err [ppm]	|err| [mDa]	mSigma	# mSigma
766.3159	1	C48H39BF2N5O2	C48H38BF2N5O2	766.3159	1.2	0.9	22.2	1

Figure S9. HRMS of compound **11a**

2.2 2-benzyl-6-butyl-7-(4-((4-(5,5-difluoro-5*H*-4λ^4,5λ^4-dipyrrolo[1,2-*c*:2',1'-*f*][1,3,2]diazaborinin-10-yl)phenyl)ethynyl)phenyl)-3-morpholino-6,7-dihydro-5*H*-pyrrolo[3,4-*b*]pyridin-5-one (**11b**)

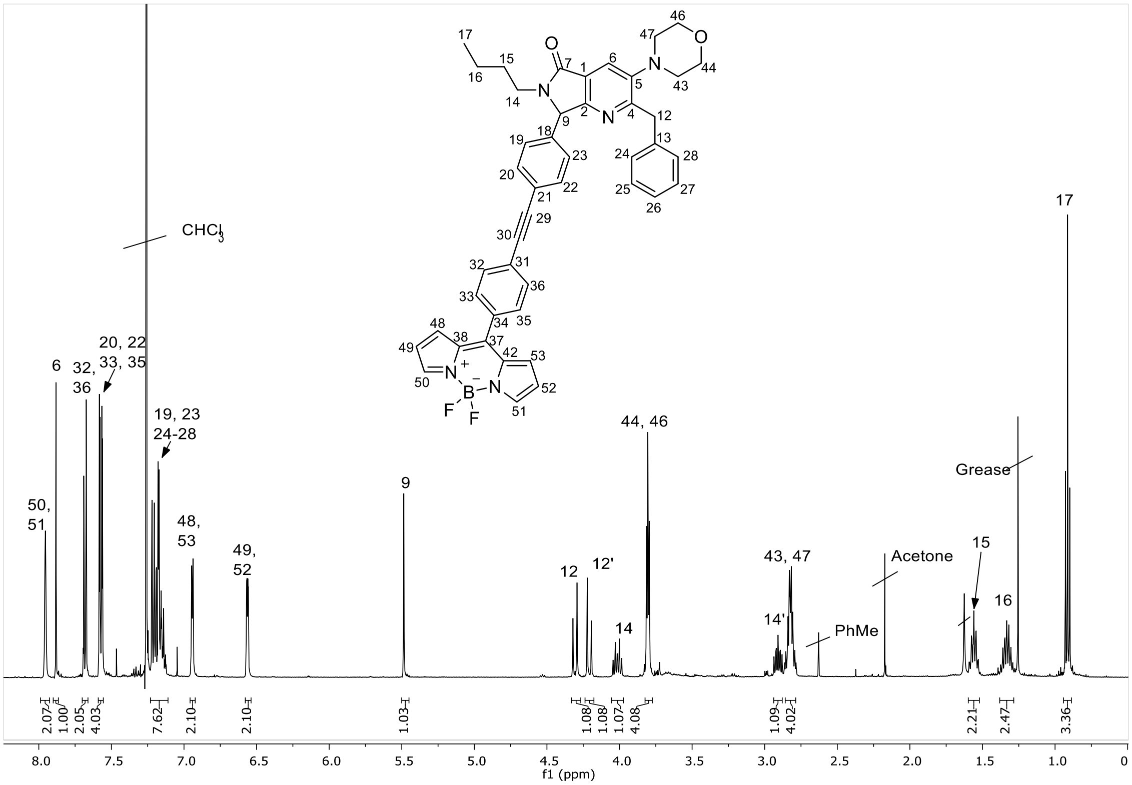

Figure S10. ^{1}H NMR spectrum of **11b**

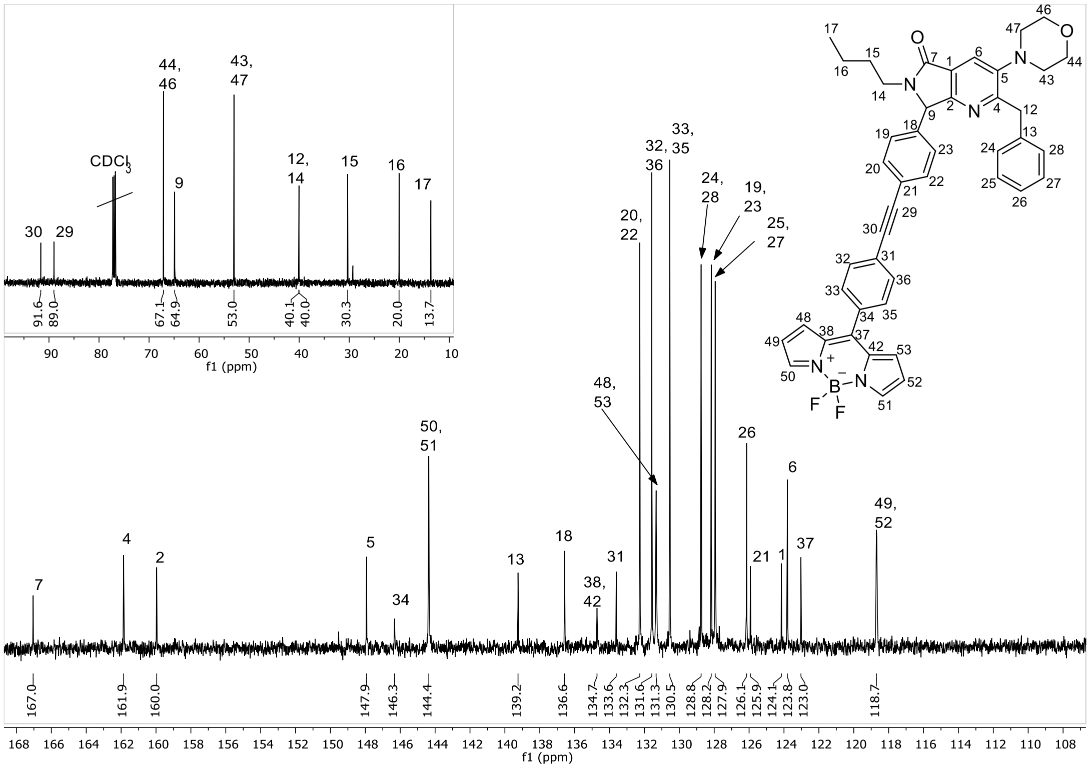

Figure S11. ^{13}C NMR spectrum of **11b**

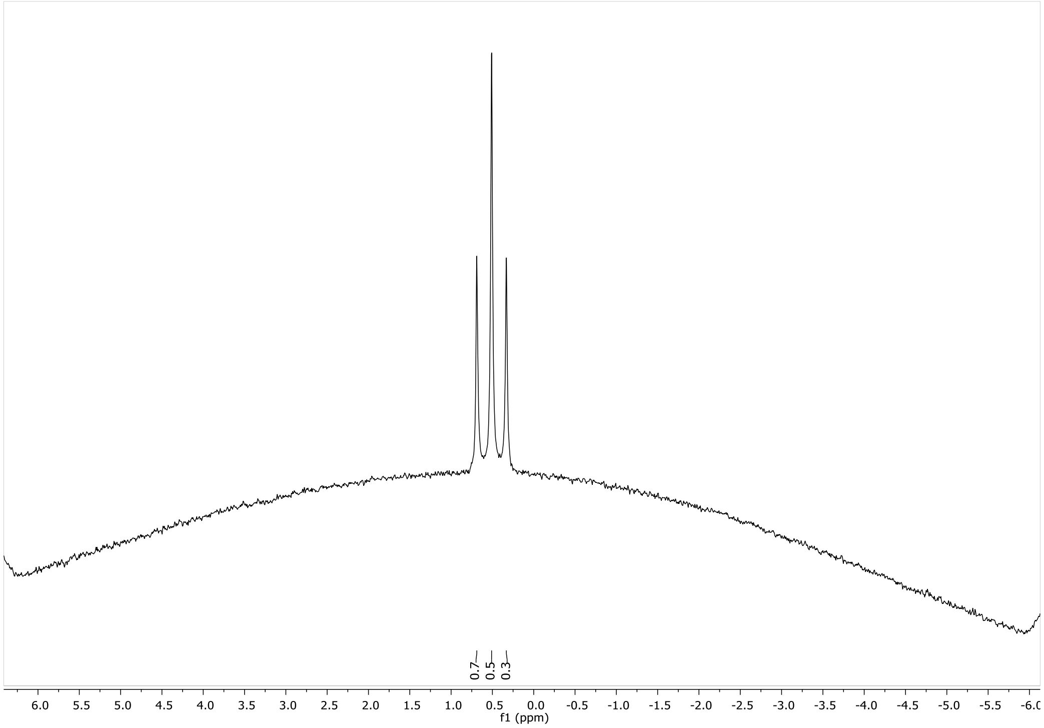

Figure S12. ^{11}B NMR spectrum of **11b**

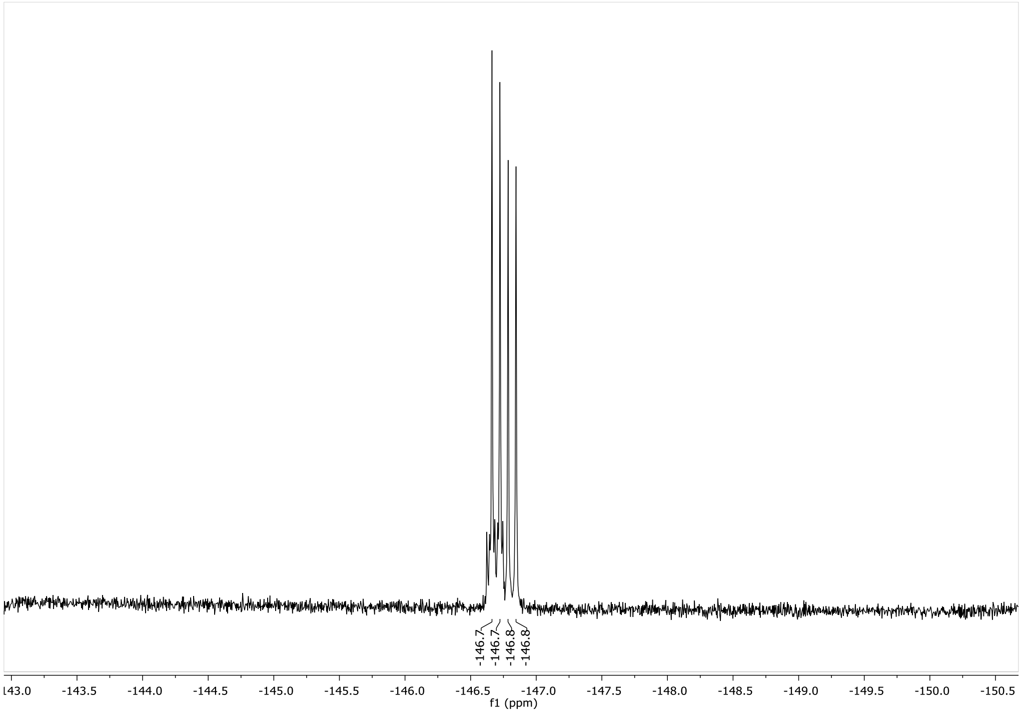

Figure S13. ^{19}F NMR spectrum of **11b**

Mass Spectrum SmartFormula Report

Acquisition Parameter

Source Type	ESI	Ion Polarity	Positive	Set Nebulizer	0.5 Bar
Focus	Active			Set Dry Heater	150 °C
Scan Begin	50 m/z	Set Capillary	4500 V	Set Dry Gas	4.0 l/min
Scan End	3000 m/z	Set End Plate Offset	-500 V	Set Divert Valve	Waste

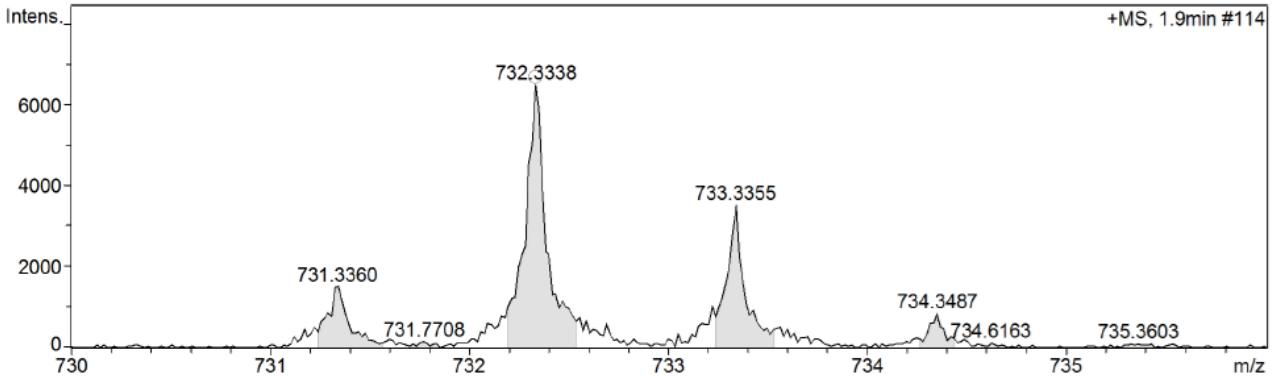

Meas. m/z	#	Ion Formula	Sum Formula	m/z	err [ppm]	|err| [mDa]	mSigma	# mSigma
732.3338	1	C45H41BF2N5O2	C45H40BF2N5O2	732.3316	-2.0	1.4	29.7	1

Figure S14. HRMS spectrum of compound **11b**

2.3 2-benzyl-7-(4-((4-(5,5-difluoro-5*H*-4λ[4],5λ[4]-dipyrrolo[1,2-*c*:2',1'-*f*][1,3,2]diazaborinin-10-yl)phenyl)ethynyl)phenyl)-3-morpholino-6-phenethyl-6,7-dihydro-5*H*-pyrrolo[3,4-*b*]pyridin-5-one (**11c**)

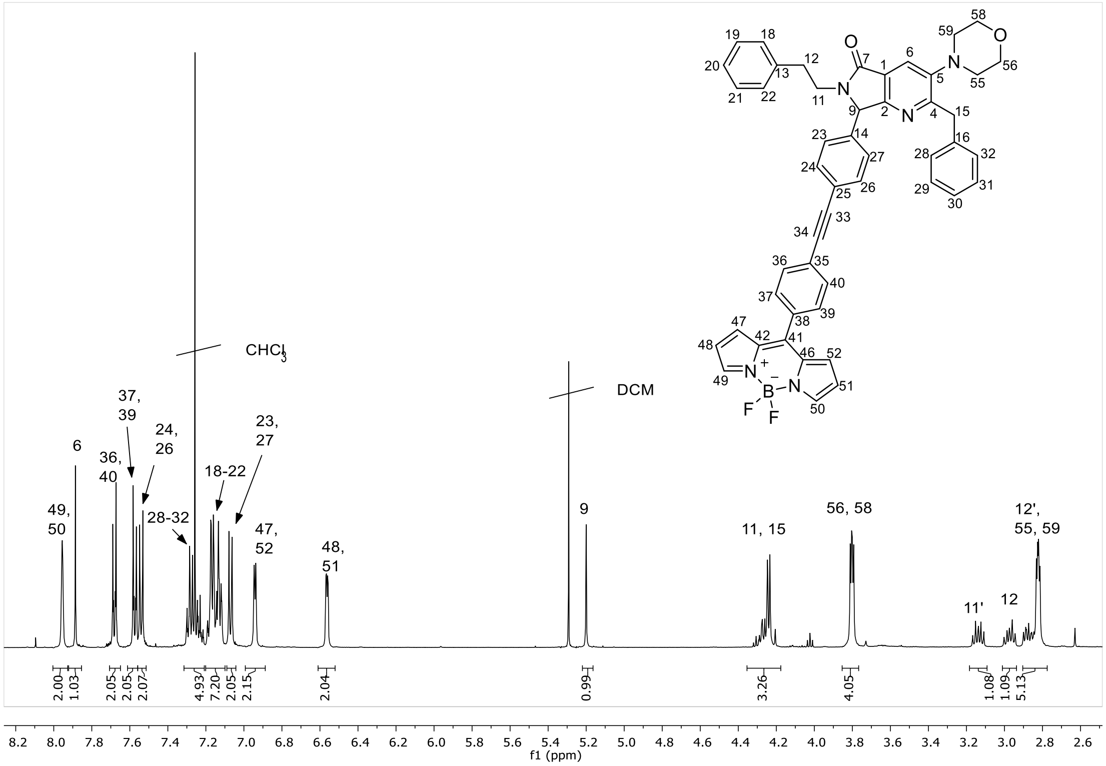

Figure 15. ^{1}H NMR spectrum of compound **11c**

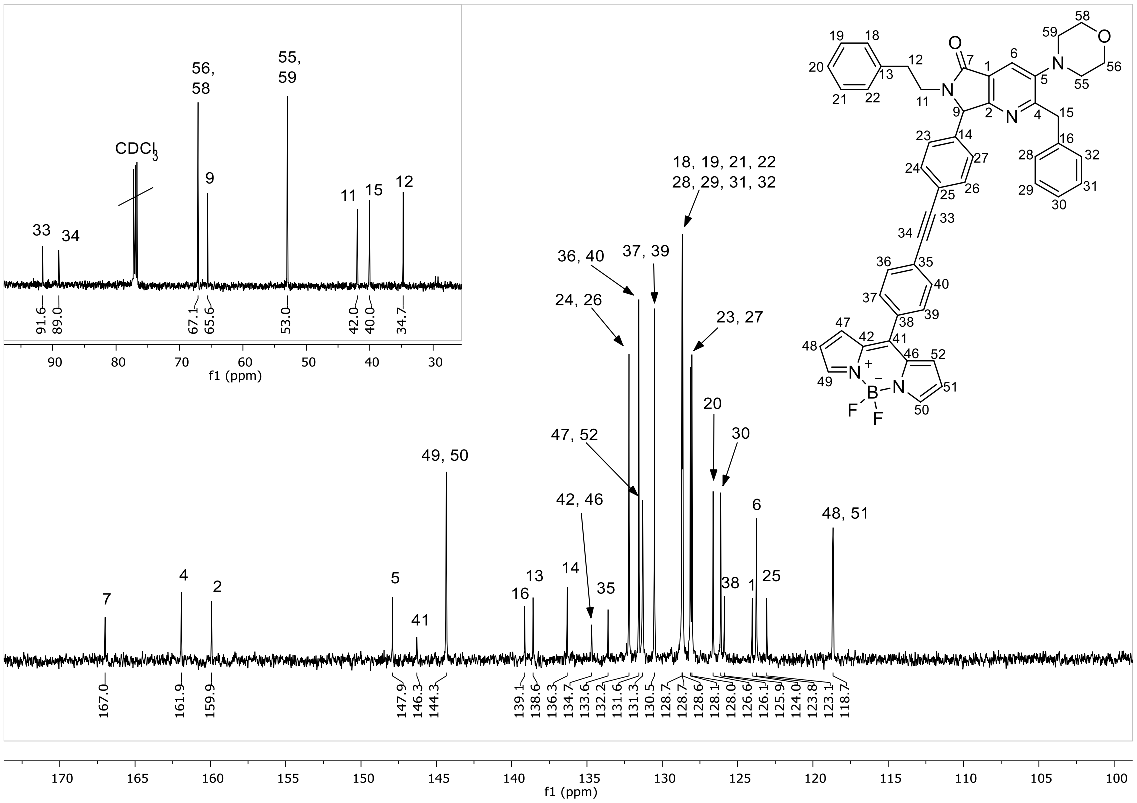

Figure 16. ^{13}C NMR spectrum of compound **11c**

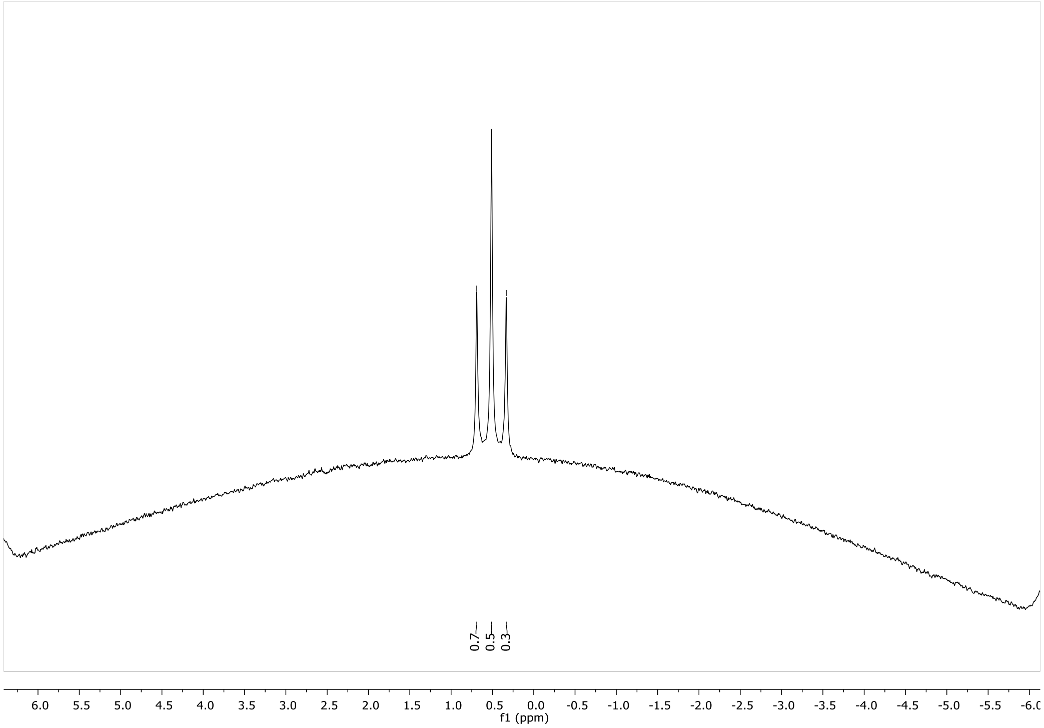

Figure S17. ^{11}B NMR spectrum of compound **11c**

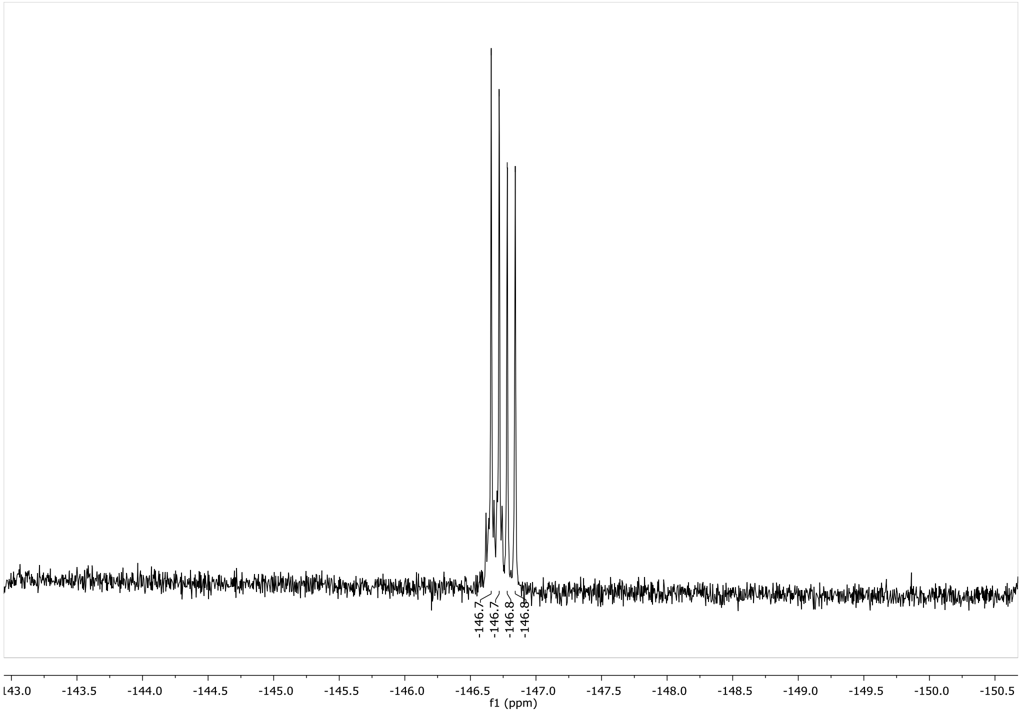

Figure S18. ^{19}F NMR spectrum of compound **11c**

Mass Spectrum SmartFormula Report

Acquisition Parameter

Source Type	ESI	Ion Polarity	Positive	Set Nebulizer	0.5 Bar
Focus	Active			Set Dry Heater	150 °C
Scan Begin	50 m/z	Set Capillary	4500 V	Set Dry Gas	4.0 l/min
Scan End	3000 m/z	Set End Plate Offset	-500 V	Set Divert Valve	Waste

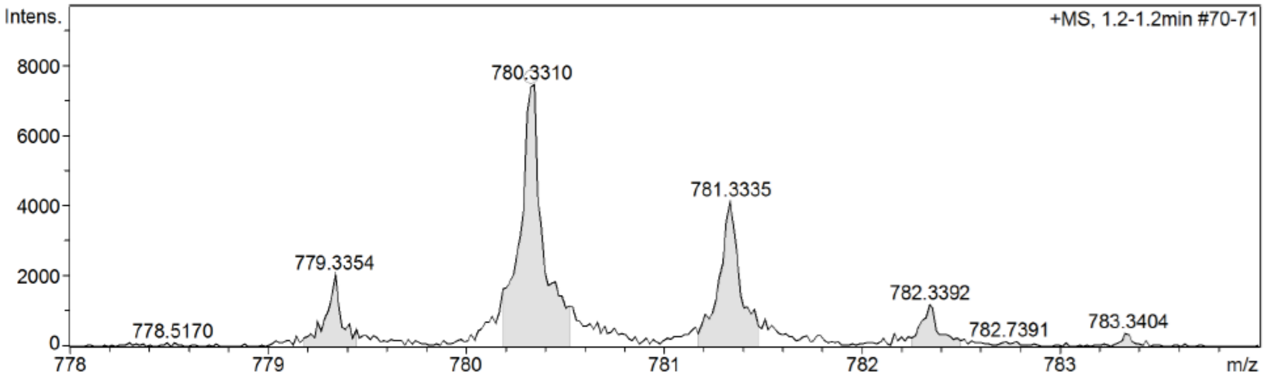

Meas. m/z	#	Ion Formula	Sum Formula	m/z	err [ppm]	|err| [mDa]	mSigma	# mSigma	N/C
780.3310	1	C49H41BF2N5O2	C49H40BF2N5O2	780.3316	1.8	1.4	33.4	1	0.102
802.3134	1	C49H40BF2N5NaO2		802.3135	1.2	1.0	64.7	1	0.102

Figure S19. HRMS spectrum of compound **11c**

2.4 2-benzyl-7-(4-((4-(5,5-difluoro-5*H*-$4\lambda^4$,$5\lambda^4$-dipyrrolo[1,2-*c*:2',1'-*f*][1,3,2]diazaborinin-10-yl)phenyl)ethynyl)phenyl)-6-(3,4-dimethoxybenzyl)-3-morpholino-6,7-dihydro-5*H*-pyrrolo[3,4-*b*]pyridin-5-one (**11d**)

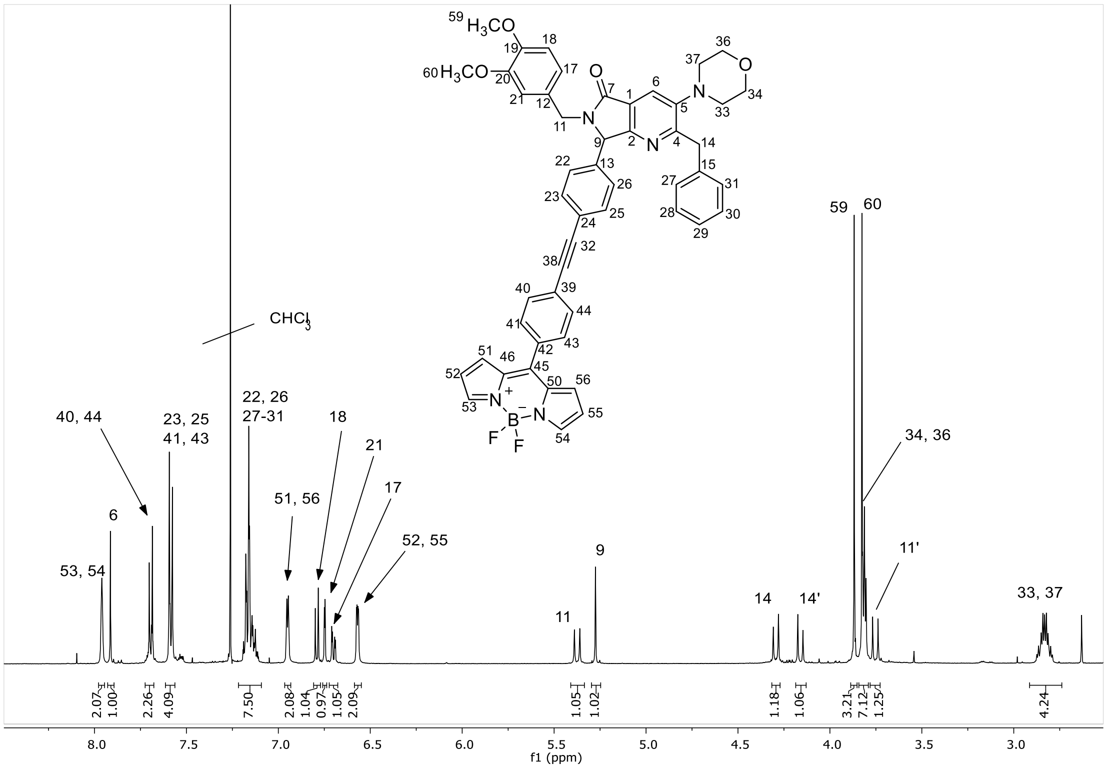

Figure S20. ^{1}H NMR spectrum of compound **11d**

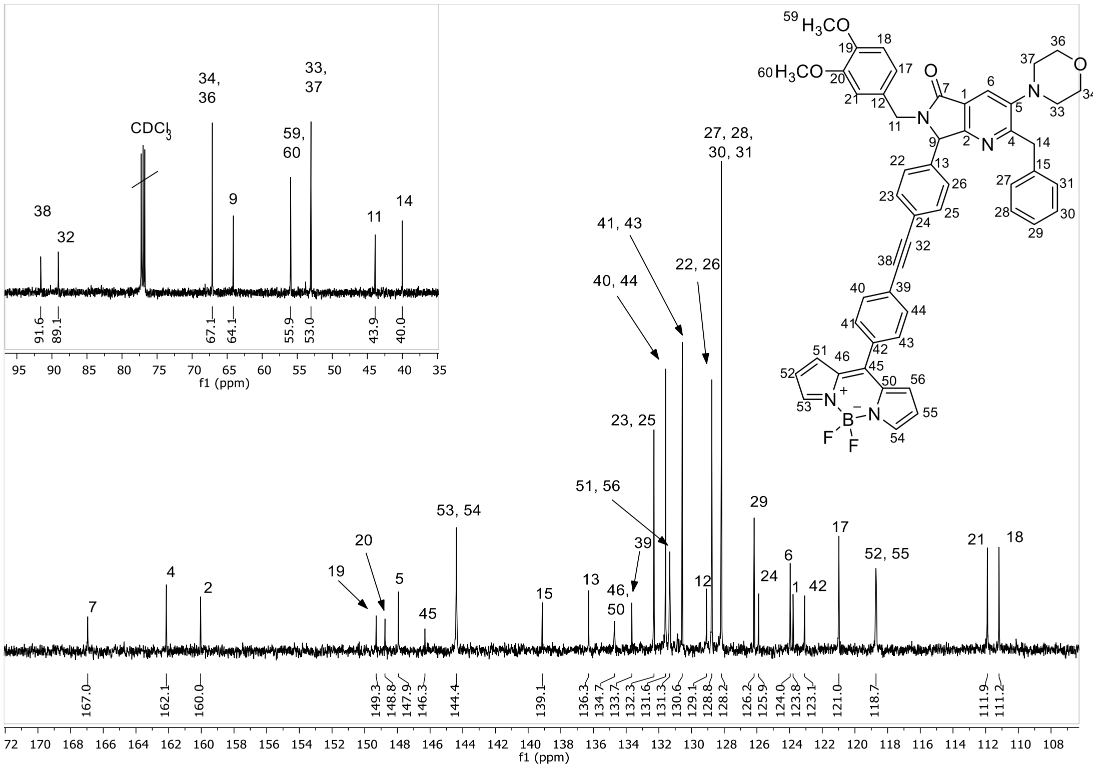

Figure S21. ^{13}C NMR spectrum of compound **11d**

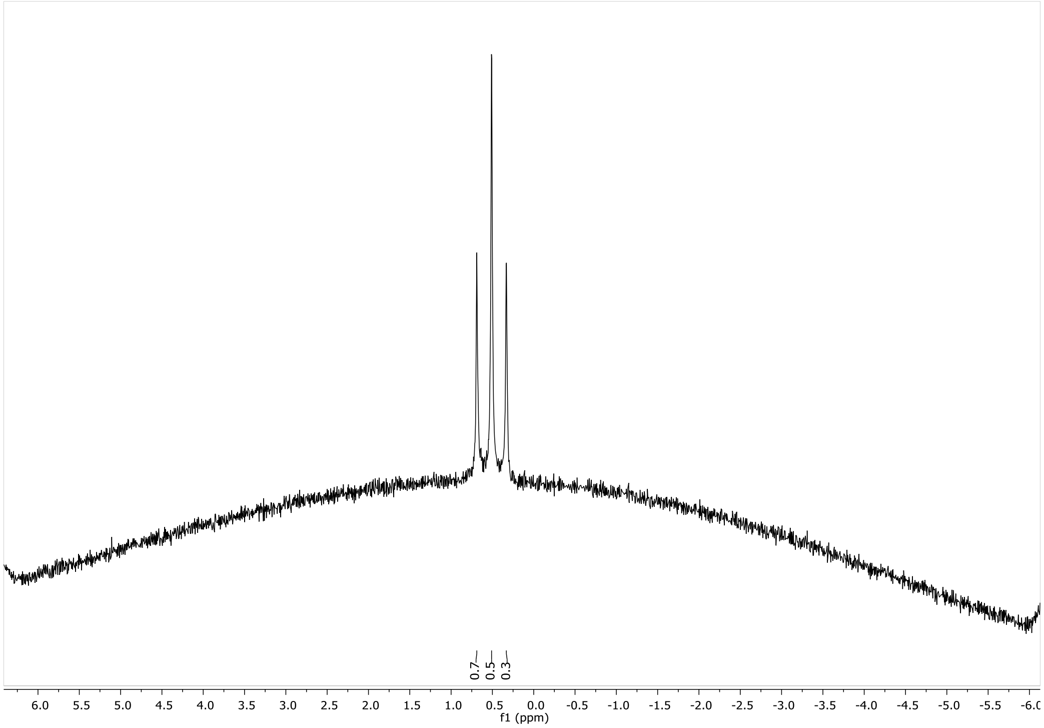

Figure S22. ^{11}B NMR spectrum of compound **11d**

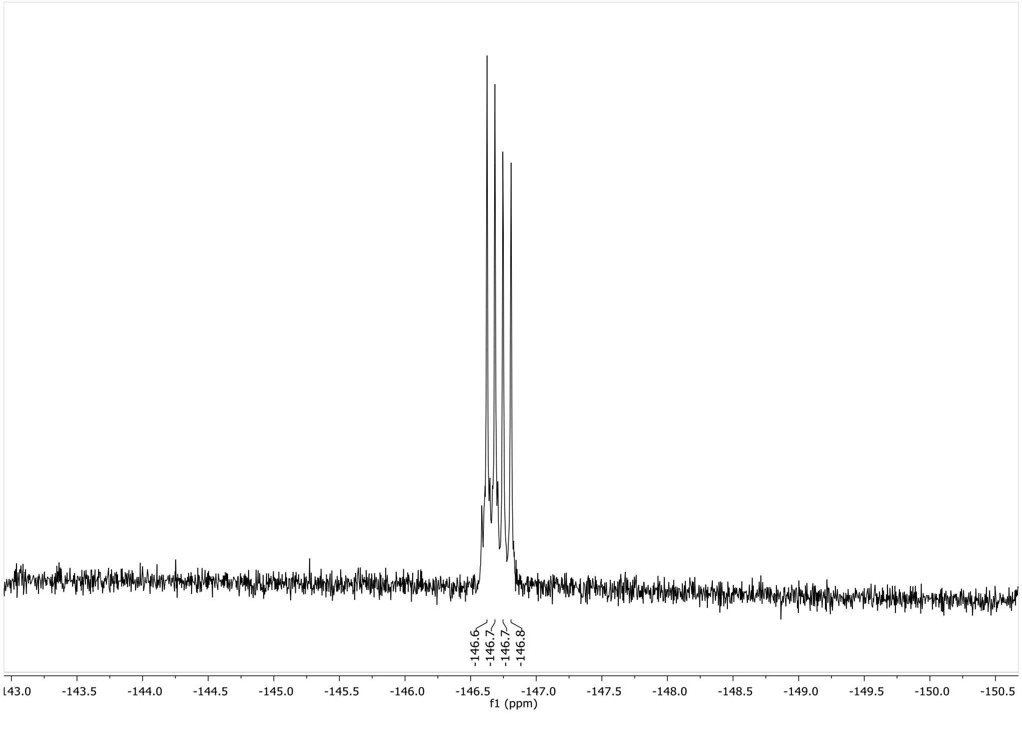

Figure S23. ^{19}F NMR spectrum of compound **11d**

Mass Spectrum SmartFormula Report

Acquisition Parameter

Source Type	ESI	Ion Polarity	Positive	Set Nebulizer	0.5 Bar
Focus	Active			Set Dry Heater	150 °C
Scan Begin	50 m/z	Set Capillary	4500 V	Set Dry Gas	4.0 l/min
Scan End	3000 m/z	Set End Plate Offset	-500 V	Set Divert Valve	Waste

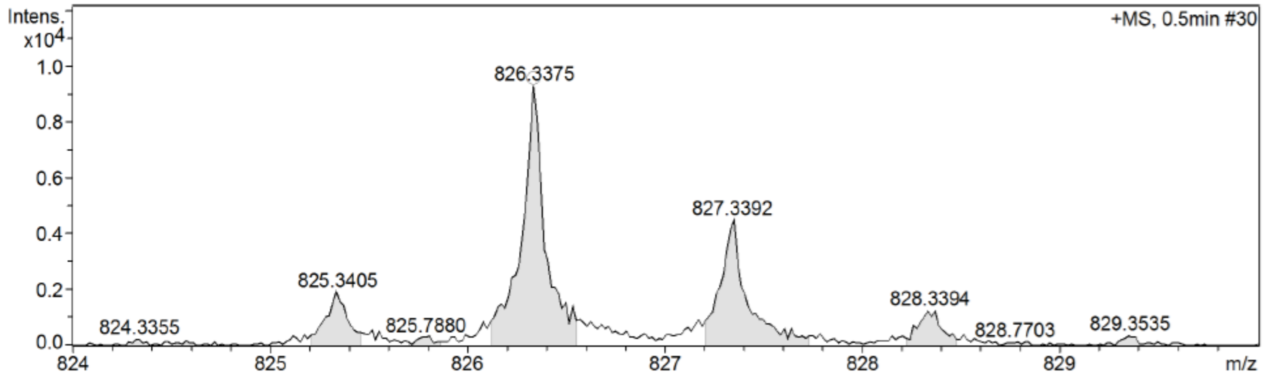

Meas. m/z	#	Ion Formula	Sum Formula	m/z	err [ppm]	|err| [mDa]	mSigma	# mSigma
826.3375	1	C50H43BF2N5O4	C50H42BF2N5O4	826.3371	0.5	0.4	24.1	1
848.3212	1	C50H42BF2N5NaO4		848.3190	-1.6	1.4	72.7	1

Figure S24. HRMS spectrum of compound **11d**

2.5 2-benzyl-7-(4-((4-(5,5-difluoro-5*H*-4λ^4,5λ^4-dipyrrolo[1,2-*c*:2',1'-*f*][1,3,2]diazaborinin-10-yl)phenyl)ethynyl)phenyl)-6-(4-fluorobenzyl)-3-morpholino-6,7-dihydro-5*H*-pyrrolo[3,4-*b*]pyridin-5-one (**11e**)

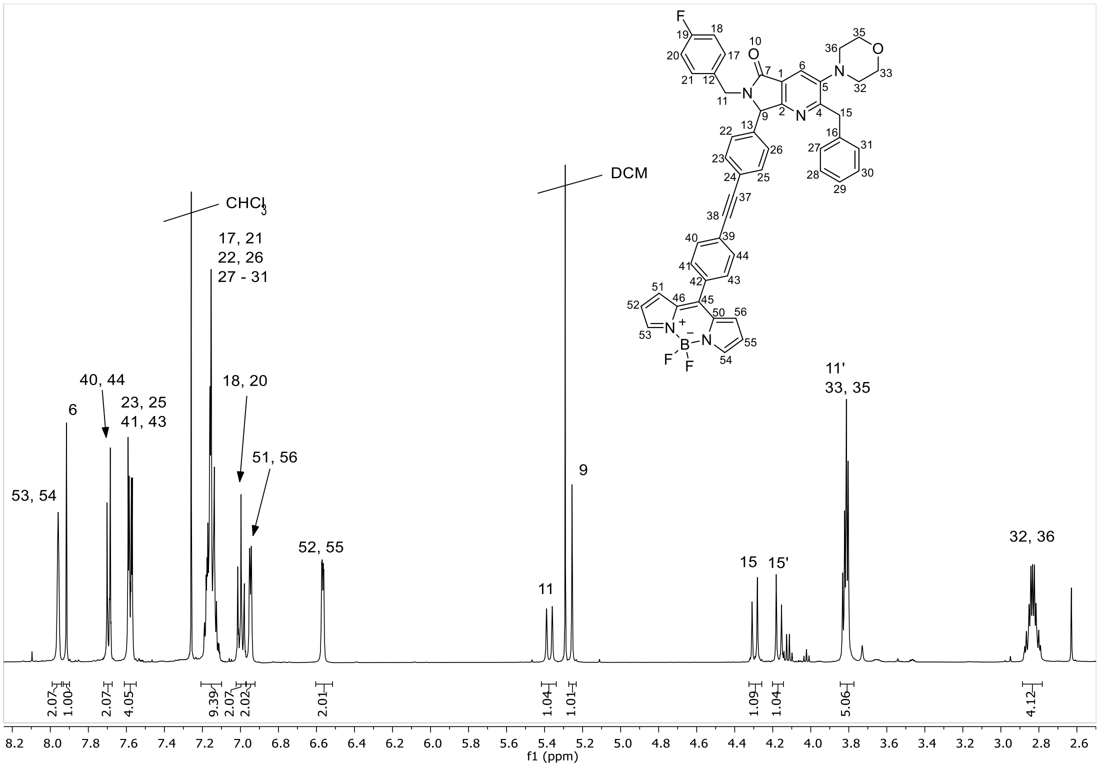

Figure 25. ^{1}H NMR spectrum of compound **11e**

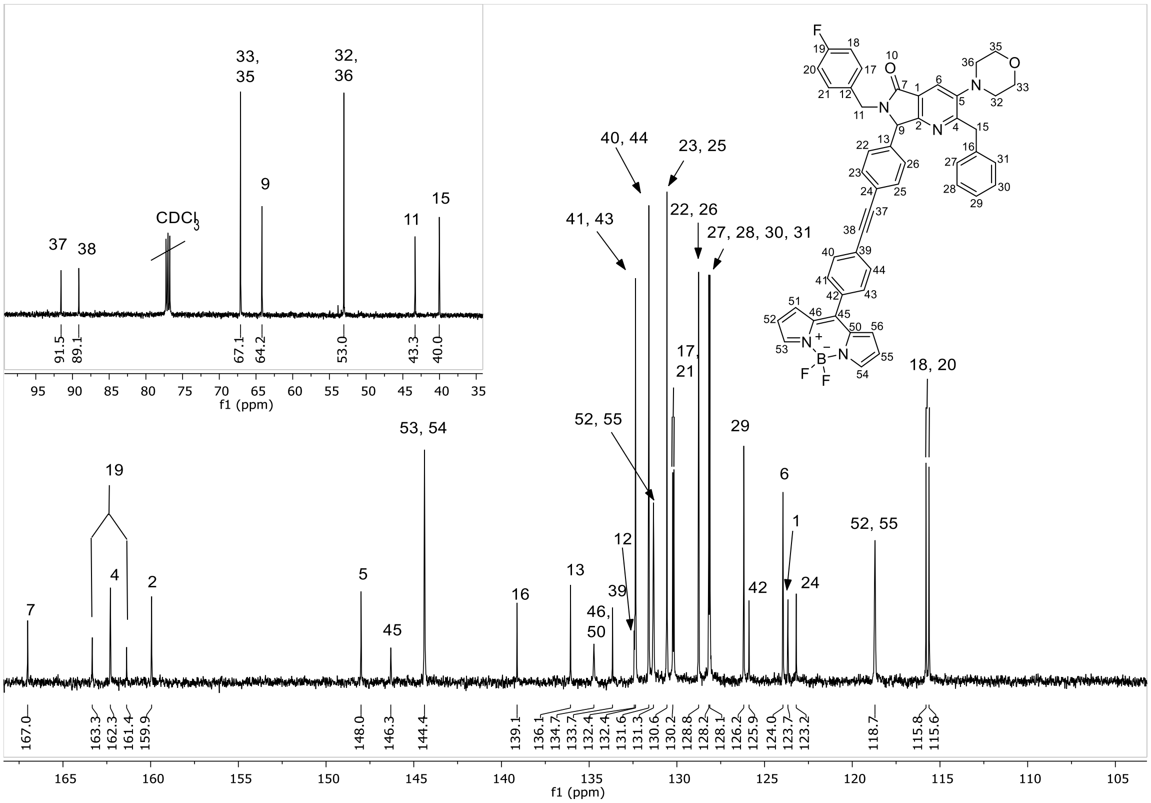

Figure S26. ^{13}C NMR spectrum of compound **11e**

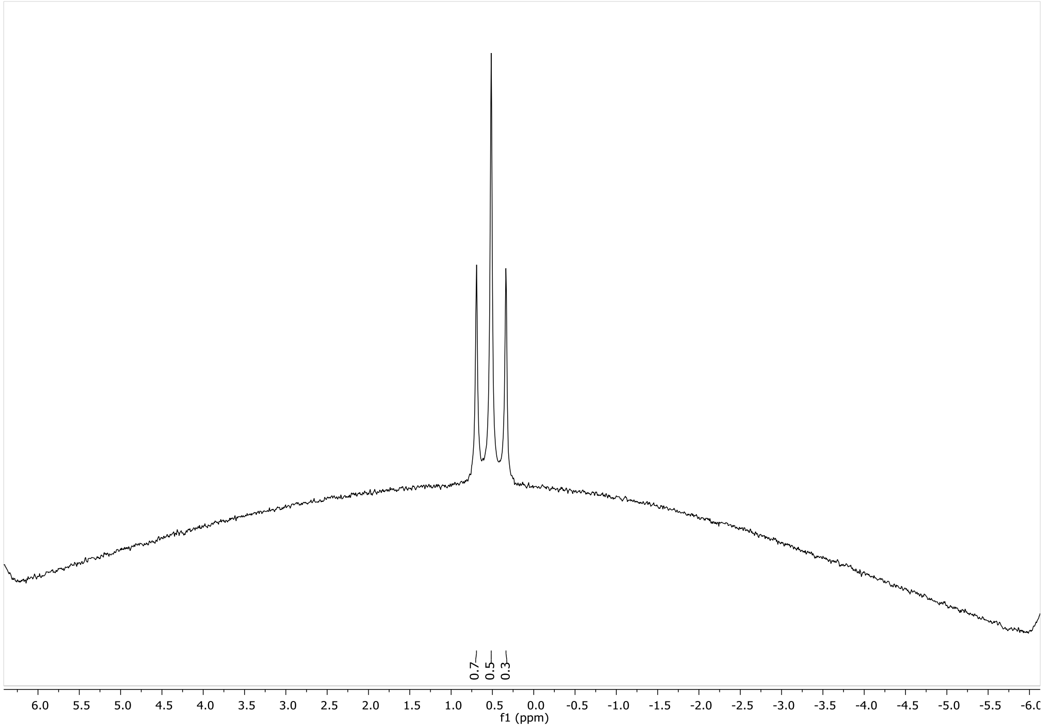

Figure S27. ^{11}B NMR spectrum of compound **11e**

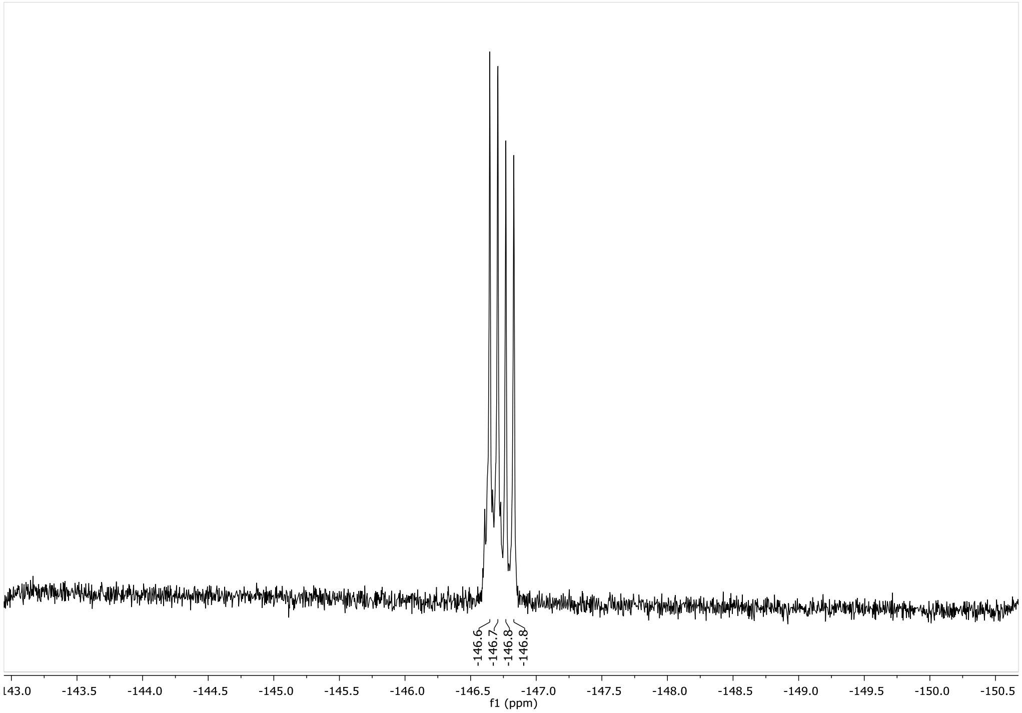

Figure S28. ^{19}F NMR spectrum of compound **11e**

Mass Spectrum SmartFormula Report

Acquisition Parameter

Source Type	ESI	Ion Polarity	Positive	Set Nebulizer	0.5 Bar
Focus	Active			Set Dry Heater	150 °C
Scan Begin	50 m/z	Set Capillary	4500 V	Set Dry Gas	4.0 l/min
Scan End	3000 m/z	Set End Plate Offset	-500 V	Set Divert Valve	Waste

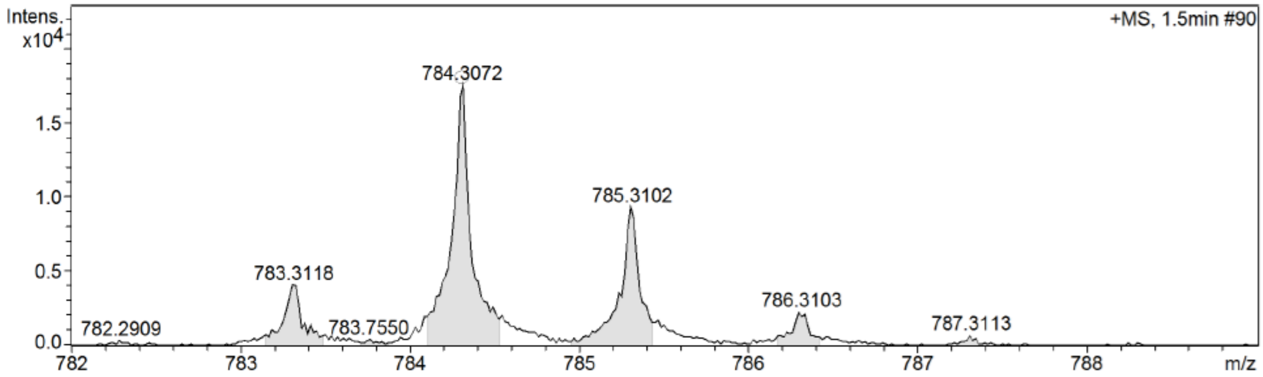

Meas. m/z	#	Ion Formula	Sum Formula	m/z	err [ppm]	|err| [mDa]	mSigma	# mSigma
784.3072	1	C48H38BF3N5O2	C48H37BF3N5O2	784.3065	0.2	0.1	13.6	1
806.2910	1	C48H37BF3N5NaO2		806.2885	-2.2	1.7	32.7	1

Figure S29. HRMS spectrum of compound **11e**

2.6 2-benzyl-7-(4-((4-(5,5-difluoro-5*H*-4λ4,5λ4-dipyrrolo[1,2-*c*:2',1'-*f*][1,3,2]diazaborinin-10-yl)phenyl)ethynyl)phenyl)-6-(3,4-dimethoxyphenethyl)-3-morpholino-6,7-dihydro-5*H*-pyrrolo[3,4-*b*]pyridin-5-one (**11f**)

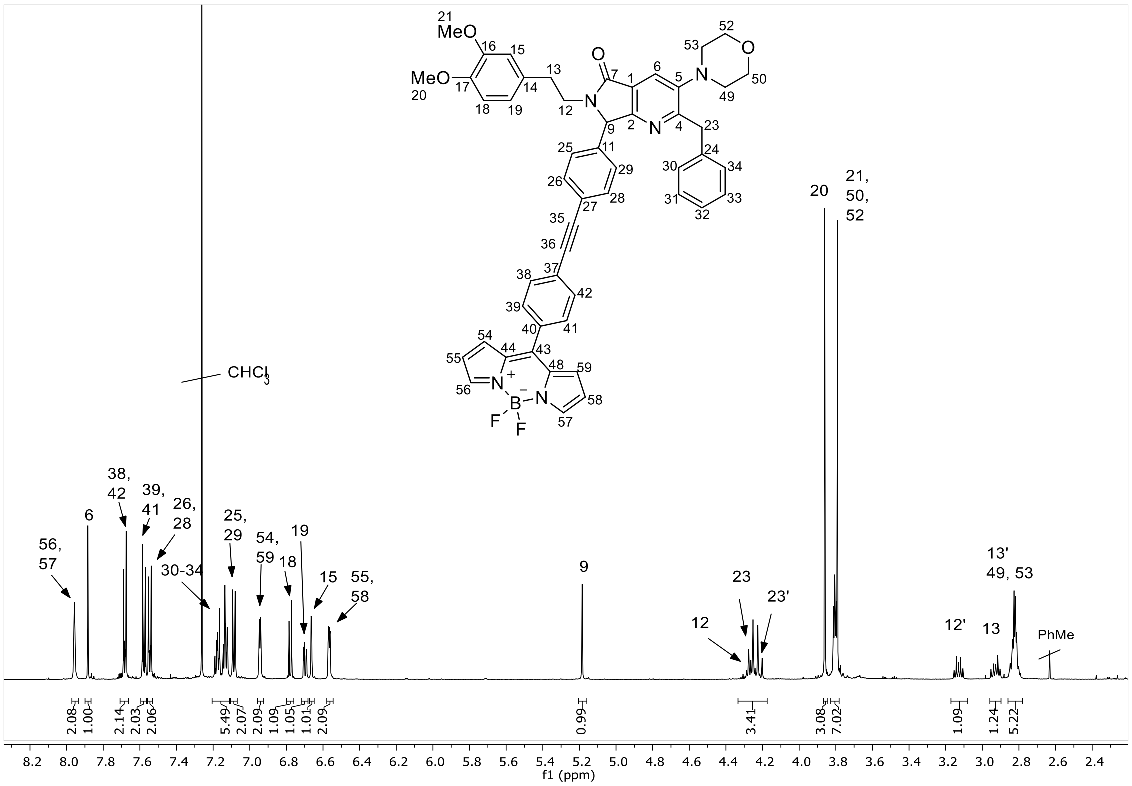

Figure S30. ^{1}H NMR spectrum of compound **11f**

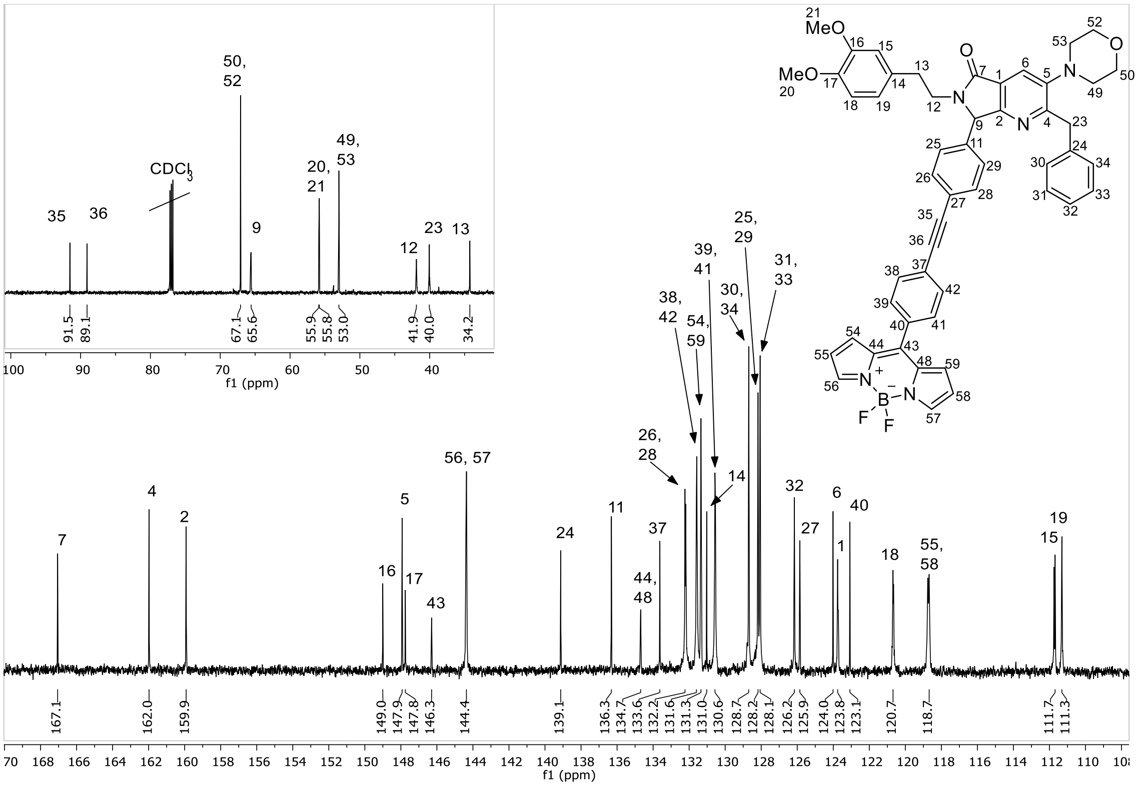

Figure S31. ^{13}C NMR spectrum of compound **11f**

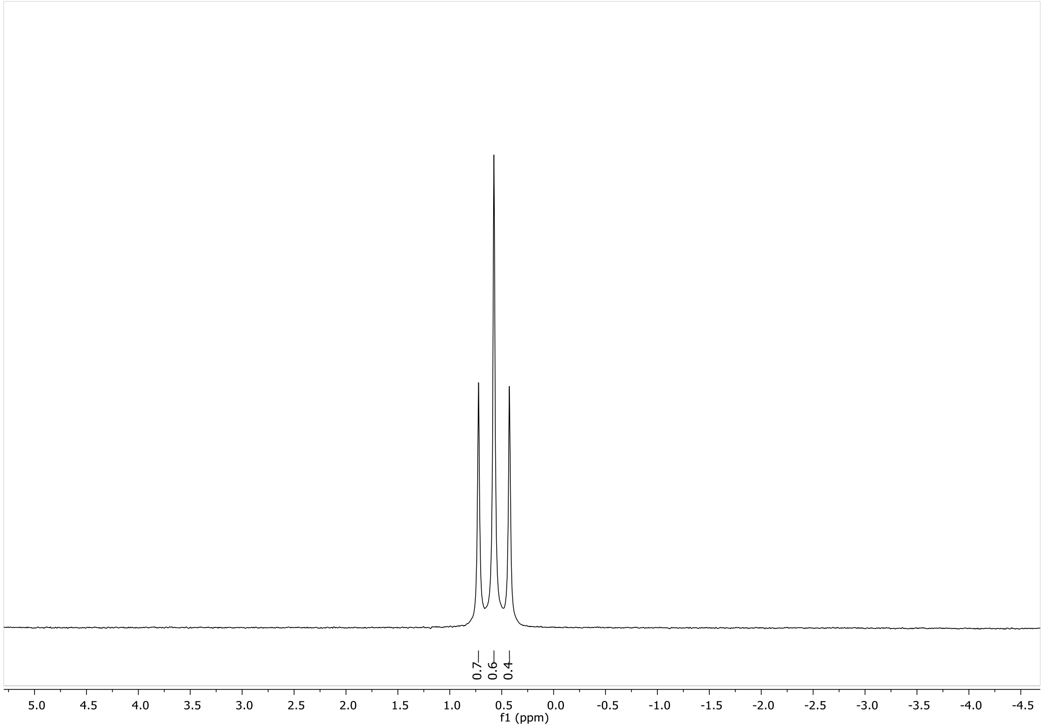

Figure S32. ^{11}B NMR spectrum of compound **11f**

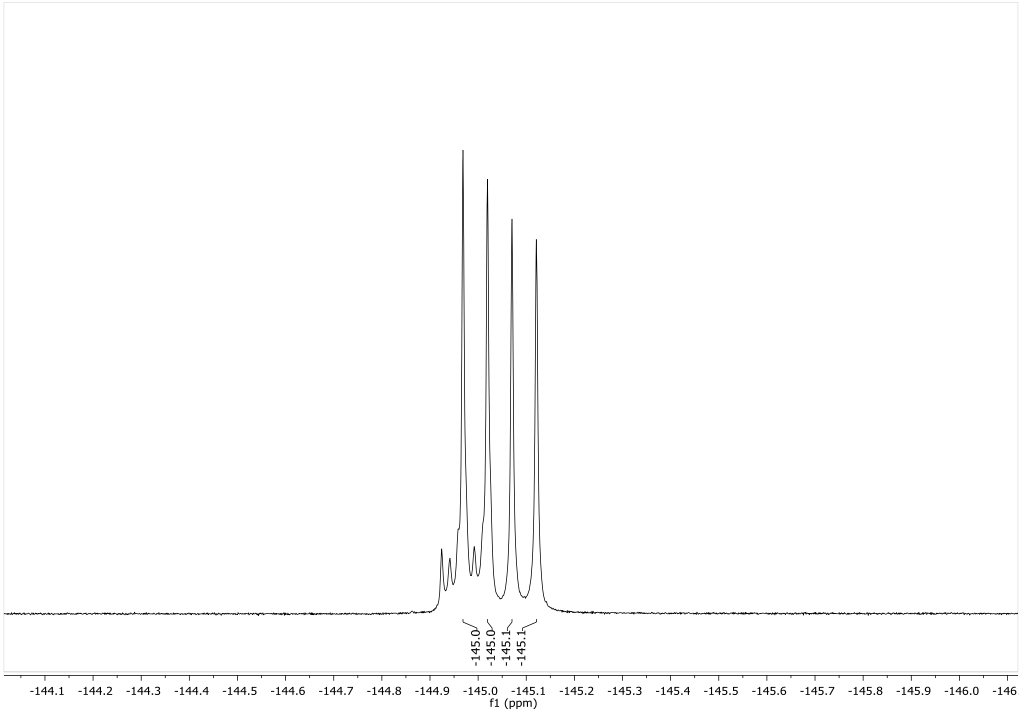

Figure S33. ^{19}F NMR spectrum of compound **11f**

Mass Spectrum SmartFormula Report

Acquisition Parameter

Source Type	ESI	Ion Polarity	Positive	Set Nebulizer	0.5 Bar
Focus	Active			Set Dry Heater	150 °C
Scan Begin	50 m/z	Set Capillary	4500 V	Set Dry Gas	4.0 l/min
Scan End	3000 m/z	Set End Plate Offset	-500 V	Set Divert Valve	Waste

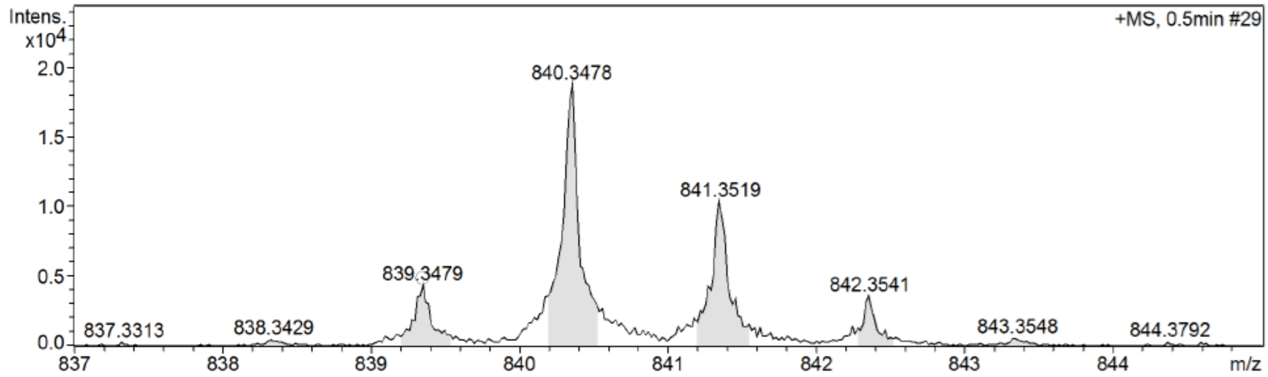

Meas. m/z	#	Ion Formula	Sum Formula	m/z	err [ppm]	|err| [mDa]	mSigma	# mSigma
839.3479	1	C51H44BF2N5O4	C51H44BF2N5O4	839.3449	-2.6	2.2	450.5	1
	1	C51H44BF2N5O4	C51H43BF2N5O4	839.3449	-2.6	2.2	450.5	1

Figure S34. HRMS spectrum of compound **11f**

2.7 2-benzyl-7-(4-((4-(5,5-difluoro-5*H*-4λ4,5λ4-dipyrrolo[1,2-*c*:2',1'-*f*][1,3,2]diazaborinin-10-yl)phenyl)ethynyl)phenyl)-3-morpholino-6-(prop-2-yn-1-yl)-6,7-dihidro-5*H*-pyrrolo[3,4-*b*]pyridin-5-one (**11g**)

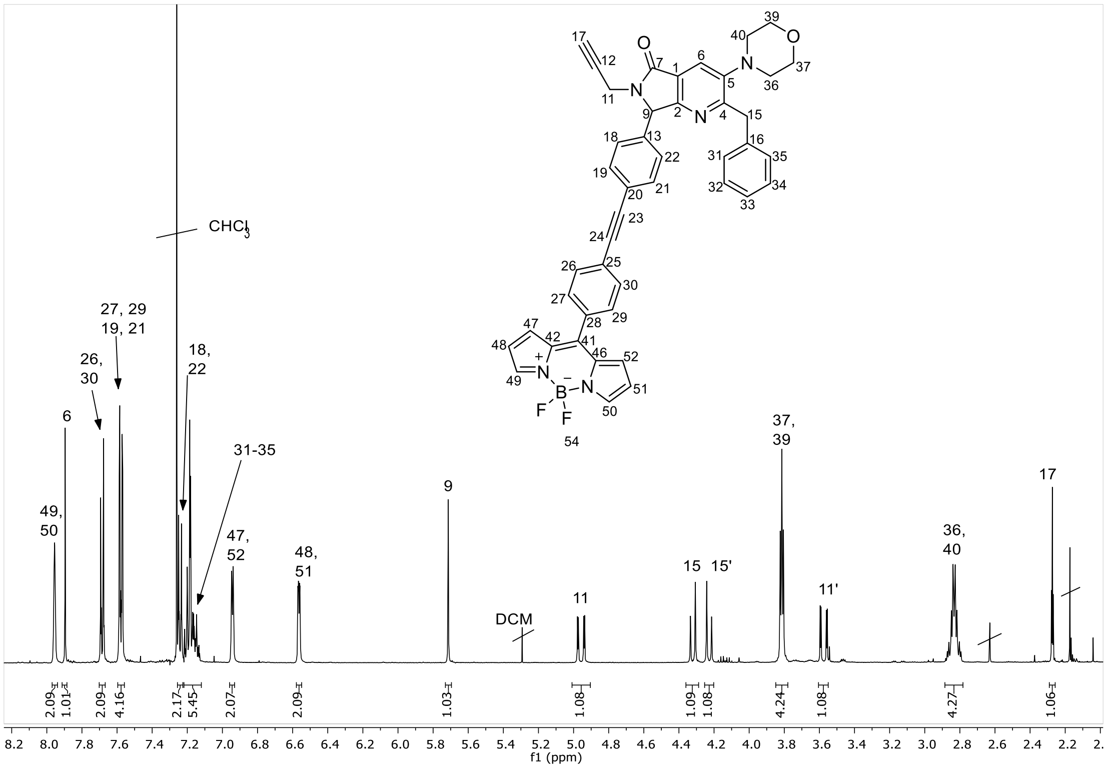

Figure 35. ^{1}H NMR spectrum of compound **11g**

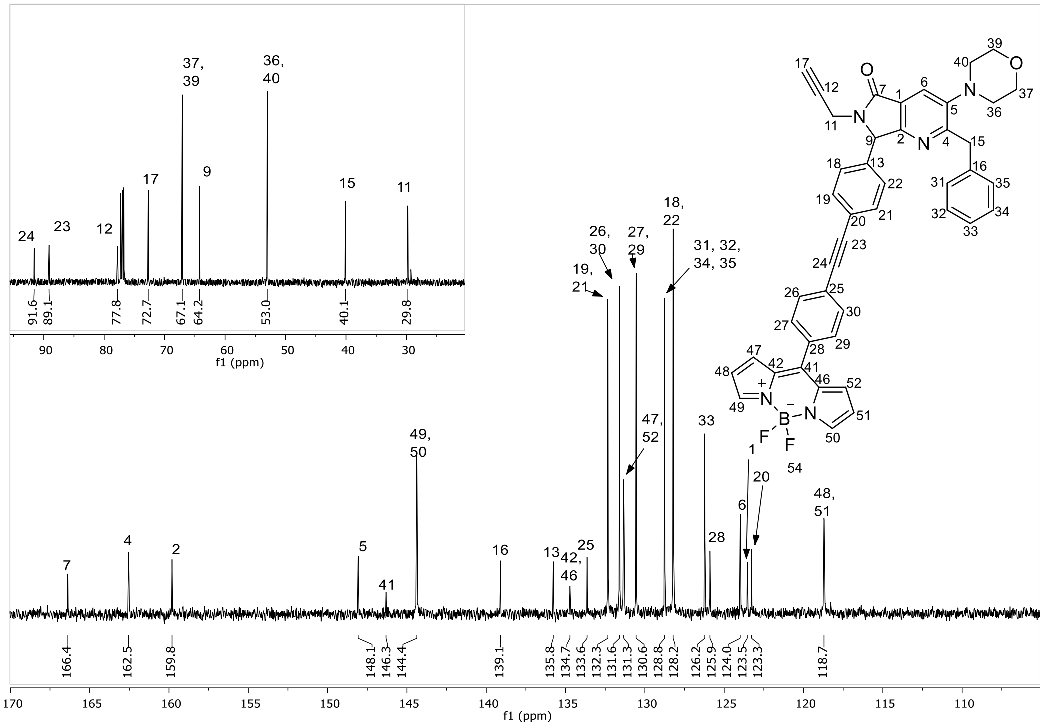

Figure S36. ^{13}C NMR spectrum of compound **11g**

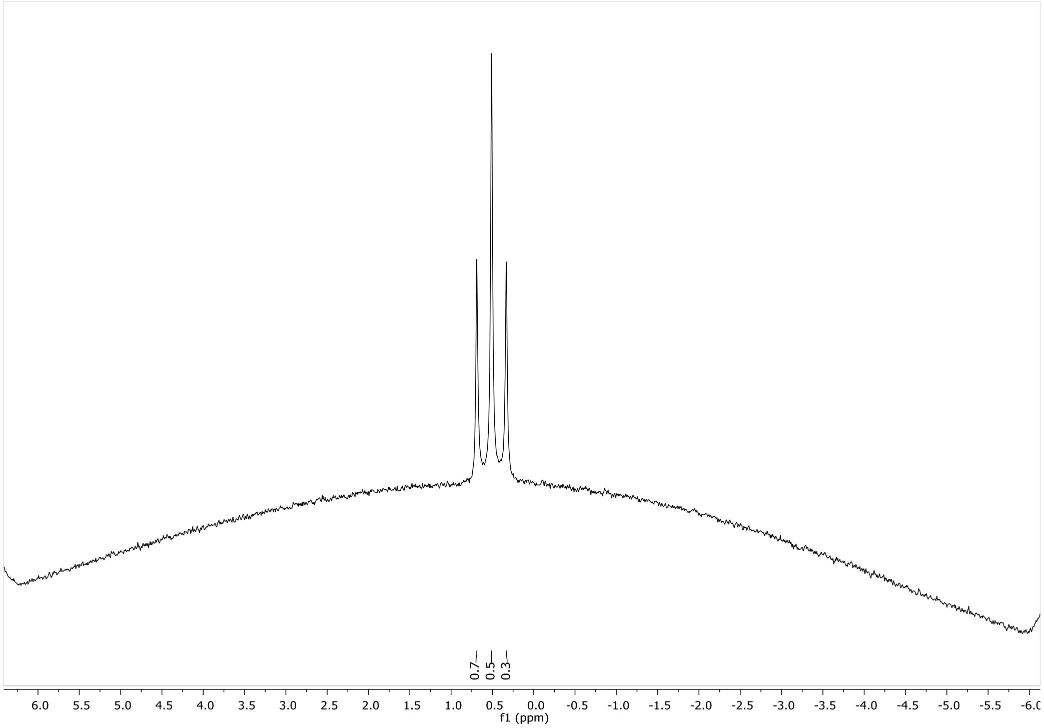

Figure S37. ^{11}B NMR spectrum of compound **11g**

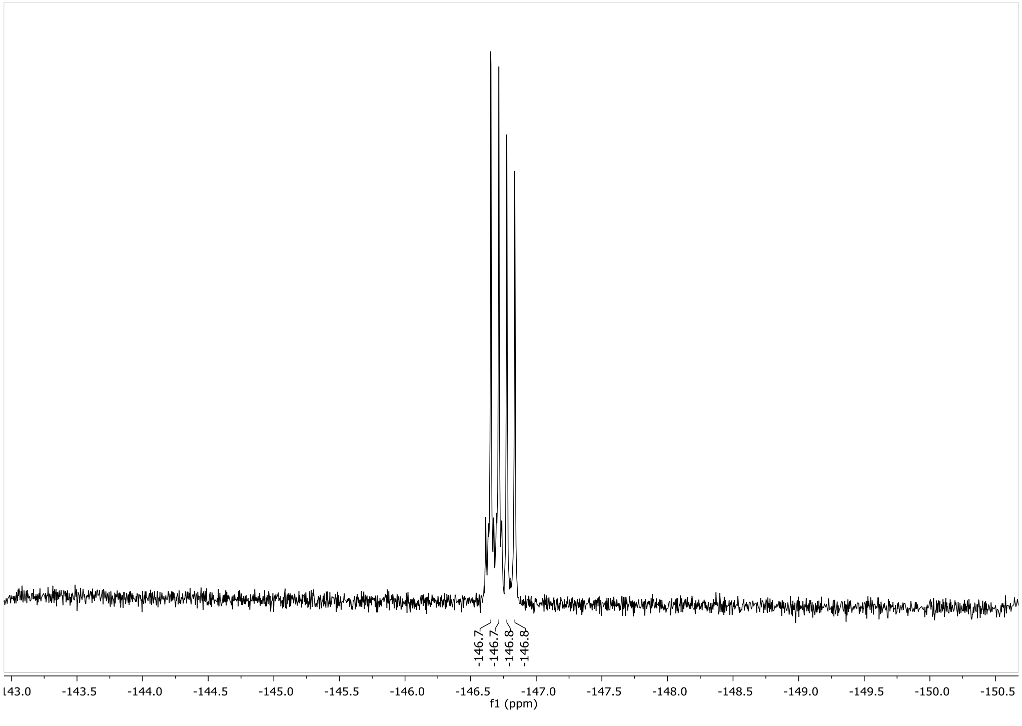

Figure S38. ^{19}F NMR spectrum of compound **11g**

Mass Spectrum SmartFormula Report

Acquisition Parameter

Source Type	ESI	Ion Polarity	Positive	Set Nebulizer	0.5 Bar
Focus	Active			Set Dry Heater	150 °C
Scan Begin	50 m/z	Set Capillary	4500 V	Set Dry Gas	4.0 l/min
Scan End	3000 m/z	Set End Plate Offset	-500 V	Set Divert Valve	Waste

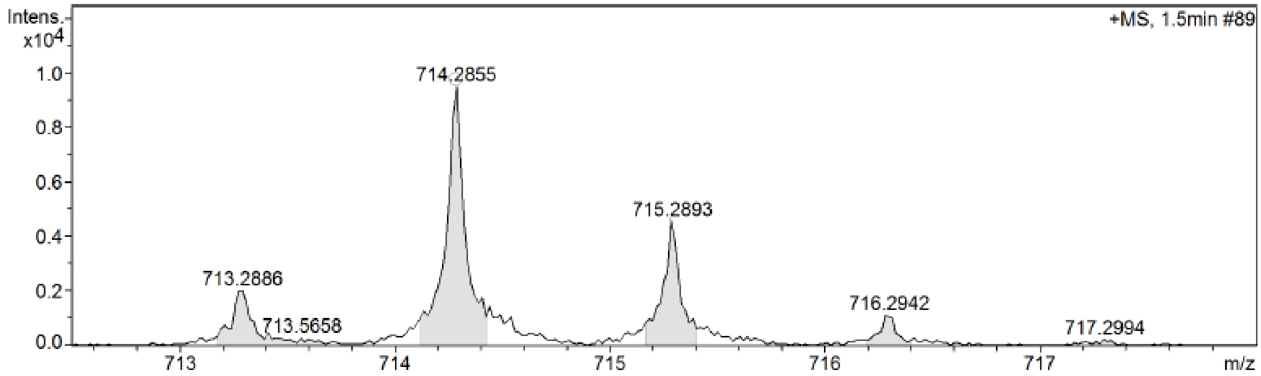

Meas. m/z	#	Ion Formula	Sum Formula	m/z	err [ppm]	|err| [mDa]	mSigma	# mSigma
714.2855	1	C44H35BF2N5O2	C44H34BF2N5O2	714.2846	-0.2	0.1	5.6	1

Figure S39. HRMS spectrum of compound **11g**

3 Emission spectra of compounds **11a-g** with different viscosities

Table S1. Conditions for fluorescence analysis at different viscosities

Name	Condition
0 gly	100% DMSO
1 gly	75 DMSO:25 Glycerol
2 gly	50 DMSO:50 Glycerol
3 gly	25 DMSO:75 Glycerol
4 gly	10 DMSO:90 Glycerol
5 gly	100% Glycerol

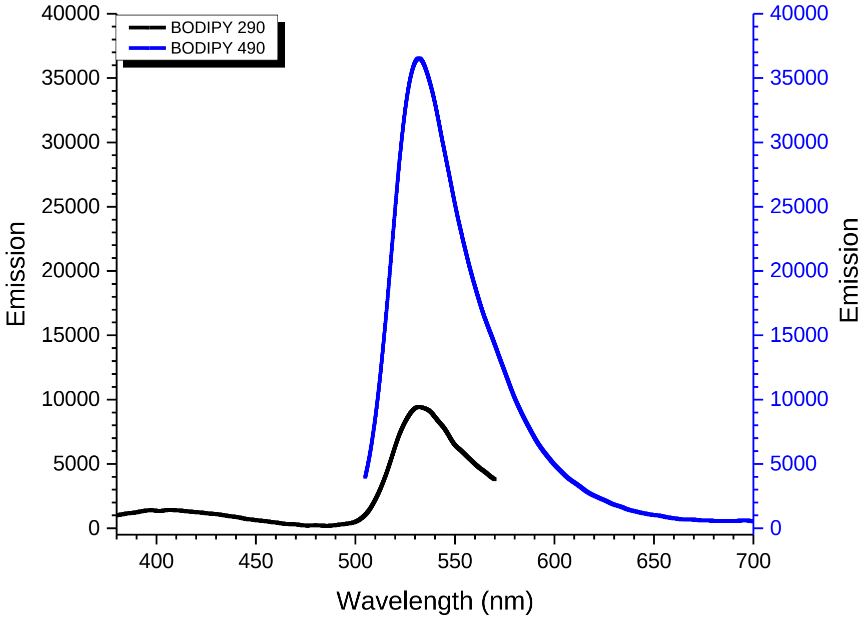

Figure S40. Emission spectra (Excitation at 290 and 490 nm) for compound **7**. DMSO was used as solvent at a concentration of 10^{-6} M at RT.

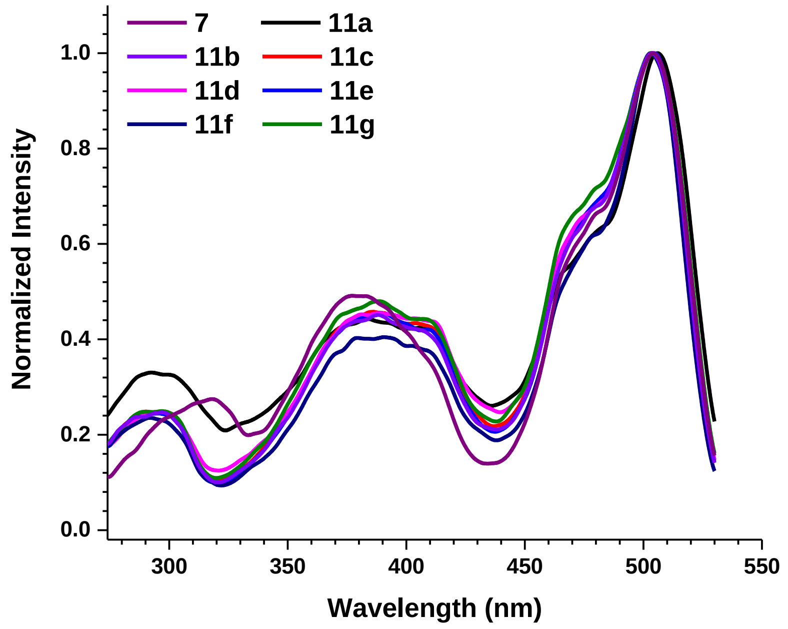

Figure S41. Normalized excitation spectra of compounds **7** and **11a-g** (Emission at 540 nm). DMSO was used as solvent at a concentration of 10^{-6} M at RT.

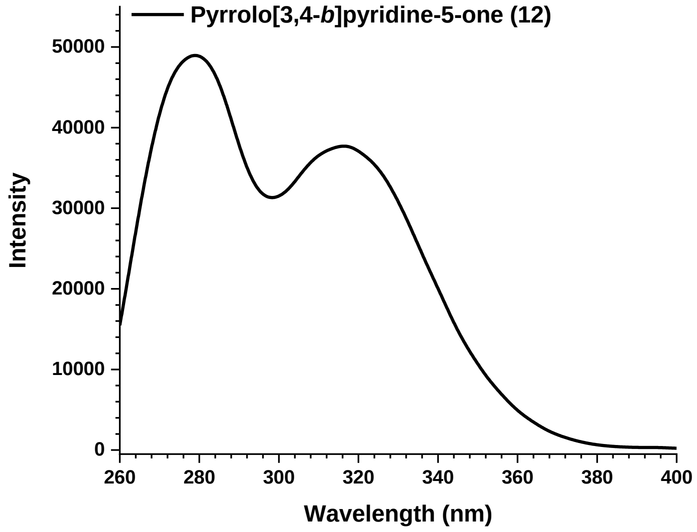

Figure S42. Excitation spectra of compound **12** (emission at 450 nm). DMSO was used as solvent at a concentration of 10−6 M at RT.

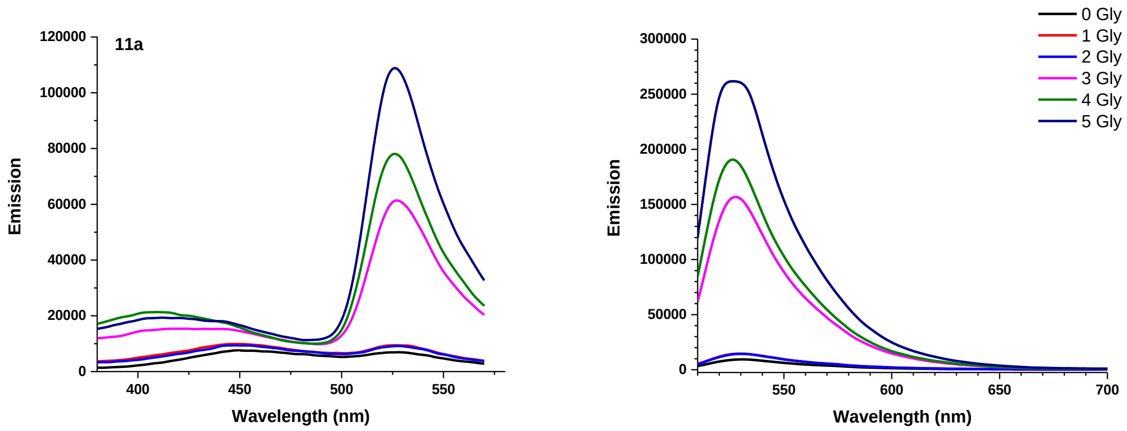

Figure S43. Emission spectra of **11a**. Left: λexc = 290 nm. Right: λexc = 490 nm.

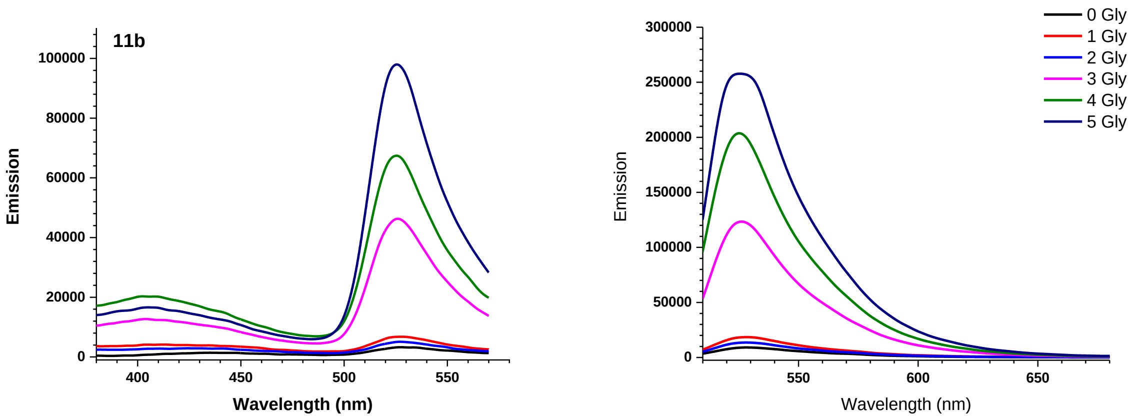

Figure S44. Emission spectra of **11b**. Left: λ_{exc} = 290 nm. Right: λ_{exc} = 490 nm

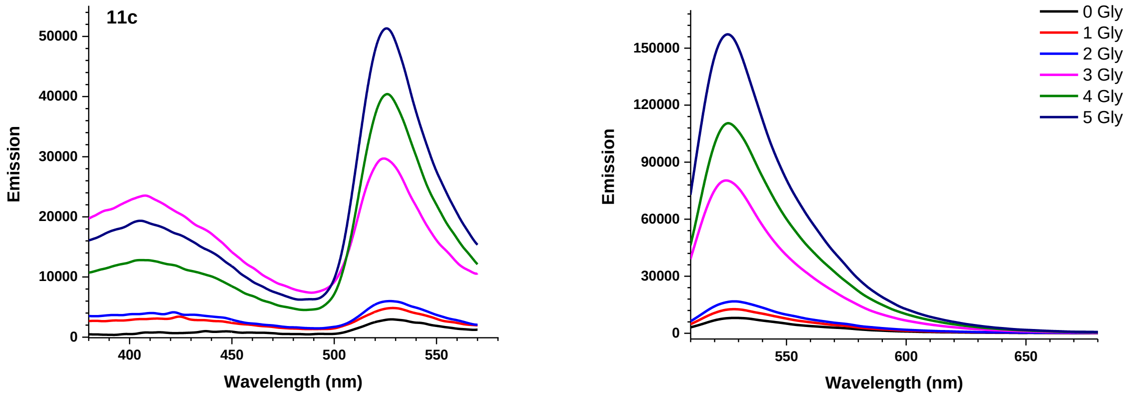

Figure S45. Emission spectra of **11c**. Left: λ_{exc} = 290 nm. Right: λ_{exc} = 490 nm

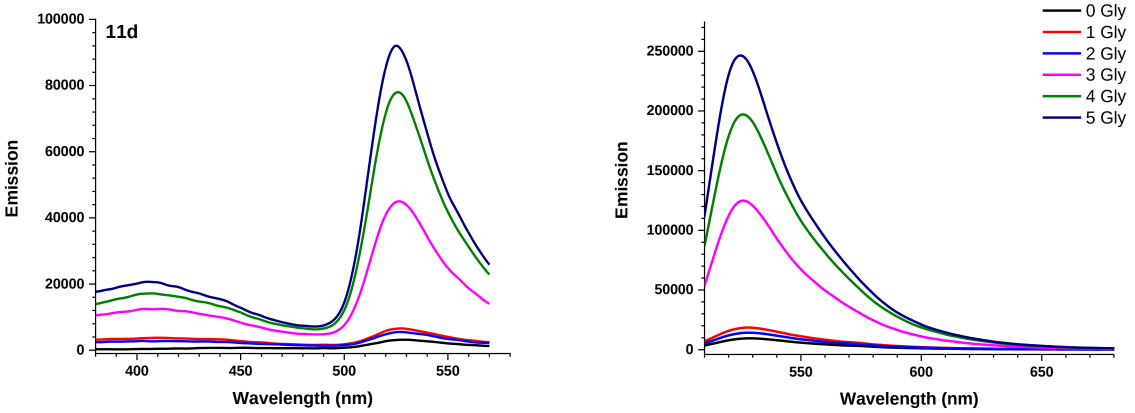

Figure S46. Emission spectra of **11d**. Left: λ_{exc} = 290 nm. Right: λ_{exc} = 490 nm

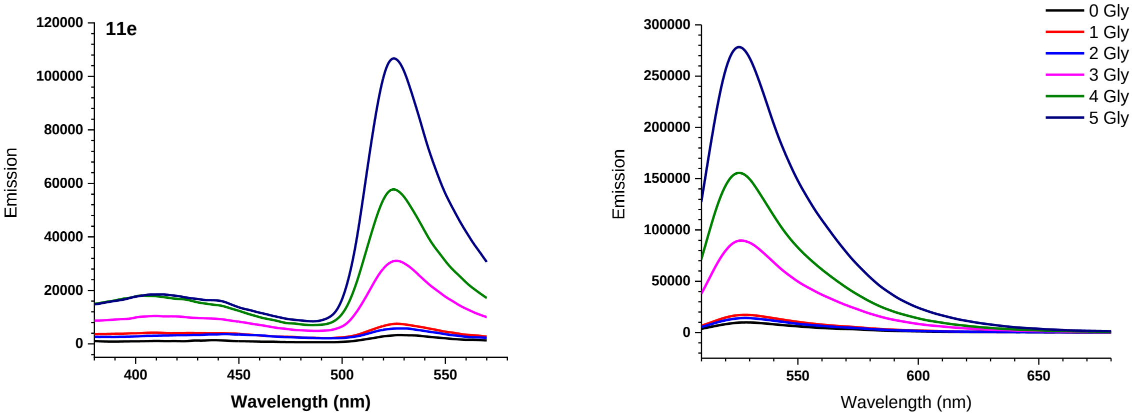

Figure S47. Emission spectra of **11e**. Left: λ_{exc} = 290 nm. Right: λ_{exc} = 490 nm

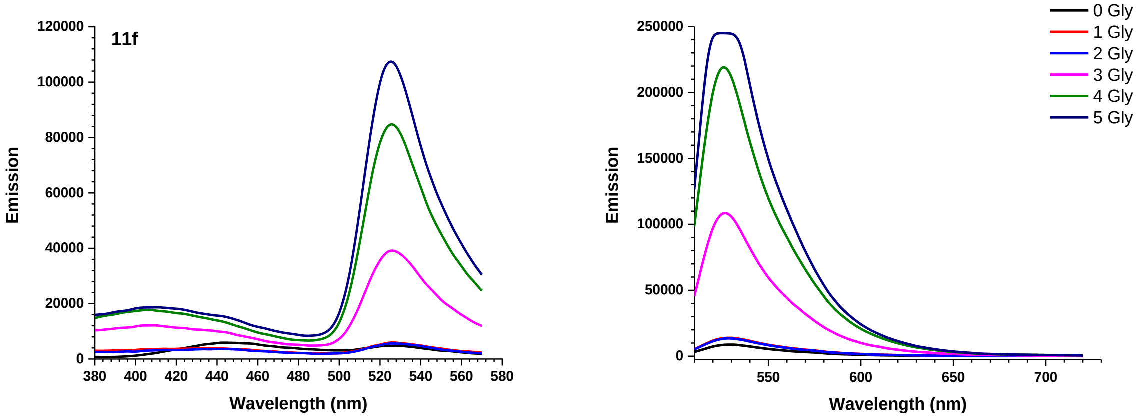

Figure S48. Emission spectra of **11f**. Left: λ_{exc} = 290 nm. Right: λ_{exc} = 490 nm

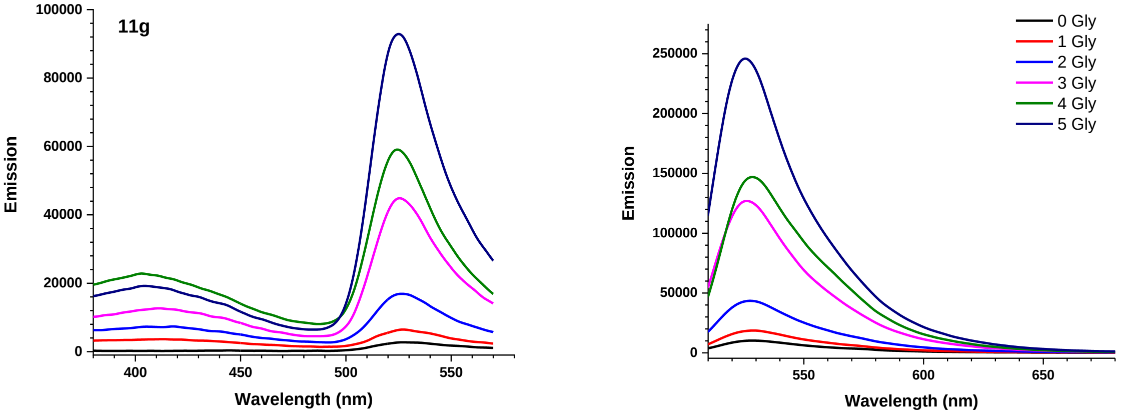

Figure S49. Emission spectra of **11g**. Left: λ_{exc} = 290 nm. Right: λ_{exc} = 490 nm

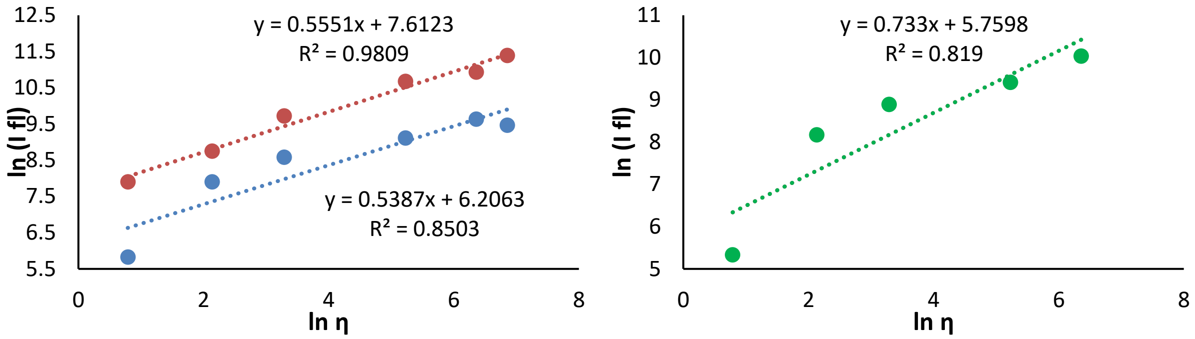

Figure S50. Calibration curves of ln I_f vs ln η for **11g** in DMSO/glycerol mixtures upon excitation at 290 nm and emission at 446 nm (red plot) and 530 nm (blue plot). Emission at 440 nm (due to the formation of a conformer, right).

Table S2. Coefficients χ and R^2 for compounds **11a** to **11g**. Excitation at 490 nm

Compound	χ	R^2
**11a**	0.6081	0.9241
**11b**	0.5854	0.9253
**11c**	0.5027	0.9740
**11d**	0.5668	0.9265
**11e**	0.5561	0.9293
**11f**	0.6082	0.9378
**11g**	0.5110	0.9852

4 Data of DFT and TD-DFT calculations

4.1 Optimized xyz coordinates (B3LYP/6-31G(d), SMD, DMSO) and excitation energies and oscillator strengths (B3LYP/6-311+G(d,p)) of compound **11a**

C	7.495427	-0.7783885	0.113344
C	6.435918	-1.0958741	-0.79229
C	5.705557	1.06498625	-0.79363

C	7.59052	0.53656614	0.591106
H	8.362423	0.8344382	1.292804
C	6.66644	1.46190787	0.124072
C	6.518039	2.91514422	0.380228
C	4.839483	2.25303102	-1.1855
H	4.997334	2.48713892	-2.24786
O	7.217321	3.63839708	1.087169
C	5.12114	4.74080618	-0.5583
H	4.812984	4.87756476	-1.59997
H	6.061531	5.28320627	-0.41351
N	5.431648	3.3233331	-0.36395
C	3.349105	2.03564015	-0.96585
C	2.488272	1.96492866	-2.06635
C	2.817815	1.87659499	0.322098
C	1.124123	1.74621116	-1.89478
H	2.888878	2.0837543	-3.06994
C	1.458552	1.66142067	0.506762
H	3.471049	1.93521968	1.187806
C	0.587951	1.59306173	-0.60218
H	0.465533	1.69663917	-2.75647
H	1.055546	1.54554376	1.508042
N	5.566847	-0.1765461	-1.245
C	6.230638	-2.5191619	-1.2876
H	6.698038	-2.6175318	-2.27718
H	6.772494	-3.1917745	-0.61655
C	4.777881	-2.9455008	-1.37754
C	4.079688	-2.8973233	-2.59041
C	4.108283	-3.4119595	-0.23739
C	2.745532	-3.3027638	-2.66489
H	4.586931	-2.5401875	-3.48359
C	2.774189	-3.8159575	-0.30656
H	4.63863	3.46065444	0.711368
C	2.087897	-3.7633551	-1.52233
H	2.220224	-3.2595487	-3.61586
H	2.271612	-4.1762446	0.587717
H	1.049572	-4.0799041	-1.5784
C	9.391012	-2.2075791	-0.4791
C	9.03367	-1.6242607	1.836322
C	10.09183	-3.48204	-0.01856
H	10.13996	-1.4097151	-0.62552
H	8.900849	-2.3858948	-1.43973
C	9.722196	-2.9284629	2.230303
H	9.788385	-0.8188867	1.825503
H	8.271002	-1.3695451	2.579629
H	10.89885	-3.741154	-0.71077
H	9.371446	-4.3158848	0.008345

H	10.25809	-2.803211	3.17602
H	8.969668	-3.7240754	2.352516
N	8.391915	-1.7984883	0.52765
O	10.69257	-3.31475	1.261765
C	-0.80844	1.3838918	-0.41292
C	-2.00149	1.2094598	-0.24379
C	-3.3962	1.0092531	-0.04268
C	-4.27582	0.90696522	-1.14032
C	-3.92692	0.90831943	1.260041
C	-5.63538	0.70395379	-0.93973
H	-3.88279	0.99269004	-2.14841
C	-5.28919	0.72068458	1.455546
H	-3.26007	0.97437316	2.11392
C	-6.16639	0.61108032	0.360335
H	-6.29942	0.64435405	-1.79609
H	-5.67684	0.62635152	2.464629
C	-7.61634	0.39800922	0.573514
C	-8.33902	1.25322363	1.427795
C	-8.27594	-0.6683757	-0.06783
C	-7.94244	2.41680741	2.138652
H	-6.95112	2.84710622	2.135881
C	-9.06318	2.89283179	2.80955
H	-9.12724	3.76212207	3.449993
C	-10.1249	7,2.027100	2.489177
H	-11.1597	2.071485	2.803349
N	-9.69947	1.0520631	1.666961
N	-9.64243	-0.885673	0.116138
C	-7.78036	-1.709728	-0.89656
H	-6.7511	-1.8334048	-1.20198
C	-8.85506	-2.533062	-1.21227
H	-8.84395	-3.424546	-1.82464
C	-9.98188	-1.994323	-0.56457
H	-11.0016	-2.35701	-0.55478
B	-10.5952	-0.0393	1.010359
F	-11.1745	-0.84537	1.986619
F	-11.5849	0.559967	0.234548
C	4.060675	5.30598489	0.372648
C	4.298533	5.38630819	1.753204
C	2.844962	5.77931001	-0.13275
C	3.333803	5.92093914	2.607193
H	5.245791	5.03105421	2.150513
C	1.878727	6.3187895	0.720688
H	2.650263	5.72213284	0.201415
C	2.120337	6.38885221	2.093271
H	3.530814	5.9784026	3.674838

H	0.93848	6.67989395	0.311804
H	1.370145	6.80715265	2.759451

Excited State 1: Singlet-A 2.5567 eV 484.94 nm f=0.3588 <S**2>=0.000

199 -> 201 0.43935

200 -> 201 0.54099

Excited State 23: Singlet-A 4.3808 eV 283.02 nm f=0.1057 <S**2>=0.000

191 -> 203 -0.11245

200 -> 204 0.64273

4.2 Optimized xyz coordinates (B3LYP/6-31G(d), SMD, DMSO) and excitation energies and oscillator strengths (B3LYP/6-311+G(d,p)) of compound **11b**

C	7.334261	-0.703473	0.124791
C	6.281727	-1.055301	-0.775970
C	5.506555	1.089976	-0.806765
C	7.401381	0.619781	0.584907
H	8.166326	0.942974	1.283156
C	6.457539	1.518987	0.107196
C	6.269910	2.970942	0.353233
C	4.616491	2.254812	-1.213367
H	4.778070	2.484415	-2.276369
O	6.950759	3.719947	1.051143
C	4.787049	4.744311	-0.543197
H	4.203214	4.826982	-1.465897
H	5.704080	5.331174	-0.677475
N	5.172381	3.342388	-0.389852
C	3.129168	2.009345	-1.005630
C	2.271898	1.946126	-2.109139
C	2.595897	1.822604	0.278372
C	0.910446	1.705046	-1.945023
H	2.673939	2.086841	-3.109320
C	1.238791	1.586034	0.455968
H	3.248995	1.868674	1.145301
C	0.372261	1.524187	-0.656825
H	0.255293	1.659663	-2.809518
H	0.834998	1.445471	1.453880
N	5.394966	-0.159994	-1.242674
C	6.101388	-2.490112	-1.247232
H	6.565807	-2.597308	-2.237236
H	6.657717	-3.141579	-0.567113

C	4.656162	-2.944196	-1.322619
C	3.960417	-2.957523	-2.537714
C	3.991551	-3.375696	-0.165726
C	2.633754	-3.389328	-2.598394
H	4.463853	-2.628184	-3.443686
C	2.665000	-3.805507	-0.221096
H	4.520077	-3.376694	0.785326
C	1.981142	-3.814541	-1.439454
H	2.110464	-3.394099	-3.551442
H	2.166321	-4.137984	0.686064
H	0.948710	-4.151449	-1.484670
C	9.252357	-2.114221	-0.444581
C	8.892780	-1.487900	1.858568
C	9.973458	-3.368138	0.040457
H	9.989176	-1.308691	-0.609377
H	8.763243	-2.319888	-1.400187
C	9.603484	-2.772273	2.278089
H	9.634187	-0.670723	1.827663
H	8.128603	-1.229653	2.599113
H	10.781711	-3.630954	-0.648961
H	9.265197	-4.211391	0.088539
H	10.140701	-2.617859	3.218764
H	8.864135	-3.576855	2.420731
N	8.249731	-1.699538	0.556122
O	10.576280	-3.164484	1.314509
C	-1.021784	1.291144	-0.477832
C	-2.213710	1.096923	-0.323338
C	-3.607897	0.872777	-0.144256
C	-4.461616	0.723446	-1.256879
C	-4.163535	0.794190	1.149466
C	-5.820677	0.496985	-1.079182
H	-4.048984	0.790614	-2.258514
C	-5.525736	0.584045	1.321658
H	-3.516396	0.896400	2.014893
C	-6.376786	0.427647	0.211683
H	-6.465120	0.399842	-1.947069
H	-5.933297	0.509160	2.324538
C	-7.826698	0.191679	0.401011
C	-8.584341	1.061291	1.208802
C	-8.450590	-0.909235	-0.216729
C	-8.226564	2.258467	1.883524
H	-7.244045	2.708585	1.885403
C	-9.370976	2.735829	2.512052
H	-9.466533	3.626957	3.117614
C	-10.407920	1.836685	2.202428

H	-11.449903	1.871013	2.493365
N	-9.945302	0.840744	1.426744
N	-9.815847	-1.149337	-0.052398
C	-7.917030	-1.965799	-1.001396
H	-6.879603	-2.077007	-1.283049
C	-8.968017	-2.821306	-1.311179
H	-8.926386	-3.731165	-1.894534
C	-10.118567	-2.286082	-0.703362
H	-11.130532	-2.669956	-0.701863
B	-10.802973	-0.296662	0.797825
F	-11.375135	-1.082235	1.794883
F	-11.796606	0.248032	-0.012307
C	3.991256	5.305881	0.643051
H	4.585660	5.173927	1.555608
H	3.069841	4.723900	0.772076
C	3.648148	6.789369	0.459933
H	3.059481	6.918318	-0.459850
H	4.575667	7.360966	0.313712
C	2.874144	7.372122	1.645751
H	3.450934	7.287334	2.575444
H	2.646871	8.433762	1.491100
H	1.922339	6.847305	1.797140

Excited State 1: Singlet-A 2.5563 eV 485.02 nm f=0.3267 <S**2>=0.000

190 -> 193 0.27423

191 -> 193 -0.32377

192 -> 193 0.56097

Excited State 21: Singlet-A 4.3900 eV 282.42 nm f=0.1075 <S**2>=0.000

186 -> 195 0.12062

187 -> 195 0.10403

192 -> 196 0.63667

4.3 Optimized xyz coordinates (B3LYP/6-31G(d), SMD, DMSO) and excitation energies and oscillator strengths (B3LYP/6-311+G(d,p)) of compound **11c**

C	7.2408698	-0.6818431	0.1217243
C	6.1807211	-1.0209079	-0.7757662
C	5.3983404	1.1222148	-0.7598712
C	7.3043753	0.6318010	0.6092095
H	8.0725114	0.9438771	1.3088967

C	6.3528064	1.5365005	0.1570933
C	6.1574214	2.9810181	0.4374037
C	4.4973183	2.2889503	-1.1355569
H	4.6459163	2.5416738	-2.1950025
O	6.8314852	3.7192334	1.1527584
C	4.6590365	4.7620533	-0.4118210
H	4.0888578	4.8742633	-1.3390078
H	5.5675751	5.3678325	-0.5024238
N	5.0563588	3.3630321	-0.2964100
C	3.0142519	2.0292538	-0.9157238
C	2.1441667	1.9832839	-2.0099763
C	2.4973724	1.8119773	0.3703169
C	0.7869246	1.7265701	-1.8350323
H	2.5328446	2.1470060	-3.0118426
C	1.1441604	1.5613329	0.5588944
H	3.1606664	1.8421932	1.2302546
C	0.2650704	1.5151513	-0.5446505
H	0.1221504	1.6934575	-2.6926562
H	0.7534356	1.3967065	1.5583141
N	5.2880695	-0.1197143	-1.2183436
C	6.0016625	-2.4484675	-1.2698284
H	6.4986077	-2.5498973	-2.2447306
H	6.5311600	-3.1126839	-0.5799884
C	4.5565190	-2.8895021	-1.4006867
C	3.8954713	-2.8537998	-2.6345176
C	3.8558299	-3.3543102	-0.2788441
C	2.5675324	-3.2710808	-2.7472979
H	4.4261766	-2.4971639	-3.5141826
C	2.5279603	-3.7707230	-0.3862822
H	4.3568502	-3.3917820	0.6861730
C	1.8788093	-3.7302978	-1.6226876
H	2.0716426	-3.2375517	-3.7142722
H	2.0014898	-4.1297169	0.4946442
H	0.8455187	-4.0565543	-1.7088788
C	9.1488685	-2.0828234	-0.5010263
C	8.8342395	-1.4809334	1.8157587
C	9.8839054	-3.3386545	-0.0435014
H	9.8790589	-1.2730100	-0.6732523
H	8.6416582	-2.2813776	-1.4484155
C	9.5590912	-2.7663270	2.2064953
H	9.5708299	-0.6594518	1.7791251
H	8.0844661	-1.2352962	2.5751001
H	10.6778968	-3.5922641	-0.7526750
H	9.1793974	-4.1845814	0.0116119
H	10.1153307	-2.6193747	3.1373184

H	8.8268475	-3.5759828	2.3571882
N	8.1656754	-1.6802939	0.5241249
O	10.5131663	-3.1449412	1.2190781
C	-1.1244529	1.2612693	-0.3584153
C	-2.3128972	1.0451611	-0.2064376
C	-3.7029796	0.7895453	-0.0367702
C	-4.5323506	0.5611422	-1.1544637
C	-4.2781663	0.7550460	1.2502025
C	-5.8862348	0.2985129	-0.9878454
H	-4.1046228	0.5938989	-2.1514752
C	-5.6357955	0.5084960	1.4105234
H	-3.6499081	0.9192550	2.1199877
C	-6.4619225	0.2710633	0.2959741
H	-6.5119261	0.1394419	-1.8603093
H	-6.0582184	0.4675298	2.4092709
C	-7.9053954	-0.0083614	0.4744755
C	-8.7066724	0.8692933	1.2296264
C	-8.4797249	-1.1573329	-0.1023667
C	-8.4049441	2.1125536	1.8460042
H	-7.4404377	2.6000674	1.8373040
C	-9.5763155	2.5772059	2.4330296
H	-9.7151210	3.4944472	2.9892036
C	-10.5736303	1.6241006	2.1563598
H	-11.6204857	1.6328268	2.4312099
N	-10.0616699	0.6082437	1.4397878
N	-9.8380456	-1.4391967	0.0529306
C	-7.8981712	-2.2234206	-0.8385106
H	-6.8535971	-2.3078979	-1.1025246
C	-8.9142824	-3.1260480	-1.1303427
H	-8.8327037	-4.0545301	-1.6790969
C	-10.0915691	-2.6092315	-0.5589055
H	-11.0894434	-3.0283418	-0.5559506
B	-10.8636122	-0.5982266	0.8688458
F	-11.3915139	-1.3647780	1.9047333
F	-11.8864347	-0.1422581	0.0409793
C	3.8276844	5.2668983	0.7879127
H	4.4192221	5.1177813	1.6975139
H	2.9246732	4.6534079	0.8761983
C	3.4548493	6.7264008	0.6409213
C	4.2973814	7.7368092	1.1278110
C	2.2740383	7.1031156	-0.0161406
C	3.9695520	9.0844030	0.9650767
H	5.2149230	7.4627665	1.6444230
C	1.9424185	8.4493621	-0.1813394
H	1.6047696	6.3333762	-0.3953869
C	2.7901552	9.4453750	0.3092186

H	4.6333251	9.8521099	1.3550381
H	1.0196678	8.7196302	-0.6888852
H	2.5317571	10.4938543	0.1850032

Excited State 1: Singlet-A 2.5584 eV 484.62 nm f=0.3272 <S**2>=0.000

202 -> 205 -0.41864

204 -> 205 0.56358

Excited State 23: Singlet-A 4.3871 eV 282.61 nm f=0.1146 <S**2>=0.000

196 -> 207 -0.11071

201 -> 206 0.17806

204 -> 208 0.61891

4.4 Optimized xyz coordinates (B3LYP/6-31G(d), SMD, DMSO) and excitation energies and oscillator strengths (B3LYP/6-311+G(d,p)) of compound **11d**

C	7.803195	-0.913371	-0.042841
C	6.712147	-1.213336	-0.915689
C	6.049809	0.969623	-0.939439
C	7.956155	0.407520	0.401787
H	8.757892	0.692530	1.074767
C	7.050606	1.353587	-0.059470
C	6.958098	2.816180	0.168357
C	5.207680	2.176894	-1.326249
H	5.332153	2.383789	-2.398572
O	7.704400	3.530021	0.836589
C	5.587975	4.669465	-0.753477
H	5.220185	4.795517	-1.776645
H	6.554173	5.180121	-0.680863
N	5.863594	3.244443	-0.550214
C	3.720650	2.012235	-1.044238
C	2.814432	1.935053	-2.107146
C	3.236618	1.912830	0.268037
C	1.451588	1.769938	-1.874946
H	3.177614	2.007930	-3.129168
C	1.879527	1.751755	0.512881
H	3.925630	1.977200	1.105084
C	0.962880	1.679776	-0.557946
H	0.757340	1.717212	-2.707934
H	1.512906	1.683870	1.532367
N	5.860086	-0.275194	-1.362546

C	6.453235	-2.638072	-1.381395
H	6.886235	-2.763186	-2.383526
H	6.998302	-3.314564	-0.717163
C	4.988022	-3.028623	-1.418490
C	4.246223	-2.959095	-2.604268
C	4.350208	-3.481485	-0.254928
C	2.900384	-3.330520	-2.629063
H	4.728364	-2.612135	-3.515137
C	3.004591	-3.851527	-0.274494
H	4.914677	-3.546252	0.672963
C	2.274620	-3.777511	-1.463337
H	2.340751	-3.271245	-3.559355
H	2.527023	-4.201600	0.637376
H	1.227175	-4.067355	-1.480912
C	9.645049	-2.409760	-0.638377
C	9.340424	-1.775337	1.671757
C	10.316631	-3.695374	-0.165087
H	10.413585	-1.636305	-0.812242
H	9.133426	-2.592063	-1.587244
C	9.995122	-3.092427	2.078801
H	10.118403	-0.993151	1.636335
H	8.597036	-1.485024	2.421511
H	11.106367	-3.988485	-0.863623
H	9.573678	-4.508005	-0.114145
H	10.547850	-2.968044	3.014922
H	9.220933	-3.862875	2.224778
N	8.674328	-1.953978	0.375857
O	10.939944	-3.523946	1.103901
C	-0.430895	1.530500	-0.304556
C	-1.620537	1.409796	-0.075365
C	-3.009839	1.278136	0.203454
C	-3.952950	1.196583	-0.841853
C	-3.472096	1.229576	1.535016
C	-5.307469	1.065068	-0.562971
H	-3.612918	1.243868	-1.871538
C	-4.828628	1.112101	1.808557
H	-2.756682	1.280863	2.349683
C	-5.770068	1.024590	0.765765
H	-6.019763	1.023376	-1.380645
H	-5.162977	1.055725	2.839260
C	-7.214455	0.888850	1.061722
C	-7.843986	1.788151	1.944446
C	-7.962930	-0.148571	0.471666
C	-7.349338	2.934853	2.620550
H	-6.339414	3.314259	2.557730
C	-8.404765	3.472350	3.348598

H	-8.388050	4.348350	3.982835
C	-9.525688	2.660730	3.096004
H	-10.537862	2.761095	3.466107
N	-9.197494	1.659381	2.260873
N	-9.326916	-0.291043	0.731207
C	-7.568009	-1.224591	-0.366795
H	-6.564440	-1.406722	-0.724185
C	-8.699765	-1.993720	-0.612469
H	-8.768066	-2.892348	-1.210537
C	-9.760184	-1.388378	0.086214
H	-10.795418	-1.696270	0.156155
B	-10.185439	0.617641	1.658538
F	-10.766014	-0.144071	2.669063
F	-11.173082	1.268374	0.922095
C	4.607229	5.288278	0.227890
C	4.933433	5.420672	1.580845
C	3.363599	5.766497	-0.204864
C	4.035394	5.993458	2.484973
H	5.898104	5.069063	1.935927
C	2.458160	6.344129	0.681090
H	3.068167	5.684169	-1.247741
C	2.787674	6.454414	2.050152
H	4.314351	6.074548	3.529570
O	1.223104	6.731851	0.212867
O	1.835830	7.005831	2.856750
C	0.995227	8.145212	0.166975
H	1.041876	8.595440	1.163916
H	1.722294	8.638833	-0.491901
H	-0.008908	8.277591	-0.244355
C	2.111613	7.106818	4.251013
H	1.217175	7.544224	4.699371
H	2.294344	6.121341	4.697275
H	2.970277	7.761513	4.446640

Excited State 1: Singlet-A 2.5363 eV 488.84 nm f=0.3257 <S**2>=0.000

213 -> 217 0.36238

215 -> 217 0.21873

216 -> 217 0.55882

Excited State 26: Singlet-A 4.3779 eV 283.21 nm f=0.1009 <S**2>=0.000

215 -> 220 -0.15817

216 -> 220 0.63291

4.5 Optimized xyz coordinates (B3LYP/6-31G(d), SMD, DMSO) and excitation energies and oscillator strengths (B3LYP/6-311+G(d,p)) of compound **11e**

C	7.500548	-0.766069	0.103763
C	6.458909	-1.096882	-0.817364
C	5.706084	1.056554	-0.835966
C	7.578594	0.552238	0.574611
H	8.339236	0.859278	1.284525
C	6.652901	1.467061	0.090615
C	6.488386	2.919719	0.337802
C	4.830649	2.233785	-1.240830
H	4.992469	2.464009	-2.303324
O	7.171996	3.653687	1.049433
C	5.080997	4.727989	-0.614747
H	4.757536	4.859259	-1.652148
H	6.017787	5.280292	-0.484817
N	5.407103	3.314177	-0.420347
C	3.340694	2.006561	-1.026117
C	2.480229	1.937167	-2.126948
C	2.808989	1.844083	0.261305
C	1.115731	1.718283	-1.956001
H	2.880820	2.057861	-3.130317
C	1.449829	1.628746	0.445331
H	3.461767	1.899904	1.127554
C	0.579047	1.564133	-0.663781
H	0.457341	1.670838	-2.817959
H	1.046464	1.510534	1.446196
N	5.586695	-0.187878	-1.284962
C	6.278551	-2.523446	-1.313341
H	6.754399	-2.614184	-2.299592
H	6.825722	-3.187323	-0.638256
C	4.833540	-2.973885	-1.412870
C	4.135858	-2.918262	-2.625831
C	4.170916	-3.469422	-0.280972
C	2.808811	-3.344969	-2.708032
H	4.637901	-2.538772	-3.512671
C	2.843842	-3.894777	-0.357940
H	4.701448	-3.524171	0.667359
C	2.157861	-3.834152	-1.573457
H	2.283548	-3.295948	-3.658740
H	2.346625	-4.277576	0.529957
H	1.125075	-4.167200	-1.635776
C	9.424393	-2.176692	-0.440008
C	9.005437	-1.589913	1.865061
C	10.129479	-3.441000	0.041825

H	10.166618	-1.370213	-0.573127
H	8.957614	-2.365190	-1.410512
C	9.697761	-2.885343	2.279361
H	9.751179	-0.776213	1.870555
H	8.222430	-1.341610	2.589127
H	10.956702	-3.691456	-0.629375
H	9.419068	-4.283692	0.053000
H	10.208611	-2.752677	3.237840
H	8.950936	-3.688710	2.384706
N	8.397561	-1.775773	0.541773
O	10.696170	-3.263314	1.336183
C	-0.817464	1.358912	-0.471256
C	-2.009610	1.189706	-0.290942
C	-3.402256	0.998812	-0.067565
C	-4.301858	0.900778	-1.149058
C	-3.910765	0.905727	1.244587
C	-5.659373	0.710042	-0.923728
H	-3.926261	0.981070	-2.164251
C	-5.270676	0.729196	1.464684
H	-3.228579	0.968783	2.086485
C	-6.168428	0.624871	0.385677
H	-6.338758	0.655382	-1.768201
H	-5.640380	0.639519	2.480848
C	-7.616308	0.425515	0.624377
C	-8.314906	1.283485	1.495906
C	-8.298217	-0.631586	-0.009463
C	-7.893232	2.436644	2.209574
H	-6.897719	2.856785	2.192773
C	-8.996478	2.918395	2.904839
H	-9.039471	3.782575	3.553875
C	-10.073133	2.067173	2.595789
H	-11.101822	2.120304	2.928244
N	-9.673122	1.095059	1.757549
N	-9.663909	-0.833971	0.195476
C	-7.827452	-1.676932	-0.847659
H	-6.804382	-1.811988	-1.168842
C	-8.916080	-2.487889	-1.147422
H	-8.924342	-3.379057	-1.760305
C	-10.026436	-1.937751	-0.481256
H	-11.049879	-2.288906	-0.455651
B	-10.594382	0.025795	1.099956
F	-11.184956	-0.773851	2.074511
F	-11.575860	0.649881	0.332914
C	4.027221	5.284972	0.328111
C	4.282274	5.372397	1.705628

C	2.798499	5.742967	-0.160312
C	3.329090	5.894838	2.578334
H	5.237412	5.029524	2.093459
C	1.832474	6.274081	0.697631
H	2.586213	5.682762	-1.224832
C	2.118988	6.336514	2.054203
H	3.513487	5.967937	3.645558
H	0.875653	6.629559	0.328444
F	1.189169	6.846703	2.898660

Excited State 1: Singlet-A 2.5590 eV 484.50 nm f=0.3611 <S**2>=0.000

202 -> 205 0.18847

203 -> 205 0.40976

204 -> 205 0.53977

Excited State 23: Singlet-A 4.3762 eV 283.32 nm f=0.1042 <S**2>=0.000

196 -> 207 -0.10655

200 -> 207 -0.10175

204 -> 208 0.64478

4.6 Optimized xyz coordinates (B3LYP/6-31G(d), SMD, DMSO) and excitation energies and oscillator strengths (B3LYP/6-311+G(d,p)) of compound **11f**

C	12.7719544	7.4326495	1.8851113
C	12.3800306	6.4278833	2.8236321
C	10.1792574	6.6940604	2.2841716
C	11.7681003	8.1019918	1.1701311
H	12.0024197	8.8854315	0.4574245
C	10.4515931	7.7209879	1.3925779
C	9.1787034	8.1842255	0.7860270
C	8.6814143	6.4261530	2.3238472
H	8.4796574	5.4258620	1.9149223
O	9.0132350	9.0324664	-0.0882526
C	6.8026000	7.4910586	0.9123342
H	6.2981004	6.5879404	1.2681288
H	6.8099792	7.4589888	-0.1832666
N	8.1830223	7.4452386	1.3848881
C	8.0667067	6.5001221	3.7139269
C	7.5999916	5.3346270	4.3312450
C	7.9813391	7.7153223	4.4094505
C	7.0599641	5.3709140	5.6138402

H	7.6626306	4.3861809	3.8039697
C	7.4381390	7.7659132	5.6872090
H	8.3415056	8.6289717	3.9450795
C	6.9678368	6.5905746	6.3113494
H	6.7022882	4.4590722	6.0820193
H	7.3754919	8.7117666	6.2164710
N	11.1002363	6.0578813	2.9995360
C	13.4124274	5.7272433	3.6938482
H	13.6909048	4.7770876	3.2172008
H	14.3155631	6.3448653	3.7086765
C	12.9537065	5.4548943	5.1135005
C	12.4679539	4.1969850	5.4905144
C	13.0193497	6.4657108	6.0828121
C	12.0581605	3.9521497	6.8030287
H	12.4119236	3.4026227	4.7498580
C	12.6088638	6.2263973	7.3951091
H	13.3975696	7.4475597	5.8060491
C	12.1266907	4.9668472	7.7598440
H	11.6862056	2.9680829	7.0775562
H	12.6689537	7.0220999	8.1335690
H	11.8099478	4.7775572	8.7823131
C	14.9484148	6.7478749	1.0009504
C	14.4587375	9.1000243	1.2396126
C	16.4319103	7.0739226	1.1414340
H	14.6741249	6.7380931	-0.0682792
H	14.7548777	5.7507108	1.4040756
C	15.9552399	9.3549077	1.3982220
H	14.1919280	9.2275031	0.1760185
H	13.8952655	9.8344825	1.8244762
H	17.0288013	6.3913353	0.5286875
H	16.7407901	6.9613540	2.1935738
H	16.2189419	10.3325482	0.9832723
H	16.2246493	9.3408775	2.4666783
N	14.1463400	7.7514383	1.7289026
O	16.7238352	8.3907340	0.6852559
C	6.4091051	6.6360420	7.6212047
C	5.9315464	6.6703200	8.7405954
C	5.3763818	6.7042853	10.0510288
C	5.0850419	5.5079814	10.7389227
C	5.1086314	7.9331966	10.6885925
C	4.5548284	5.5421778	12.0224371
H	5.2767834	4.5549956	10.2560535
C	4.5643936	7.9619782	11.9663271
H	5.3371915	8.8617149	10.1749590
C	4.2824634	6.7683948	12.6570569

H	4.3221344	4.6117754	12.5304065
H	4.3844472	8.9164492	12.4502530
C	3.7198178	6.8053062	14.0262439
C	2.5664898	7.5669509	14.2960278
C	4.3405416	6.0854355	15.0652363
C	1.7161135	8.3109272	13.4356837
H	1.8451538	8.4228153	12.3685116
C	0.6956698	8.8382034	14.2188468
H	-0.1296009	9.4578416	13.8950142
C	0.9278445	8.3996785	15.5353708
H	0.3473444	8.5876156	16.4293580
N	2.0376749	7.6424995	15.5855664
N	3.8243015	6.1030938	16.3620054
C	5.5296188	5.3090223	15.0837472
H	6.1733124	5.1252849	14.2352151
C	5.7069909	4.8565931	16.3861360
H	6.5088586	4.2372189	16.7646158
C	4.6386621	5.3719483	17.1428911
H	4.4323369	5.2561200	18.1990205
B	2.5808642	6.9060734	16.8454443
F	2.9576082	7.8254117	17.8212471
F	1.6105615	6.0433953	17.3489134
C	6.0359977	8.7480605	1.3785421
H	6.5866822	9.6280584	1.0297966
H	6.0299406	8.7750564	2.4733948
C	4.6189722	8.7748748	0.8457654
C	4.3467132	9.2344185	-0.4463559
C	3.5586609	8.2956367	1.6328913
C	3.0375657	9.2273187	-0.9356891
H	5.1543508	9.6094511	-1.0700038
C	2.2479289	8.2756799	1.1466736
H	3.7695603	7.9363265	2.6347011
C	1.9855654	8.7559157	-0.1563997
H	2.8119793	9.5862736	-1.9361091
O	1.1690746	7.8114490	1.8414738
O	0.7174227	8.7095199	-0.6912853
C	-0.2127993	9.6520543	-0.1455832
H	-0.3956359	9.4695138	0.9185353
H	0.1448855	10.6810951	-0.2842849
H	-1.1446645	9.5177184	-0.7012922
C	1.3804174	7.2986661	3.1536534
H	2.0544674	6.4329360	3.1429370
H	1.7830281	8.0654832	3.8273558
H	0.3976337	6.9846463	3.5113021

Excited State 1: Singlet-A 2.5596 eV 484.38 nm f=0.3350 <S**2>=0.000

217 -> 221 -0.41983

220 -> 221 0.56011

Excited State 26: Singlet-A 4.3933 eV 282.21 nm f=0.1116 <S**2>=0.000

212 -> 223 0.12510

215 -> 223 -0.10284

219 -> 224 -0.12709

220 -> 224 0.61658

4.7 Optimized xyz coordinates (B3LYP/6-31G(d), SMD, DMSO) and excitation energies and oscillator strengths (B3LYP/6-311+G(d,p)) of compound **11g**

C	7.2511275	-0.6733840	0.2084868
C	6.2153748	-0.9746402	-0.7303210
C	5.4292364	1.1652430	-0.6460098
C	7.3031927	0.6198790	0.7480778
H	8.0539148	0.9040845	1.4778177
C	6.3626084	1.5419180	0.3081089
C	6.1698188	2.9747065	0.6364267
C	4.5353944	2.3457105	-0.9994264
H	4.7065663	2.6359906	-2.0457949
O	6.8317457	3.6852806	1.3856330
C	4.7168878	4.7940524	-0.2030560
H	4.4785563	5.0287401	-1.2474292
H	5.6067237	5.3721791	0.0702446
N	5.0830342	3.3860345	-0.1121163
C	3.0488965	2.0756489	-0.8187705
C	2.2122028	2.0246117	-1.9385224
C	2.5002824	1.8442027	0.4506479
C	0.8538359	1.7514066	-1.8040079
H	2.6273845	2.2016041	-2.9275085
C	1.1462003	1.5743290	0.5983284
H	3.1363082	1.8864328	1.3301127
C	0.2996317	1.5238504	-0.5300912
H	0.2136454	1.7168373	-2.6801232
H	0.7287564	1.4004835	1.5852865
N	5.3321051	-0.0567673	-1.1572584
C	6.0490261	-2.3795133	-1.2884676
H	6.5354876	-2.4281184	-2.2725303
H	6.5920378	-3.0688908	-0.6353779

C	4.6066248	-2.8294804	-1.4210238
C	3.9381976	-2.7791173	-2.6503483
C	3.9168946	-3.3198242	-0.3029481
C	2.6132430	-3.2061031	-2.7625149
H	4.4611683	-2.4037144	-3.5268417
C	2.5920905	-3.7450273	-0.4098235
H	4.4241471	-3.3699154	0.6582883
C	1.9353265	-3.6900753	-1.6418096
H	2.1110520	-3.1608100	-3.7257829
H	2.0735635	-4.1233389	0.4677309
H	0.9041252	-4.0229476	-1.7270925
C	9.1797454	-2.0507759	-0.4091184
C	8.7851620	-1.5496148	1.9193916
C	9.8931796	-3.3296914	0.0185635
H	9.9192164	-1.2383712	-0.5187759
H	8.7054192	-2.2041394	-1.3817542
C	9.4902520	-2.8550788	2.2790675
H	9.5261365	-0.7318734	1.9442653
H	8.0095941	-1.3323952	2.6612336
H	10.7113112	-3.5559831	-0.6721198
H	9.1839792	-4.1734406	0.0112877
H	10.0132191	-2.7516790	3.2346105
H	8.7492840	-3.6659565	2.3673260
N	8.1622757	-1.6894305	0.5974239
O	10.4773373	-3.1949194	1.3102921
C	-1.0918031	1.2575602	-0.3791261
C	-2.2816920	1.0376669	-0.2444314
C	-3.6736555	0.7865784	-0.0854143
C	-4.5212643	0.6891890	-1.2084082
C	-4.2337875	0.6318045	1.1995503
C	-5.8789512	0.4413425	-1.0494689
H	-4.1052539	0.8148199	-2.2030001
C	-5.5946980	0.4008486	1.3538852
H	-3.5911183	0.6928680	2.0721715
C	-6.4398864	0.2983321	0.2332412
H	-6.5190460	0.3858532	-1.9242420
H	-6.0058430	0.2686680	2.3493724
C	-7.8893656	0.0457693	0.4029851
C	-8.6545843	0.8694220	1.2508203
C	-8.5055747	-1.0238504	-0.2744364
C	-8.3047044	2.0308374	1.9891300
H	-7.3236825	2.4831649	2.0203902
C	-9.4543417	2.4712395	2.6348960
H	-9.5563954	3.3291540	3.2856881
C	-10.4864016	1.5861689	2.2728295
H	-11.5301337	1.6020367	2.5590639

N	-10.0159173	0.6334125	1.4490524
N	-9.8707104	-1.2772828	-0.1301932
C	-7.9634467	-2.0366129	-1.1093230
H	-6.9240404	-2.1292547	-1.3904187
C	-9.0092260	-2.8792338	-1.4682677
H	-8.9607093	-3.7578869	-2.0971635
C	-10.1652317	-2.3803223	-0.8402288
H	-11.1755752	-2.7677277	-0.8642044
B	-10.8660862	-0.4717920	0.7558146
F	-11.4435263	-1.3090198	1.7066667
F	-11.8551367	0.1120751	-0.0323944
C	3.5985694	5.1959681	0.6627983
C	2.6889844	5.5625444	1.3679387
H	1.8804510	5.8722817	1.9948759

Excited State 1: Singlet-A 2.5723 eV 482.00 nm f=0.3578 <S**2>=0.000

184 -> 187 0.45648

186 -> 187 0.53474

Excited State 21: Singlet-A 4.3743 eV 283.44 nm f=0.1079 <S**2>=0.000

186 -> 190 0.65483

4.8 Main orbital contributions for the longest wavelength absorption computed in $CHCl_3$ and PhMe

Table S3. Selected TD-DFT absorption maxima in $CHCl_3$ and main orbital contributions[A]

Compound	$\lambda_{max\ abs}$ (nm) Theoretical	Oscillator strength (*f*)	Main contributions
**11a**	472	0.490	HOMO-2→LUMO (40%)<br>HOMO→LUMO (60%)
**11b**	470	0.468	HOMO-2→LUMO (46%)<br>HOMO→LUMO (54%)
**11c**	468	0.468	HOMO-2→LUMO (45%)<br>HOMO→LUMO (55%)
**11d**	479	0.379	HOMO-3→LUMO (20%)<br>HOMO-1→LUMO (20%)<br>HOMO→LUMO (60%)
**11e**	470	0.501	HOMO-2→LUMO (40%)<br>HOMO→LUMO (60%)
**11f**	469	0.216	HOMO-3→LUMO (19%)<br>HOMO-1→LUMO (19%)

			HOMO→LUMO (62%)
**11g**	470	0.488	HOMO-2→LUMO (42%)<br>HOMO→LUMO (58%)

[A] calculated at the B3LYP/6-31G(d) level of theory.

Table S4. Selected TD-DFT absorption maxima in PhMe and main orbital contributions[A]

	**$\lambda_{max\ abs}$ (nm)**		
**Compound**	**Theoretical**	**Oscillator strength (*f*)**	**Main contributions**
**11a**	469	0.519	HOMO-2→LUMO (29%)<br>HOMO→LUMO (71%)
**11b**	467	0.504	HOMO-2→LUMO (41%)<br>HOMO→LUMO (59%)
**11c**	485	0.327	HOMO-2→LUMO (36%)<br>HOMO→LUMO (64%)
**11d**	476	0.393	HOMO-3→LUMO (11%)<br>HOMO-1→LUMO (41%)<br>HOMO→LUMO (48%)
**11e**	467	0.532	HOMO-2→LUMO (25%)<br>HOMO→LUMO (75%)
**11f**	463	0.491	HOMO-3→LUMO (46%)<br>HOMO-1→LUMO (54%)
**11g**	468	0.511	HOMO-2→LUMO (23%)<br>HOMO→LUMO (77%)

[A] calculated at the B3LYP/6-31G(d) level of theory.

Table S5. Computed frontier molecular orbitals of **11a-g** in $CHCl_3$

**11a**	HOMO<br><br>-5.761 eV	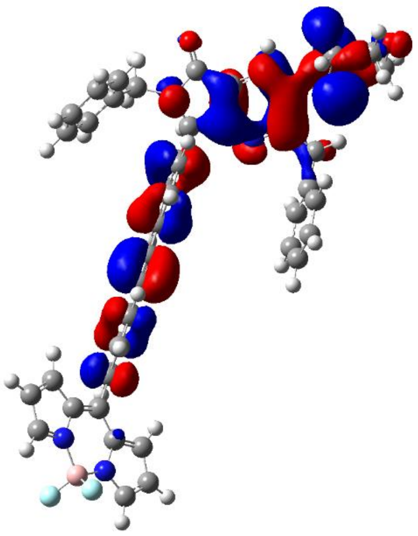	LUMO<br><br>-2.793 eV	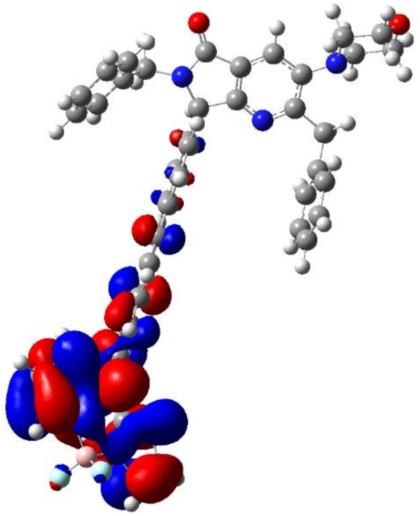

**11b**	HOMO<br><br>-5.750 eV	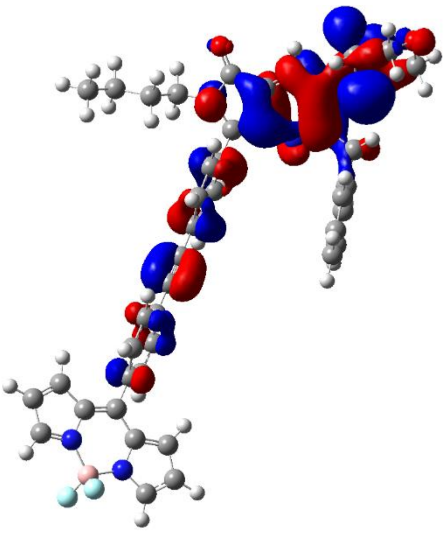	LUMO<br><br>-2.797 eV	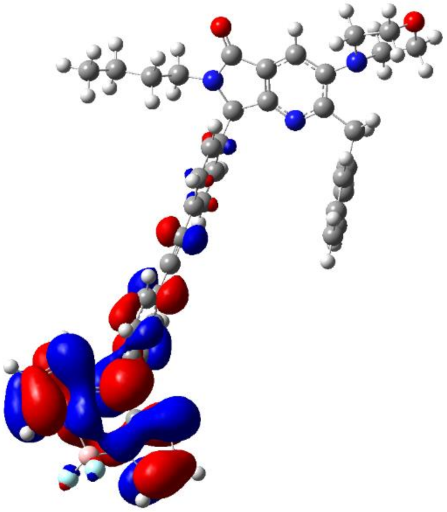
**11c**	HOMO<br><br>-5.760 eV	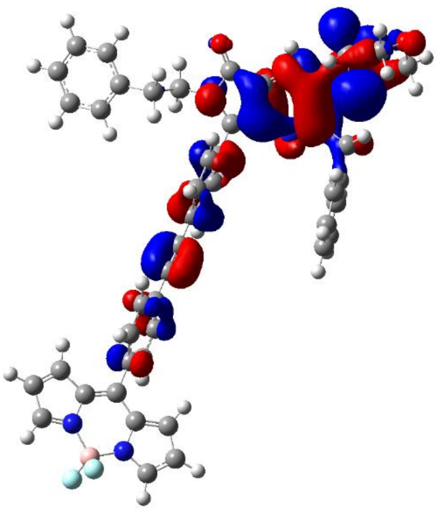	LUMO<br><br>-2.798 eV	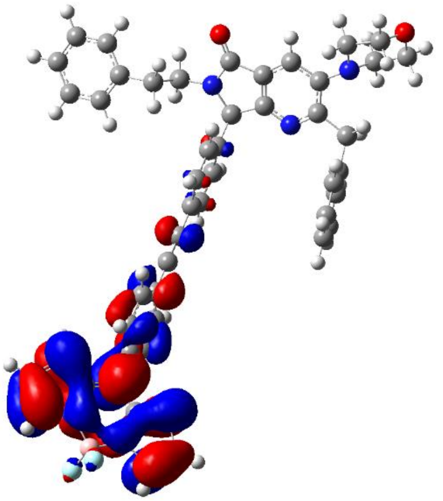

**11d**	HOMO<br><br>-5.661 eV	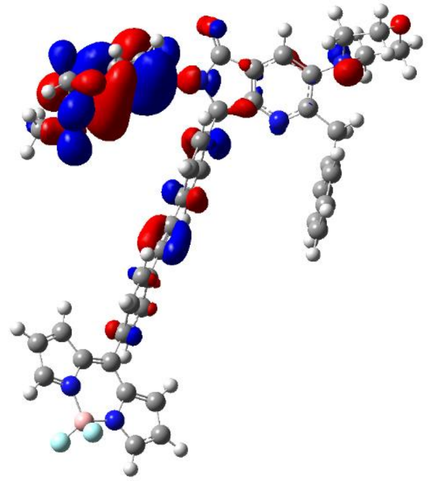	LUMO<br><br>-2.777 eV	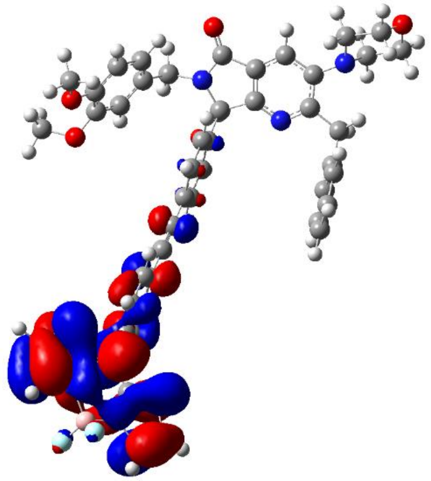
**11e**	HOMO<br><br>-5.774 eV	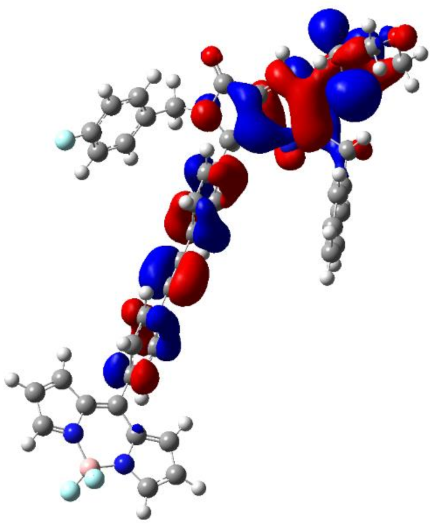	LUMO<br><br>-2.793 eV	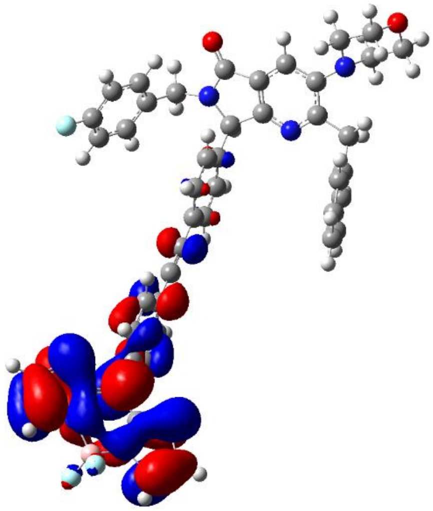
**11f**	HOMO<br><br>-5.673 eV	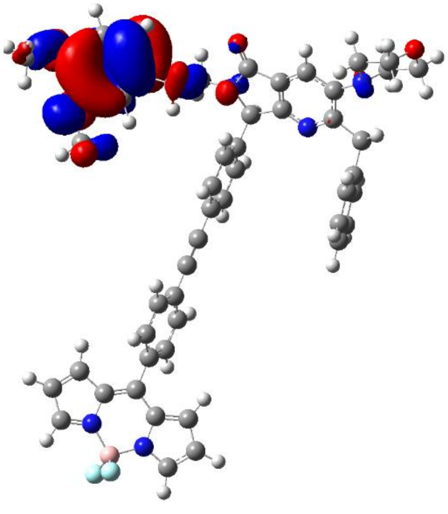	LUMO<br><br>-2.811 eV	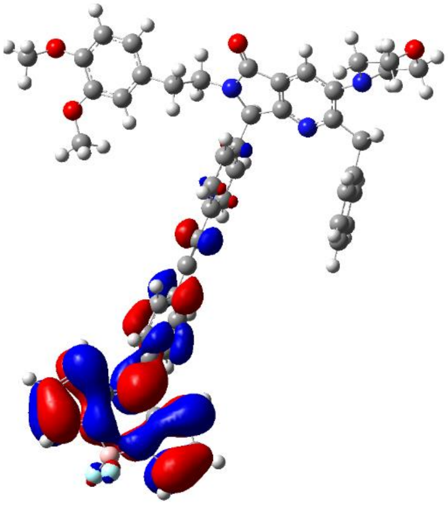

**11g**	HOMO<br><br>-5.776 eV	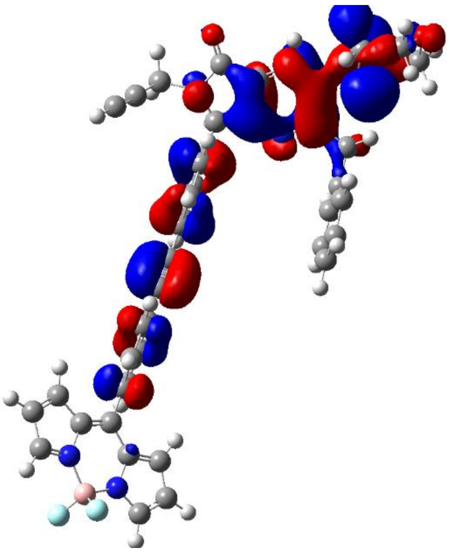	LUMO<br><br>-2.793 eV	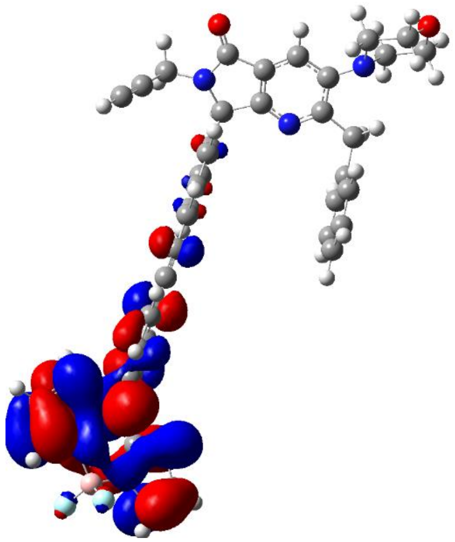

Table S6. Computed frontier molecular orbitals of **11a-g** in PhMe

**11a**	HOMO<br><br>-5.810 eV	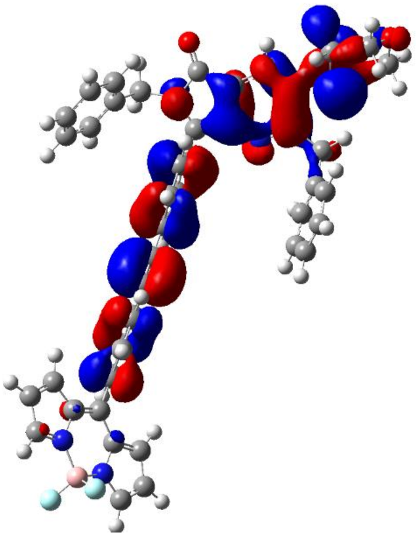	LUMO<br><br>-2.795 eV	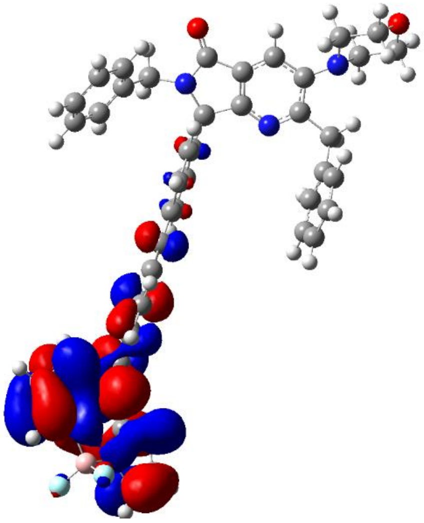

**11b**	HOMO<br><br>-5.802 eV	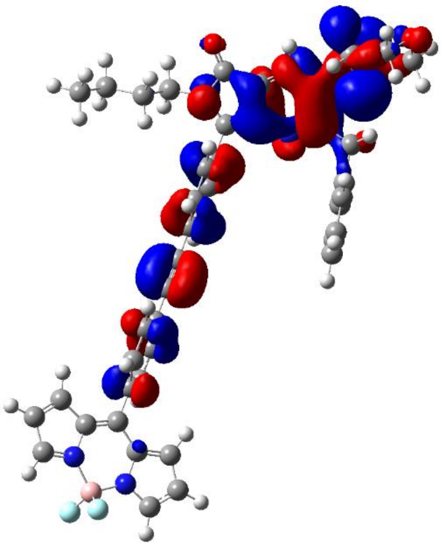	LUMO<br><br>-2.800 eV	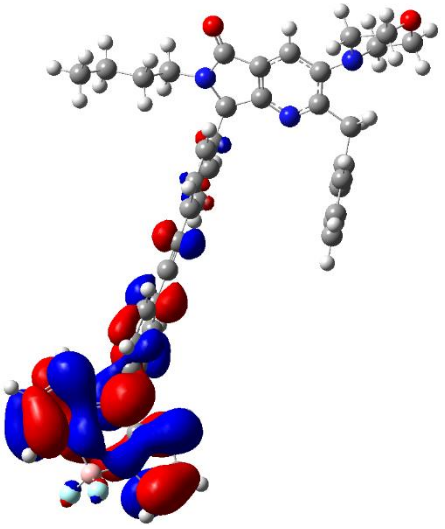
**11c**	HOMO<br><br>-5.817 eV	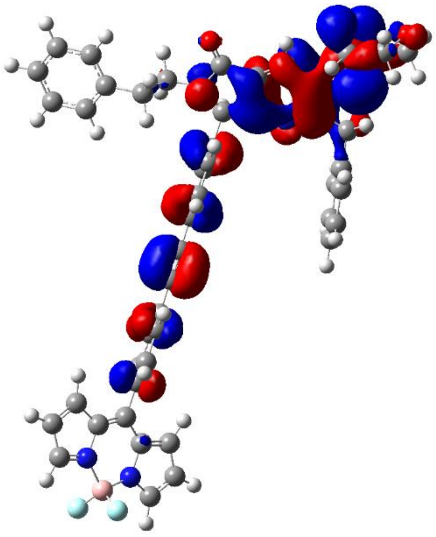	LUMO<br><br>-2.798 eV	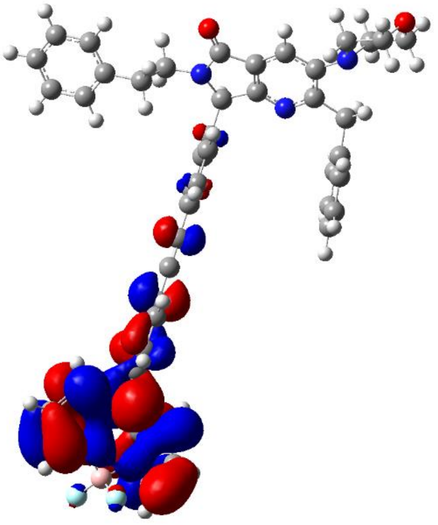
**11d**	HOMO<br><br>-5.658 eV	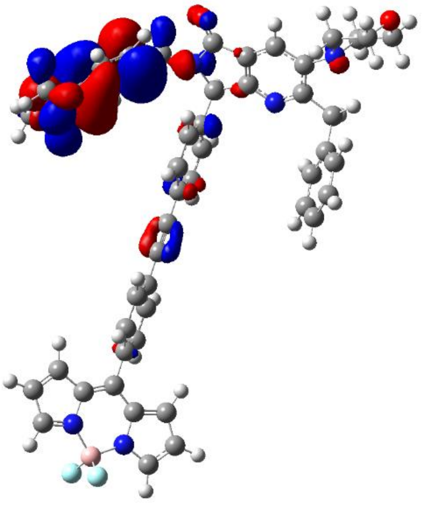	LUMO<br><br>-2.770 eV	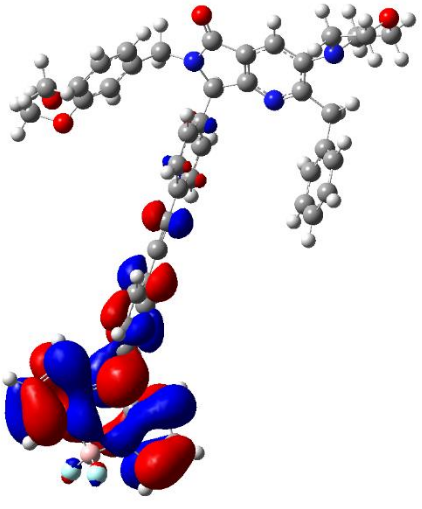

**11e**	HOMO<br><br>-5.824 eV	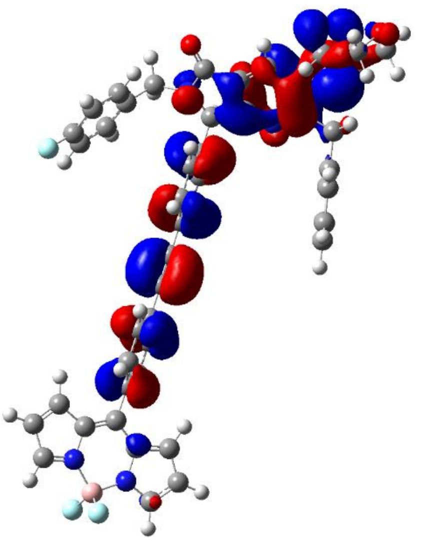	LUMO<br><br>-2.792 eV	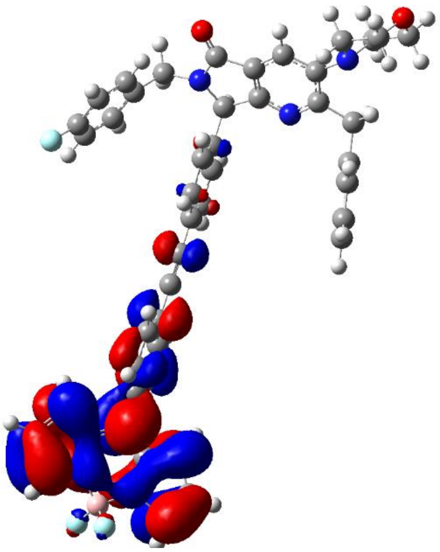
**11f**	HOMO-1<br><br>-5.827 eV	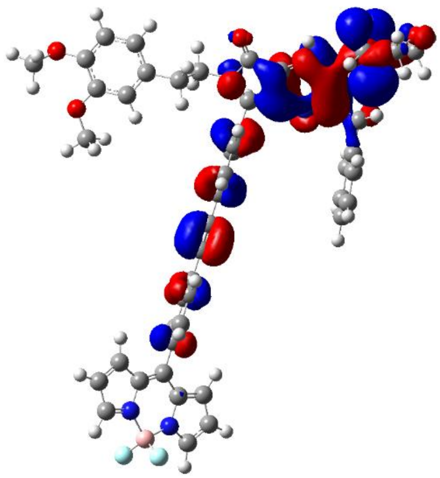	LUMO<br><br>-2.818 eV	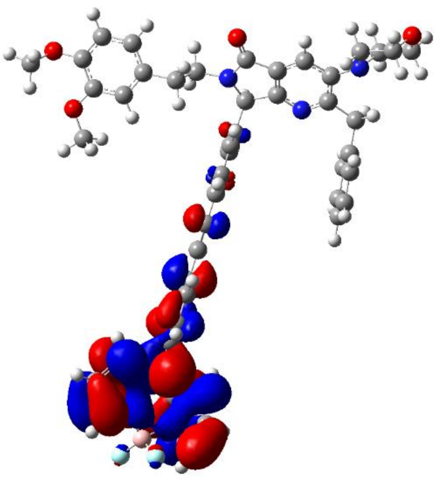

**11g**	HOMO<br><br>-5.823 eV	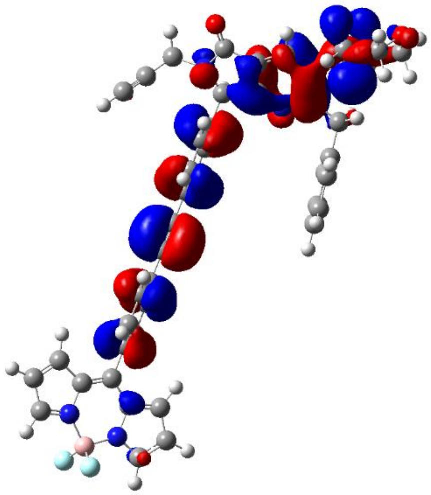	LUMO<br><br>-2.791 eV	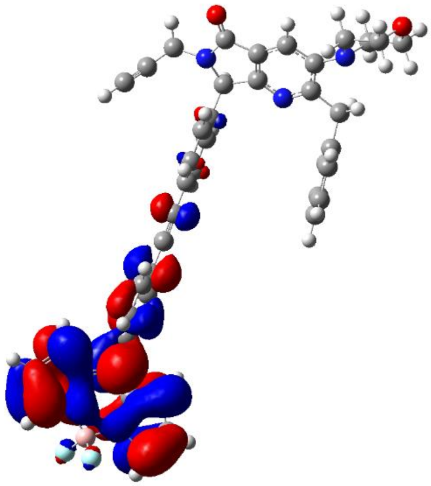

5 References

1. Xochitiotzi-Flores, E., Islas-Mejía, A., García-Ortega, H., Romero-Avila, M., Mendez-Stivalet, J.M., Carreón-Castro, M., Santillan, R., Maldonado-Domínguez, M., Arcos-Ramos, R., Farfán, N. (2016) On the structure of *meso*-substituted F-BODIPYs and their assembly in molecular crystals: An experimental-theoretical approach. *J. Organomet. Chem.*, 805.148-157. https://doi.org/10.1016/j.jorganchem.2016.01.021.
2. Fayol, A., Housseman, C., Sun, X., Janvier, P., Bienaymé, H., Zhu, J. (2005) Synthesis of α-isocyano-α-alkyl(aryl)acetamides and their use in the multicomponent synthesis of 5-aminooxazole, pyrrolo[3,4-*b*]pyridin-5-one and 4,5,6,7-tetrahydrofuro[2,3-*c*]pyridine. *Synthesis*, 1, 0161–0165. https://doi.org/10.1055/s-2004-831225.
